# Characterization of *Pseudomonas aeruginosa* Quorum Sensing Inhibitors from the Endophyte *Lasiodiplodia venezuelensis* and Evaluation of Their Antivirulence Effects by Metabolomics

**DOI:** 10.3390/microorganisms9091807

**Published:** 2021-08-25

**Authors:** Léonie Pellissier, Sara Leoni, Laurence Marcourt, Emerson Ferreira Queiroz, Nicole Lecoultre, Luis-Manuel Quiros-Guerrero, Morgane Barthélémy, Véronique Eparvier, Jérôme Chave, Didier Stien, Katia Gindro, Karl Perron, Jean-Luc Wolfender

**Affiliations:** 1 School of Pharmaceutical Sciences, University of Geneva, CMU-Rue Michel-Servet 1, CH-1211 Geneva 4, Switzerland; Laurence.Marcourt@unige.ch (L.M.); Emerson.Ferreira@unige.ch (E.F.Q.); Luis.Guerrero@unige.ch (L.-M.Q.-G.); 2 Institute of Pharmaceutical Sciences of Western Switzerland, University of Geneva, CMU-Rue Michel Servet 1, CH-1211 Geneva 4, Switzerland; 3 Microbiology Unit, Department of Botany and Plant Biology, University of Geneva, CMU-Rue Michel-Servet 1, CH-1211 Geneva 4, Switzerland; Sara.Leoni@unige.ch (S.L.); karl.perron@unige.ch (K.P.); 4 Mycology Group, Research Department Plant Protection, Agroscope, Route de Duillier 50, 1260 Nyon, Switzerland; nicole.lecoultre@agroscope.admin.ch (N.L.); katia.gindro@agroscope.admin.ch (K.G.); 5 Institut de Chimie des Substances Naturelles, Université Paris-Saclay, CNRS, UPR 2301, 91198 Gif-sur-Yvette, France; morgane.barthelemy@gmail.com (M.B.); veronique.eparvier@cnrs.fr (V.E.); 6 Laboratoire Evolution et Diversité Biologique (UMR 5174), CNRS, UT3, IRD, Université Toulouse 3, 118 Route de Narbonne, 31062 Toulouse, France; jerome.chave@univ-tlse3.fr; 7 Sorbonne Université, CNRS, Laboratoire de Biodiversité et Biotechnologie Microbiennes, LBBM, Observatoire Océanologique, 66650 Banyuls-Sur-Mer, France; didier.stien@cnrs.fr

**Keywords:** natural products, quorum sensing inhibition, *Pseudomonas aeruginosa*, endophytic fungi, *Lasiodiplodia venezuelensis*, bio-guided fractionation, secondary metabolites, molecular networking, virulence factors, metabolomics

## Abstract

The opportunistic pathogen *Pseudomonas aeruginosa* is one of the “critical priority pathogens” due to its multidrug resistance to a wide range of antibiotics. Its ability to invade and damage host tissues is due to the use of quorum sensing (QS) to collectively produce a plethora of virulence factors. Inhibition of QS is an attractive strategy for new antimicrobial agents because it disrupts the initial events of infection without killing the pathogen. Highly diverse microorganisms as endophytes represent an under-explored source of bioactive natural products, offering opportunities for the discovery of novel QS inhibitors (QSI). In the present work, the objective was to explore selective QSIs within a unique collection of fungal endophytes isolated from the tropical palm *Astrocaryum sciophilum*. The fungi were cultured, extracted, and screened for their antibacterial and specific anti-QS activities against *P. aeruginosa*. The endophytic strain *Lasiodiplodia venezuelensis* was prioritized for scaled-up fractionation for its selective activity, leading to the isolation of eight compounds in a single step. Among them, two pyran-derivatives were found to be responsible for the QSI activity, with an effect on some QS-regulated virulence factors. Additional non-targeted metabolomic studies on *P. aeruginosa* documented their effects on the production of various virulence-related metabolites.

## 1. Introduction

Antibiotics remain a critical element for the treatment of bacterial diseases. However, they have been so widely used that many are losing their capacity to fight bacteria [1,2]. Antimicrobial resistance, AMR, is considered as one of the biggest threats to public health today, with estimated deaths projected to reach millions by 2050 [3,4]. This crisis is related to the emergence of multidrug resistant pathogens, as the so-called “ESKAPE” pathogens (*Enterococcus faecium, Staphylococcus aureus, Klebsiella pneumoniae, Acinetobacter baumannii, Pseudomonas aeruginosa* and *Enterobacter sp.)* [5]. Concurrently, the pace of development of new antimicrobials has slowed down in the past 20 years and cannot keep up with the accelerated evolution of AMR. Few novel classes of antibiotics have been discovered, and only one out of sixteen antibiotics in early-stage research reaches the clinical application. Therefore, there is an urgent need to discover new antimicrobial compounds and develop therapies that will ensure long term efficacy against pathogens. Antivirulence is an alternative strategy targeting the components of the bacteria that are responsible for pathogenesis, instead of those that are essential for growth. Such components, known as virulence factors, are synthesized specifically to colonize and impair the host. Antivirulence aims to disarm rather than kill the pathogen [6]. One of the most studied targets for antivirulence therapy is bacterial cell-to-cell communication called quorum sensing (QS), a key control center for the virulence of many pathogens of plants, animals and humans [7].

As stressed by the WHO, the opportunistic pathogen *Pseudomonas aeruginosa* is one of the “critical priority pathogens” because of its multidrug resistance to a wide range of antibiotics [8]. This gram-negative bacterium causes severe infections both in hospitals and the community, and is a main pathogen in cystic fibrosis and immunocompromised patients [9]. Its ability to invade and damage the host tissues is due to the use of QS to collectively produce a plethora of virulence factors. This includes secretion of degrading toxins (exoenzymes, exotoxins), proteases and hemolysins (phospholipase, rhamnolipids) [10]. During chronic infections, QS is also involved in the formation of biofilms, highly tolerant to antibiotics [11]. *P. aeruginosa* harbors three QS systems: *las*, *rhl* that works in series, and *pqs*. All are interconnected but each system is autoregulated and also modulates the activities of the others. The *las* and *rhl* systems rely on two different *N*-acyl-homoserine lactones (AHL)-type signal molecules, *N*-(3-oxododecanoyl)-homoserine lactone (3OC12HSL) and *N*-butanoyl-L-homoserine lactone (C4HSL) [12], respectively, of which the production is catalyzed by the autoinducer (AI) synthases LasI and RhlI [13]. At high cell density, the related AHL (3OC12HSL) AI binds LasR. The complex formed binds to promoter elements, activating the transcription of genes encoding for virulence factors involved in the initiation of the infection process (elastases, proteases, exotoxin A) [13,14]. This system also activates the expression of *rhlI* that will synthesize the AI (C4HSL). C4HSL then binds RhlR and this complex induces the transcription of a second class of specific genes encoding notably for elastases, proteases, pyocyanin, siderophores, hemolysin and rhamnolipids [15]. The third circuit, *pqs*, employs 2-alkyl-4-quinolones: 2-heptyl-4-hydroxyquinoline (HHQ) or 4,2-heptyl-3-hydroxy-4(1H)-quinolone (PQS) that are non-AHL signals detected by the regulator MvfR (PqsR). The complex auto-induces the synthesis of PQS by activating the transcription of the *pqsABCDE* operon and the expression of a set of virulence factors such as elastase, pyocyanin, lectins and hydrogen cyanide regulation. The *pqs* QS system further activates *rhlI* and *rhlR* expression, and constitutes an additional link between the *las* and the *rhl* circuits [16,17]. Thus, MvfR plays a critical role in chronic infection and in the development of antibacterial resistance. Due to the central role of QS in *P. aeruginosa* virulence, and according to the fact that mutations in their key genes strongly reduces the pathogenicity, *las*, *rhl* and *pqs* circuits constitute attractive targets for novel antimicrobial agents [18]. Bioprospection for natural products has become an established way for the discovery of new drugs, notably via the isolation of bioactive molecules from living organisms. In this perspective, research efforts have turned more and more towards microorganisms residing within plants, known as endophytes [19]. Endophytes are described as a group of microorganisms (mostly bacteria or fungi) that inhabit, during all or part of their life cycle, apparently healthy plant tissues without causing any symptoms [20]. They are ubiquitous as they have been found in very various and biodiverse ecosystems, and every plant species studied so far have been found to host at least one endophyte but could potentially host hundreds of endophytes [21]. Endophytes have shown the potential to produce a vast number of biologically active metabolites as antimicrobials, antifungals, anticancer, immunosuppressants among others [22,23]. The co-evolution and mutualistic relationship of endophytes with their host plants from the time they emerged on land might have led to the co-evolution of some functional traits, such as aiding plants to resist environmental stresses and the production of bioactive metabolites [24]. During coexistence in this ecological niche, endophytes encounter invasion by a multitude of specific or generalist pathogens for which QS is essential for the colonization, pathogenesis and development of AMR [19,25]. One of their strategies to prevent the colonization of the plant tissues by pathogenic organisms is therefore by impairing the QS between invading pathogens, i.e., quorum quenching (QQ) [26]. Endophytes disrupt bacterial cross-talk as a natural process, either by possessing QQ enzymes (lactonases and acylases) [27] or by producing their own QQ chemicals, such as compounds mimicking the microbial AIs [28]. For instance, the endophytic fungus *Penicillium restrictum* isolated from *Silybum marianum* plant produces polyhydroxyantrhaquinones with potent QQ activity in MRSA [29]. Similarly, *Aspergillus flavipes* isolated from a mangroves plant *Acanthus ilicifoilius* produces flavipesins A that demonstrate anti-biofilm activities in *S. aureus* [30]. Regarding the vast opportunities for the discovery of new quorum sensing inhibitors they offer, we investigated cultivated communities of endophytic fungi isolated from a model plant, *Astrocaryum sciophilum* (Miq.) Pulle, known for its long-life cycle and its steady association with microbial communities over many years. *A. sciophilum* is a solitary, understory palm endemic to the northeast region of the Amazon basin (Amapá and Pará states of Brazil, French Guiana, Guyana and Suriname) [31]. With its noteworthy maturation age of approximately 170 years and leaves that can be up to 20 years old, this palm is considered as long-lived and particularly resistant [32]. This implies that its associated endophytes manage to survive and resist the internal and external plant environment for a substantial period. To this extent, they may have developed antipathogen and antivirulence strategies involved in their longevity and that of their host [33]. Furthermore, previous work on another collection of endophytes associated with that plant model has shown that it contained antimicrobial strains and bioactive compounds [34,35].

In the present study, a collection of fungi isolated as cultivable endophyte strains from the leaves of *A. sciophilum* were cultured, extracted and screened for their antibacterial activity and their specific anti-QS activity against *P. aeruginosa*. Among them, the endophytic strain *Lasiodiplodia venezuelensis* (Burgess, Barber and Mohali [36]) was prioritized for scaled-up fractionation for its selective activity and taxonomical originality. The genus *Lasiodiplodia* belongs to the phylum Ascomycota, class Dothideomycetes, order Botryosphaeriales, and family Botryiosphaeriaceae [37]. Members of this family have been found on a large variety of plant hosts as important pathogens of several woody perennial plants, mostly in tropical and subtropical regions [38,39,40,41]. They can also survive as saprophytes or endophytes within seeds and living tissues, in a latent phase without causing any symptoms [42]. 

In the frame of the present study, we isolated and characterized the metabolites responsible for *P. aeruginosa* quorum sensing inhibition in *L. venezuelensis* and documented their biological activity.

## 2. Materials and Methods

### 2.1. General Experimental Procedures

NMR spectroscopic data were recorded on a Bruker Avance Neo 600 MHz NMR spectrometer equipped with a QCI 5 mm Cryoprobe and a SampleJet automated sample changer (Bruker BioSpin, Rheinstetten, Germany). Chemical shifts are reported in parts per million (δ) using the residual CD_3_OD (δ_H_ 3.31; δ_C_ 49.0) or DMSO-*d*_6_ (δ_H_ 2.50; δ_C_ 39.5) as internal standards for ^1^H and ^13^C NMR, respectively, and coupling constants (*J*) are reported in Hertz (Hz). Complete assignments were obtained based on 2D-NMR experiments: COSY, ROESY, edited-HSQC and HMBC. HRESIMS data were obtained on a Q Exactive Focus mass spectrometer (Thermo Scientific, Bremen, Germany), using a heated electrospray ionization (HESI-II) source. Analytical HPLC was carried out on a Acquity HP 1260 (Waters, Milford, MA, USA) system equipped with a photodiode array and an ELSD detector (Sedere, Alfortville, France). Semi-preparative HPLC-UV analyses were conducted on a Shimadzu system (Kyoto, Japan) equipped with an LC-20A module pump, an SPD-20A UV/VIS, a 7725I Rheodyne^®^ valve and an FRC-10A fraction collector. The system is controlled by the software LabSolutions (v5.97, Shimadzu, Kyoto, Japan, 2019). 

### 2.2. Electronic Circular Dichroism (ECD) and Optical Rotation (αD)

The spectra obtained by electronic circular dichroism (ECD) and UV were analyzed on a J-815 circular dichroism spectrometer (Loveland, CO, USA). The solvent used was methanol. The cell temperature was 24.5 °C and stabilised using a thermostatted ED circulation bath (Julabo, Seelbach, Germany) and a PFD-350S thermostat (Jasco, Tokyo, Japan). The set scan speed was 200 nm/min in continuous mode with a scan start at 800 nm and an end at 200 nm. The optical rotations were measured in methanol solutions on P-1030 polarimeter (Jasco, Tokyo, Japan) in a 1 cm tube.

### 2.3. Plant Material

*Astrocaryum sciophilum* palm tree leaves were sampled in August 2017 at the Nouragues ecological research station in French Guiana. Leaf samples were collected at the Inselberg research station (4°05′ N-52°41′ W) by randomly cutting 5 × 3 cm pieces of fresh leaflets and rachis from individual plants using sterile tweezers. The pieces were placed in Eppendorf tubes containing Potato Dextrose Broth 1/4 (PDB, Nutriselect™ Basic, Sigma-Aldrich, Germany) medium to allow regrowth of the fungi, as well as tubes containing Cetyl trimethylammonium bromide H6269 (CTAB, CAS: 57-09-0, Sigma-Aldrich, Germany) to preserve the genetic material. Petri dishes containing “Potato Dextrose Agar” (PDA, Potato Dextrose Agar, Sigma-Aldrich, Germany), medium and aureomycin were also left open on the stipe in order to sample fungi from the environment close to the palm tree. These media were enriched with aureomycin to avoid the growth of bacteria and promote the growth of fungi only.

### 2.4. Isolation of Endophytes

The isolation of the fungi and the constitution of the strain collection were carried out at the Agroscope in Changins (Federal Department of Economy, Education and Research DEFR, Plant Protection Research Division). The surface of the plant fragments was thoroughly washed for three hours under a stream of tap water in a suitable container, then washed in three successive baths of sterile water for 10 min each under a laminar flow hood. This non-chemical surface cleaning method was used to reduce possible loss of diversity due to aggressive sterilization using alcoholic solutions or even Paraquat (82). The surface-sterilized leaves were aseptically cut into small segments (0.5 cm^2^), placed individually in Petri dishes containing PDA, amended with aureomycin (25 ppm.L^−1^) in 9 cm Petri dishes and cultured at room temperature. Each individual emerging hypha fragment was removed and placed in 9 cm Petri dishes containing PDA and aureomycin. The isolated and selected strains were then individually cultured on a small scale in 9 cm Petri dishes containing PDA medium without aureomycin. Each strain has been integrated into the dynamic fungal library of Agroscope, whose content is available on the web (database Mycoscope: www.mycoscope.ch, accessed on 20 February 2021), in vials containing 5 mL of diluted PDB aqueous solution (1:4) at 4 °C.

### 2.5. Identification of Fungal Strains

Identification of the isolated strains was performed by extraction of total DNA (phenol/chloroform), PCR amplification of specific ribosomal DNA regions, ITS1F and ITS4, and sequencing (Fasteris, Geneva, Switzerland). The obtained sequences were then submitted to BLAST on NCBI (https://blast.ncbi.nlm.nih.gov/Blast.cgi, accessed on 20 February 2021) to identify the strain. The specimens were then entered in the dynamic mycotheca of Agroscope and registered in the fungal database (www.mycoscope.ch, accessed on 20 February 2021) under the reference numbers 1883 to 2024.

### 2.6. Culture and Extraction

Each strain was cultivated at 21 °C under alternating light and dark (12 h each) in 10 cm Petri dishes containing PDA culture medium. The culture medium was then extracted by maceration in ethyl acetate (EtOAc, Thermo fisher scientific, Waltham, MA, USA) for 24 h at room temperature under agitation. The organic phase was recovered by vacuum filtration and washed three times with Millipore Corporation (MilliQ) water (Elga LabWater, High Wycombe, UK). The organic and water phases were dried under reduced pressure with a rotary evaporator (Büchi, Flawil, Switzerland) to yield crude mixtures. Agar from uninoculated PDA plates were used as controls.

### 2.7. Scale Up of the Fungal Culture of Lasiodiplodia Venezuelensis

Strains were cultivated during 15 days at room temperature in 14 cm Petri dishes containing PDA medium. Culture media was then consecutively extracted three times by maceration in EtOAc for 24 h at room temperature under agitation (After each 24-h maceration, the organic phase was harvested and the medium was macerated again in renewed EtOAc). The combined organic solution was washed three times with MilliQ water. The organic and water phases were dried under reduced pressure with a rotary evaporator to yield the ethyl acetate fraction (Et) and the water fraction (W). The Et fraction was then degreased by liquid–liquid partition in a hexane/methanol–water (1/1) system with methanol–water (7:3) to give the hexane (EtH) and the methanol–water fraction (EtM). Large scale cultivation of the *Lasiodiplodia venezuelensis* strain was conducted on 100 14 cm Petri dishes to yield the factions A02Et (1 g) and A02W (335 mg). Six hundred mg of the fraction A02Et were then degreased to yield fractions A02EtH (320 mg) and A02EtM (250 mg).

### 2.8. HPLC-PDA-ELSD Analyses

HPLC-PDA analyses were conducted as follow: Waters^®^ X-bridge C18 column (250 × 4.6 mm i.d., 5 μm) (Waters^®^, Milford, MA, USA) equipped with a Waters^®^ C18 pre-column cartridge holder (20 × 4.6 mm i.d.); solvent system H_2_O (A) and MeOH (B), both containing 0.1% formic acid (FA). The column was equilibrated with 3% of B during 15 min. The separation was performed with an isocratic mode at 3% over 15 min, then 5% over 5 min, followed by a gradient mode from 5% to 42% over 40 min, then from 42 to 100% of B over 5 min. The column was then washed with 100% of B during 10 min and equilibrated with 3% of B over 15 min. The flow rate was 1 mL/min, the injection volume 20 μL and the sample concentration 1 mg/mL in MeOH. The UV absorbance was measured at 254 nm and 280 nm and the ELSD set at 45 °C, 3.5 bar N_2_, gain 8.

### 2.9. Semi-Preparative HPLC-UV Purification of the Water Partition of the Strain Lasiodiplodia Venezuelensis (A02W)

Semi-preparative HPLC-UV analyses were conducted as follow: Waters^®^ X-bridge C18 column (250 × 19 mm i.d., 5 μm) equipped with a Waters^®^ C18 pre-column cartridge holder (10 × 4.6 mm i.d); solvent system H_2_O (A) and MeOH (B), both containing 0.1% FA. The column was equilibrated with 3% of B during 15 min. The separation was performed with an isocratic mode at 3% over 15 min, then 5% over 5 min, followed by a gradient mode from 5% to 42% over 40 min, then from 42 to 100% of B over 5 min. The column was then washed with 100% of B during 10 min. The flow rate was 17 mL/min. These conditions were calculated from a geometric gradient transfer from the analytical HPLC using the software developed by Guillarme et al. [43]. The UV traces were recorded at 254 and 280 nm. The HPLC-UV chromatograms of the different separation are shown. The A02W partition was injected in the semi-preparative HPLC-UV using dry load (10, 50, 50, 50 mg). The dry load injection was performed using a dry load cell, i.e., a commercial small aluminum pre-column cartridge from Waters^®^ (10 × 19 mm i.d.) that was opened on its side. The extract was mixed with the same ratio of stationary phase (C18 Zeoprep^®^ 40–63 μm), and this mixture was transferred in the dry load cell as a thin layer at the bottom of the cell. That thin layer was then covered with stationary phase until reaching a final mass of 1000 mg and the cell was closed with a metallic sinter. The dry load was then directly connected to the Rheodyne^®^ valve. According to UV signals, 19 fractions were collected. All the fractions were then analyzed by UHPLC-UV-ELSD-QDa.

### 2.10. Description of the Isolated Compounds

Uridine (1) (F3): ^1^H NMR (CD_3_OD, 600 MHz) δ 3.73 (1H, dd, *J* = 12.2, 3.1 Hz, H-5’b), 3.84 (1H, dd, *J* = 12.2, 2.7 Hz, H-5’a), 4.00 (1H, dt, *J* = 4.7, 3.1, 2.7 Hz, H-4’), 4.14 (1H, t, *J* = 4.7 Hz, H-3’), 4.18 (1H, t, *J* = 4.7 Hz, H-2’), 5.69 (1H, d, *J* = 8.1 Hz, H-5), 5.90 (1H, d, *J* = 4.7 Hz, H-1’), 8.01 (1H, d, *J* = 8.1 Hz, H-6); ^13^C NMR (CD_3_OD, 151 MHz) δ 61.9 (C-5’), 71.0 (C-3’), 75.4 (C-2’), 86.1 (C-4’), 90.3 (C-1’), 102.3 (C-5), 142.4 (C-6), 152.2 (C-2), 165.9 (C-4). ESI(+)-HRMS *m*/*z* 245.0770 [M + H]^+^ (calcd. for C_9_H_13_N_2_O_6_^+^, 245.0768, Δppm = 0.76). For NMR spectra, see Figures S1–S5 [44].

(5S,6S)-6-((3’S,4’S,Z)-3’,4’-Dihydroxypent-1-en-1-yl)-5-hydroxy-5,6-dihydro-2H-pyran-2-one (2) (F4): ${\left[a\right]}_{D}^{25}$ 277.7 (c. 0.15, MeOH); UV(MeOH) λ_max_ 226 (3.7) nm. ^1^H NMR (CD_3_OD, 600 MHz) δ 1.17 (2H, d, *J* = 6.4 Hz, H-5’), 3.74 (1H, qd, *J* = 6.4, 4.7 Hz, H-4’), 4.21 (1H, dd, *J* = 5.6, 2.9 Hz, H-5), 4.24 (1H, ddd, *J* = 8.2, 4.7, 1.0 Hz, H-3’), 5.35 (1H, ddd, *J* = 8.7, 2.9, 0.9 Hz, H-6), 5.85 (1H, ddd, *J* = 11.4, 8.2, 0.9 Hz, H-2’), 5.93 (1H, ddd, *J* = 11.4, 8.7, 1.0 Hz, H-1’), 6.08 (1H, d, *J* = 9.8 Hz, H-3), 7.07 (1H, dd, *J* = 9.8, 5.6 Hz, H-4); ^13^C NMR (CD_3_OD, 151 MHz) δ 18.5 (C-5’), 63.5 (C-5), 71.4 (C-4’), 72.9 (C-3’), 78.5 (C-6), 122.7 (C-3), 127.5 (C-1’), 135.7 (C-2’), 147.2 (C-4), 166.0 (C-2). ESI(+)-HRMS *m*/*z* 215.0911 [M + H]^+^ (calcd. for C_10_H_15_O_5_^+^, 215.0914, Δppm = 1.39). For NMR spectra, see Figures S6–S11.

5-Hydroxymethyl-furan-2-carboxylic acid (Sumikis’ acid) (3) (F8): ^1^H NMR (DMSO-*d*_6_, 600 MHz) δ 4.41 (2H, s, H-6), 5.12 (1H, s, OH-6), 6.31 (1H, d, *J* = 3.3 Hz, H-4), 6.84 (1H, d, *J* = 3.3 Hz, H-3); ^13^C NMR (DMSO-*d*_6_, 151 MHz) δ 55.8 (C-6), 107.9 (C-4), 115.0 (C-3), 148.2 (C-2), 157.4 (C-5). ESI(+)-HRMS *m*/*z* 143.0338 [M + H]^+^ (calcd. for C_6_H_7_O_4_^+^, 143.0339, Δppm = 0.59). For NMR spectra, see Figures S12–S17 [45].

(*Z*)-3-((2*R*,3*R*,6*R*)-3-Hydroxy-6-((*R*)-1-hydroxyethyl)-3,6-dihydro-2H-pyran-2-yl)acrylamide (4) (F11): ^1^H NMR (CD_3_OD, 600 MHz) δ 1.19 (3H, d, *J* = 6.4 Hz, H_3_-8), 3.71 (1H, p, *J* = 6.4, 5.8 Hz, H-7), 4.04 (1H, dq, *J* = 5.8, 2.0 Hz, H-2), 4.17 (1H, dd, *J* = 5.9, 2.8 Hz, H-6), 4.74 (1H, dt, *J* = 5.1, 2.8, 2.0 Hz, H-5), 6.10 (1H, ddd, *J* = 10.4, 5.1, 2.3 Hz, H-4), 6.18 (1H, d, *J* = 9.7 Hz, H-2’), 6.32 (1H, dt, *J* = 10.4, 1.2 Hz, H-3), 7.00 (1H, dd, *J* = 9.7, 5.9 Hz, H-1’); ^13^C NMR (CD_3_OD, 151 MHz) δ 18.8 (CH_3_-8), 66.2 (C-6), 69.9 (C-7), 71.5 (C-5), 80.6 (C-2), 123.3 (C-4), 124.8 (C-2’), 135.0 (C-3), 142.7 (C-1’), 165.4 (C-3’). ESI(+)-HRMS *m*/*z* 214.1071 [M + H]^+^ (calcd. for C_10_H_16_NO_4_^+^, 214.1074, Δppm = 1.40). For NMR spectra, see Figures S18–S23 [46].

Diplobifuranylone B (5) (F12): ${\left[a\right]}_{D}^{25}$ -44.4 (c. 0.11, MeOH); UV(MeOH) λ_max_ 206 (3.5), sh 285 (2.3) nm. ^1^H NMR (CD_3_OD, 600 MHz) δ 1.15 (2H, d, *J* = 6.4 Hz, H_3_-7’), 2.21 (1H, ddt, *J* = 12.8, 10.1, 6.4, 5.4 Hz, H-3b), 2.31 (1H, dddd, *J* = 12.8, 10.3, 8.0, 6.9 Hz, H-3a), 2.49 (1H, ddd, *J* = 17.4, 10.3, 6.4 Hz, H-4b), 2.62 (1H, ddd, *J* = 17.4, 10.1, 6.9 Hz, H-4a), 3.71 (1H, qd, *J* = 6.4, 4.8 Hz, H-6’), 3.81 (1H, s, OMe), 3.86 (1H, s, H-4), 4.58 (1H, ddd, *J* = 8.0, 5.4, 3.0 Hz, H-2), 4.63 (1H, tt, *J* = 4.8, 2.2, 1.6 Hz, H-5’), 4.93 (1H, t, *J* = 3.0, 1.9 Hz, H-2’), 5.92 (1H, dt, *J* = 6.4, 1.9 Hz, H-3’), 6.05 (1H, dt, *J* = 6.4, 2.2, 1.5 Hz, H-4’); ^13^C NMR (CD_3_OD, 151 MHz) δ 18.6 (C-7’), 24.8 (C-3), 29.0 (C-4), 53.1, 70.8 (C-6’), 82.5 (C-2), 89.6 (C-2’), 92.5 (C-5’), 128.2 (C-3’), 130.6 (C-4’), 180.5 (C-5). ESI(+)-HRMS *m*/*z* 199.0964 [M + H]^+^ (calcd. for C_10_H_15_O_4_^+^, 199.0965, Δppm = 0.51). For NMR spectra, see Figures S24–S34 [47].

3ξ-(1ξ-Hydroxyethyl)-7-hydroxy-1-isobenzofuranone (6) (F14): 6a: ^1^H NMR (CD_3_OD, 600 MHz) δ 1.26 (3H, d, *J* = 6.5 Hz, H_3_-2’), 4.20 (1H, qd, *J* = 6.5, 3.1 Hz, H-1’), 5.36 (1H, d, *J* = 3.1 Hz, H-3), 6.88 (1H, d, *J* = 7.4 Hz, H-6), 7.05 (1H, d, *J* = 7.4 Hz, H-4), 7.54 (1H, t, *J* = 7.4 Hz, H-5); ^13^C NMR (CD_3_OD, 151 MHz) δ 18.9 (C-2’), 68.7 (C-1’), 85.2 (C-3), 113.6 (C-8), 114.7 (C-4), 116.7 (C-6), 137.4 (C-5), 151.0 (C-9), 158.1 (C-7), 172.0 (C-1). 6b: ^1^H NMR (CD_3_OD, 600 MHz) δ 1.18 (3H, d, *J* = 6.5 Hz, H_3_-2’), 4.05 (1H, qd, *J* = 6.5, 4.5 Hz, H-1’), 5.34 (1H, d, *J* = 4.5 Hz, H-3), 6.90 (1H, d, *J* = 7.5 Hz, H-6), 7.07 (1H, d, *J* = 7.5 Hz, H-4), 7.54 (1H, t, *J* = 7.4 Hz, H-5); ^13^C NMR (CD_3_OD, 151 MHz) δ 18.0 (C-2’), 69.7 (C-1’), 85.7 (C-3), 113.1 (C-8), 115.0 (C-4), 116.9 (C-6), 137.4 (C-5), 150.6 (C-9), 158.3 (C-7), 171.9 (C-1). ESI(+)-HRMS *m*/*z* 195.0652 [M + H]^+^ (calcd. for C_10_H_11_O_4_^+^, 195.0652, Δppm = 0.15). For NMR spectra, see Figures S35–S40.

(2*Z*,4*Z*,8*E*)-6,7-Dihydroxydeca-2,4,8-trienoic acid (7) (F19): ^1^H NMR (CD_3_OD, 600 MHz) δ 1.67 (3H, ddd, *J* = 6.5, 1.7, 0.8 Hz, H_3_-10), 3.91 (1H, t, *J* = 6.9 Hz, H-7), 4.45 (1H, dd, *J* = 9.0, 6.9 Hz, H-6), 5.45 (1H, ddq, *J* = 15.3, 6.9, 1.7 Hz, H-8), 5.73 (3H, m, H-2, H-5, H-9), 7.00 (1H, t, *J* = 11.3 Hz, H-3), 7.32 (1H, t, *J* = 11.8 Hz, H-4); ^13^C NMR (CD_3_OD, 151 MHz) δ 18.0 (C-10), 71.5 (C-6), 76.9 (C-7), 121.2 (C-2), 127.5 (C-4), 129.5 (C-9), 131.2 (C-8), 138.8 (C-5), 139.3 (C-3), 170.2 (C-1). ESI(+)-HRMS *m*/*z* 199.0964 [M + H]^+^ (calcd. for C_10_H_15_O_4_^+^, 199.0965, Δppm = 0.51). For NMR spectra, see Figures S41–S46 [46].

(+)-(3*R*,4*S*)-4,5-Dihydroxymellein (8) (F17): ^1^H NMR (CD_3_OD, 600 MHz) δ 1.28 (3H, d, *J* = 6.9 Hz, H_3_-11), 4.85 (1H, overlapped, H-3), 4.94 (1H, d, *J* = 2.5 Hz, H-4), 6.85 (1H, d, *J* = 9.0 Hz, H-7), 7.11 (1H, d, *J* = 9.0 Hz, H-6); ^13^C NMR (CD_3_OD, 151 MHz) δ 18.1 (CH_3_-11), 64.4 (C-4), 82.7 (C-3), 108.2 (C-9), 119.1 (C-7), 124.2 (C-10), 126.2 (C-6), 148.8 (C-5), 156.4 (C-8), 169.8 (C-1). ESI(+)-HRMS *m*/*z* 211.0601 [M + H]^+^ (calcd. for C_10_H_11_O_5_^+^, 211.0601, Δppm = 0.10).). For NMR spectra, see Figures S46–S51 [48].

The raw NMR data files of the isolated compounds are available at the following Yareta link: https://doi.org/10.26037/yareta:5twbcdxdvbchnorxffaz7j4hoq (accessed on 19 August 2021).

### 2.11. ECD Experimental Details

The absolute configuration of the compounds was assigned according to the comparison of the calculated and experimental ECDs. After NMR analyses, the structure and the relative configuration proposed were used to perform a conformational random search using MMFF94s force field by Spartan Student v7 (Wavefunction, Irvine, CA, USA, 2019). Then, the 10 conformers with the lowest energy were subjected to PM3 and B3LYP/6-31G(d,p) geometrical optimizations in Gaussian16 software using CPCM model in methanol. The optimized conformers in each step were checked to avoid imaginary frequencies. Using a cut-off of 4 kcal/mol, the conformers were selected and submitted to Gaussian16 software for ECD calculations, using B3LYP/def2svp as basis set with CPCM model in methanol. The computation in Gaussian was performed at the University of Geneva on the Baobab cluster (https://plone.unige.ch/distic/pub/hpc/baobab_en, accessed on 19 August 2021). The calculated ECD spectrum was generated in SpecDis1.71 software (Version 1.71, T. Bruhn, Berlin, Germany, 2017) based in Boltzmann weighing average.

### 2.12. UHPLC-PDA-CAD-HRMS/MS Analysis

Metabolite profiling of the extracts was performed by UHPLC-PDA-CAD-HRMS/MS (Thermo Scientific, Bremen, Germany) as follows. The separation was achieved on an Acquity BEH C18 column (2.1 × 50 mm; 1.7 μm) (Waters, Milford, MA, USA). The temperatures in the autosampler and in the column oven were, respectively, set at 10 and 40 °C. The mobile phase was constituted of water (A) and acetonitrile (B) both containing 0.1% formic acid; the separation was performed with a linear gradient from 5 to 100% of B in 7 min followed by a 1 min isocratic step at 100% of B. The flow rate was set at 600 μL/min and the injection volume was set at 2 μL. The optimized HESI-II parameters were as follows: source voltage, 3.5 kV (pos), 4 kV (neg); sheath gas flow rate (N_2_), 55 units; auxiliary gas flow rate, 15 units; spare gas flow rate, 3.0; capillary temperature, 275 °C (pos), 320 °C (neg); S-Lens RF Level, 45. The data-dependent MS/MS events were performed on the four most intense ions detected in full scan MS (Top 3 experiment). The MS/MS isolation window width was 1 Da, and the normalized collision energy was set to 35 units. In data-dependent MS/MS experiments, full scans were acquired at a resolution of 35000 FWHM (at 200 *m*/*z*) and MS/MS scans at 17500 FWHM both with a maximum injection time of 50 ms. After being acquired in an MS/MS scan, parent ions were placed in a dynamic exclusion list for 2.0. In positive mode, the di-isoctyl phtalate C_24_H_38_O_4_ [M + H]^+^ ion (*m*/*z* 391.28429) was used as an internal lock mass. The mass analyzer was calibrated using a mixture of caffeine, methionine-arginine-alanine-acetate (MRFA), sodium dodecyl sulfate, sodium taurocholate and Ultramark 1621 in an acetonitrile/methanol/water solution containing 1% formic acid by direct injection. An Acquity UPLC photodiode array detector) (Waters, Milford, MA, USA) was used to acquire UV spectra which were detected in the 200–500 nm range.

### 2.13. UHPLC-HRMS/MS Data Processing

The raw files containing UHPLC-HRMS/MS data were converted into mzXML files using the MS convert software. The mzXML files were then processed using MZmine (v2.51, T. Pluskal, Prague, Czech Republic, 2019) (84).

For A02 extract analysis: Mass detection was carried out using a centroid mass detector with a noise level set at 5E^4^. The ADAP chromatogram builder was employed with a minimum height of 5E^4^, a minimum group size of scans of 5, a minimum group intensity threshold of 5E^4^, a minimum highest intensity of 5E^4^ and an *m*/*z* tolerance of 8 ppm. The wavelets ADAP algorithm was used for chromatogram deconvolution with the following settings: a single to noise (S/N) threshold of 10, intensity window SN, a minimum feature height of 5E^4^, a coefficient area threshold of 100, a peak duration range between 0.02 and 0.9 min and a wavelet range between 0.00 to 0.05 min. The *m*/*z* and retention time (RT) range for MS^2^ scan pairing were, respectively, set to 0.025 Da and 0.1 min. The chromatograms were deisotoped using the isotope peak grouper algorithm with an *m*/*z* tolerance of 8 ppm, an RT tolerance of 0.08 min and a maximum charge of 2, while the representative isotope used was the most intense. The peak alignment was performed using the join aligner method with an *m*/*z* tolerance of 8 ppm, an RT absolute tolerance of 0.05 min and a weight for *m*/*z* and RT at 20. An adduct search (Na^+^, K^+^, NH_4_^+^, ACN^+^, HCOOH) was performed on the peak list with an RT tolerance of 0.01 min, an *m*/*z* tolerance of 8 ppm, and a maximum relative peak height of 1000%. A complex search was performed on the peak list with an RT tolerance of 0.01 min, an *m*/*z* tolerance of 8 ppm, and a maximum relative peak height of 1000%. The peak list was then gap-filled using the same RT and *m*/*z* range gap filler with an *m*/*z* tolerance of 8 ppm. A custom database of the fungi group (17,255 compounds) was applied for in silico identification from the Dictionary of Natural Product (DVD version 26.2) (85). The full MS data set has been uploaded and is accessible on the GNPS servers as Massive Data set n°MSV000087760 (https://massive.ucsd.edu, accessed on 5 July 2021).

For pseudomonas supernatant analysis: Each experiment was performed in triplicate and all the samples were profiled in random order, with QCs included at regular times. 

Mass detection was carried out using a centroid mass detector with a noise level set at 1E^5^. The ADAP chromatogram builder was employed with a minimum height of 1E^5^, a minimum group size of scans of 5, a minimum group intensity threshold of 1E^5^, a minimum highest intensity of 1E^5^ and an *m*/*z* tolerance of 8 ppm. The wavelets ADAP algorithm was used for chromatogram deconvolution with the following settings: a single to noise (S/N) threshold of 100, intensity window SN, a minimum feature height of 1E^5^, a coefficient area threshold of 100, a peak duration range between 0.02 and 0.8 min and a wavelet range between 0.01 and 0.06 min. The *m*/*z* and retention time (RT) range for MS^2^ scan pairing were, respectively, set to 0.03 Da and 0.1 min. The chromatograms were deisotoped using the isotope peak grouper algorithm with an *m*/*z* tolerance of 6 ppm, an RT tolerance of 0.02 min and a maximum charge of 2, while the representative isotope used was the most intense. The peak alignment was performed using the join aligner method with an *m*/*z* tolerance of 8 ppm, an RT absolute tolerance of 0.03 min and a weight for *m*/*z* and RT at 10, and a spectra similarity comparison of 8 ppm with a cosine threshold of 0.75. An adduct search (Na^+^, K^+^, NH_4_^+^, ACN^+^, HCOOH) was performed on the peak list with an RT tolerance of 0.01 min, an *m*/*z* tolerance of 8 ppm, and a maximum relative peak height of 1000%. A complex search was performed on the peak list with an RT tolerance of 0.01 min, an *m*/*z* tolerance of 8 ppm, and a maximum relative peak height of 1000%. The peak list was then gap-filled using the peak finder filler with an *m*/*z* tolerance of 6 ppm*,* RT tolerance of 0.01 min and an intensity tolerance of 15%. The full MS data set has been uploaded and is accessible on the GNPS servers as Massive Data set n°MSV000087761 (https://massive.ucsd.edu, accessed on 5 July 2021).

### 2.14. Molecular Network Analysis

The MZmine files were exported in MGF format for the processing of the molecular network (MN) in the Global Natural Products Social (GNPS) platform [49]. In order to maintain the RT and exact mass information, and allow the isomer separation, feature-based MN were created using the MGF file resulting from the MZmine pre-treatment steps detailed above. The spectral data were then uploaded on the GNPS MN platform. A network was created where edges were filtered to have a cosine score above 0.65 and more than 6 matched peaks. Further edges between two nodes were kept in the network if, and only if, each of the nodes appeared in each other’s respective top 10 most similar nodes. The spectra in the network were then searched against GNPS’ spectral libraries. All matches kept between network spectra and library spectra were required to have a score above 0.7 and at least 6 matched peaks. The analogs spectra were searched against the DNP-ISDB database [50]. A top 50 of 2D chemical structures was provided for each node according to the in silico MS/MS fragmentation spectra match and was called “initial rank”. A chemo-taxonomical filter was applied to weight the search according to the occurrence of structures in Botryiosphaeriaceae to give a ranking of the top 6 most probable structures and was called “final rank” [51]. The output was visualized using Cytoscape v3.7.1 (v3.7.1, Pratt, Ono and Otasek, UCSD, San Diego, CA, USA, 2019). Peak areas of different analyses were represented as pie chart diagrams. Node sizes were proportional to the peak areas of the extract.

### 2.15. Antibacterial Assays

*Pseudomonas aeruginosa* (ATCC 27853) was used for the antibacterial assay. The MIC of the different compounds was determined in triplicate using the broth dilution method in 96-well microtiter plates [52]. Briefly, compounds were resuspended at 10.24 mg/mL in DMSO and serially diluted in Mueller−Hinton broth (MHB, Oxoid). The maximum initial concentration used for this assay was 256 μg/mL. After an incubation of 24 h at 37 °C, iodonitrotetrazolium chloride (INT, Sigma-Aldrich, St Louis, MO, USA) was added to each well, as growth indicator, and incubated for 1 h [53]. The highest dilution of a compound in which no growth appeared corresponded to its MIC. Gentamicin (INT, Sigma-Aldrich, St Louis, MO, USA) was used as control of inhibition and compared to the reference values.

### 2.16. Fluorescence Screening

The *pqsA::gfp* plasmid was constructed from the *lasB::gfp* plasmid [54] as follows. The *pqsA* promoter was amplified by PCR using GCTCTAGATCGAGCAAGGGTTGTAACGGTTTTTG and GCTGCTGCATGCGACAGAACGTTCCCTCTTCAGCGA primers and cloned by usual molecular methods into XbaI-SphI sites of *lasB::gfp* plasmid. Plasmids were transformed into *P. aeruginosa* PAO1 by electroporation. Reporter strains were grown at starting OD = 0.05 in PTSB (5% peptone, 0.25% trypticase soy broth) supplied with gentamycin 50 µg/mL and each sample at 128 µg/mL. Azithromycin 2 µg/mL was used as a positive control. Plates (black 96 well, Costar) were incubated at 37 °C, 160 rpm. After 15 h, OD600 and fluorescence at 480/520 nm were measured using microplate reader (SynergyHT BioTek, Winooski, VT, USA). Results are represented in percentage of fluorescence compared to the solvent control (DMSO) fixed at 100%.

### 2.17. qRT-PCR

Cultures of PAO1 were grown in triplicate during 4 h in presence of the compound of interest at 128 µg/mL. Then, bacteria were treated with RNAprotect Bacteria Reagent (Qiagen, Hilden, Germany) before centrifugation and storage at −20 °C. Pellets were resuspended in 100 µL TE pH8, lysozyme 1 mg/mL and incubated 5 min at RT. RNA was extracted using RNeasy kit (Qiagen, Hilden, Germany) according to manufacturer’s protocol. RNA was eluted in 40 µL of RNase-free water, quantified using Qubit 2.0 fluorometer (Invitrogen, Waltham, MA, USA) and DNase treated with RQ1 RNase-free DNase (Promega, Madison, WI, USA) according to manufacturer’s instructions. Then, 500 ng of RNA were reverse transcribed into cDNA using random primers (Promega, Madison, WI, USA) and Improm-II reverse transcriptase (Promega, Madison, WI, USA) according to the protocol. qPCR was performed using SYBR select master mix (Thermo Fisher, Waltham, MA, USA) with primers listed in Table S7 and *oprF* was used for normalization.

### 2.18. Rhamnolipids Assay

Rhamnolipids production was evaluated using cetyltrimethylammonium bromide (CTAB) agar test according to [55]. One mL of M8 1X, glucose 0.2%, MgSO_4_ 2 mM, methylene blue 0.0005%, glutamate 0.1%, CTAB 0.02% and agar 1.6% supplemented with sample at 128 µg/mL was poured in triplicate into a 24-well plate (TPP 92024). After solidification, 4 µL of ON culture was spotted in the center of each well and incubated for 24 h at 37 °C.

### 2.19. P. aeruginosa Culture for Supernatant Analysis

In a clear 96-well plate, overnight cultures of PAO1 at starting OD = 0.05 were grown in triplicate in LB medium with compound at 128 µg/mL or DMSO 1.28% in a final volume of 200 µL per well. The plate was then incubated at 37 °C, 160 rpm in orbital shaker, during 3 h, 6 h or 15 h. After incubation, cultures were transferred in a V-shaped bottom microplate (Corning, NY, USA) and centrifuged 20 min at 4500 rpm. Each supernatant was filtered using a 0.22 µm filter (bgb-analytik, Alexandria, VA, USA).

## 3. Results

### 3.1. Biological Activities of Fungal Extracts

A collection of 15 strains isolated as cultivable endophytes from the leaves of *A. sciophilum* were cultured and extracted with ethyl acetate (EtOAc). The extracts were then partitioned with water and both polar (W) and apolar (Et) extracts were screened for bioactivity. The antibacterial activity of all the extracts was tested against *Pseudomonas aeruginosa* (ATCC 27853). None of these extracts inhibited the growth at the maximum concentration tested (128 µg/mL). We therefore tested their ability to inhibit quorum sensing (QS) activity in *P. aeruginosa* (Pqs and Las systems). To this aim, we adapted an assay based on two reporter strains designed to monitor fluorescence induced by the quorum sensing systems Pqs and Las in *P. aeruginosa* [54]. Fluorescence was measured on the reporter strains grown with 128 µg/mL of extracts (EtOAc and water) and normalized to 100% of the reporter strain grown with 1.28% DMSO (negative control). Notable QS inhibition was highlighted for the water partition of the strain A02 (A02W), identified as *Lasiodiplodia venezuelensis,* which exhibited a clear specific inhibitory activity of 63% ± 0.4% for the Pqs and 49% ± 0.6% for the Las QS system. The positive control Azithromycin inhibited, respectively, 75 ± 5.3% and 80 ± 3.3% of the fluorescence induced by the Pqs and Las systems (Table S1). Based on those bioactivity results, the A02 strain was cultivated at a large scale (100 large Petri dishes) and extracted with the same procedure as for the screening. The large-scale water partition A02W was then tested in an independent series of measurements and confirmed the inhibitory activity observed in small scale, where it inhibited 52 ± 1.2% of the fluorescence of the pqs system and 28 ± 5.4% on the Las system, and had no effect on the growth (MIC > 128 µg/mL) (Table S1).

### 3.2. Bioactivity-Guided Fractionation and Isolation of the Bioactive Compounds

**Figure 1:**
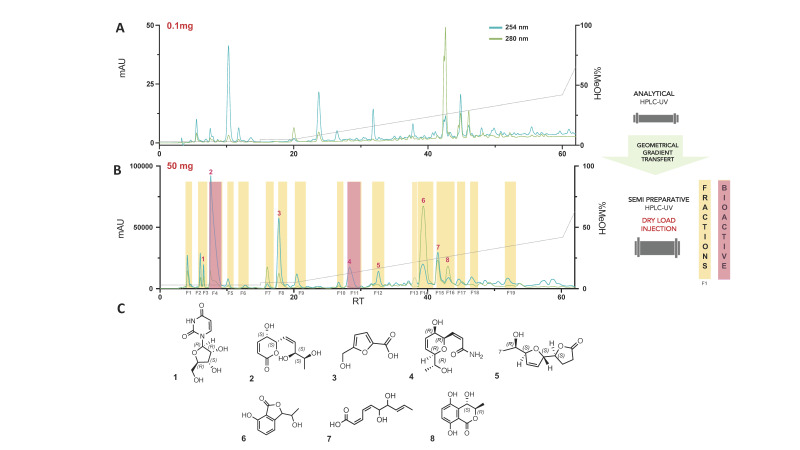
(**A**) Analytical HPLC-PDA profile of the water partition of *L. venezuelensis* extract (A02W). (**B**) Semi-preparative HPLC-UV profile of A02W with fractions collected by semi-preparative fractionation highlighted in yellow. The active fractions are displayed in pink. (**C**) Pure compounds isolated in a single step (UV peak-based fractionation) and identified via HRMS and NMR analyses.

In order to identify the individual fungal metabolites responsible for the QS inhibition activity, a careful fractionation of A02W was needed. For an efficient isolation of the active compounds, the reverse phase HPLC-PDA-ELSD conditions were optimized to obtain the highest possible chromatographic resolution of all detected peaks (Figure 1A). Those conditions were transferred to semi-preparative HPLC using a geometric gradient transfer method to obtain similar chromatographic selectivity [43]. In order to avoid a loss of resolution, the extract was introduced by dry load, following a protocol recently developed in our laboratory [56]. The high-resolution HPLC semi-preparative fractionation of the extract provided a good baseline separation of most compounds detected by UV (Figure 1B). This efficient process yielded nineteen fractions containing mainly single HPLC peaks. Fractions in sufficient amount were directly tested for their antibacterial activity (determination of MIC) and for their ability to inhibit QS on *lasB::gfp* and the *pqsA::gfp* reporter strains. While no growth inhibition was observed at 128 µg/mL, two fractions, named F4 and F11 (Figure 1B), showed strong QS inhibition activity (Table S2). 

**Figure 2:**
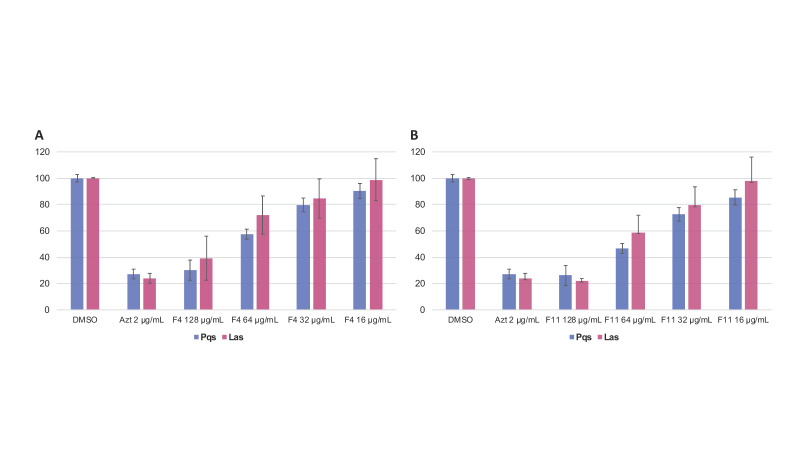
Dose-response effects of (**A**) fraction F4 and (**B**) fraction F11 on PAO1-*pqsA::gfp* (blue) and PAO1-*lasB::gfp* (red) reporter strains. All experiments were performed in a triplicate manner (technical and biological replicates), only representative data are shown. Azithromycin was used as a positive control. Fluorescence was normalized to 100% of the reporter strains grown with 1.28% DMSO (negative control).

F4 inhibited 70 ± 4.2% of the fluorescence induced by the *pqsA::gfp* strain and 64 ± 6.8% of the fluorescence induced by the *lasB::gfp* strain at 15 h. F11 inhibited 74 ± 2.8% of the fluorescence induced by the *pqsA::gfp* system and 77 ± 1.2% of the fluorescence induced by the *lasB::gfp* system at 15 h (Figure 2A,B). This QS inhibition followed a clear dose-response effect from 128 to 16 µg/mL.

### 3.3. Chemical Characterisation of the Secondary Metabolites Isolated from the Water Extract (A02W)

NMR and HRMS analysis of the fractions revealed the presence of two pure compounds: compounds 2 (F4) and 4 (F11) (Figure 1C). Compound 4 was identified as (*Z*)-3-((2*R*,3*R*,6*R*)-3-hydroxy-6-((*R*)-1-hydroxyethyl)-3,6-dihydro-2H-pyran-2-yl)acrylamide, a compound identified in the A02Et fraction of the same extract, in the frame of a screening for anticancer activity [46]. Compound 2 is a new natural product whose complete de novo structure elucidation is described hereafter.

**Figure 3:**
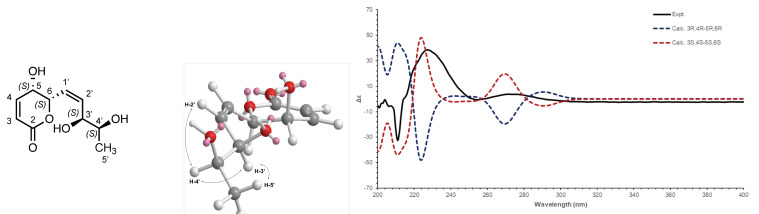
Structure and numbering of compound 2; ROESY correlations observed for the side chain; Experimental (black) and TFT-TD-calculated ECD spectra of the two enantiomers (5*R*,6*R*)-6-((3’*R*,4’*R*,Z) (blue) and (5*S*,6*S*)-6-((3’*S*,4’*S*,Z) (red) of 2.

Compound 2 (F4) was isolated as an amorphous solid. The ESI(+)-HRMS experiment indicated a molecular formula of C_10_H_14_O_5_ with an *m*/*z* of 215.0915 [M + H]^+^ (calcd. for C_10_H_14_O_5_^+^, 215.0914, Δppm = 0.40), which corresponded to 4 degrees of unsaturation. Analysis of the ^1^H-NMR and HSQC data of 2 indicated the presence of four ethylenic protons at δ_H_/δ_C_ 6.08/122.7 (H-3/C-3), 7.04/147.2 (H-4/C-4), 5.93/127.5 (H-1’/C-1’) and 5.85/135.7 (H-2’/C-2’); four oxymethines protons at δ_H_/δ_C_ 4.21/63.5 (H-5/C-5), 5.35/78.5 (H-6/C-6), 4.24/72.9 (H-3’/C-3’) and 3.74/71.4 (H-4’/C-4’), and a methyl group at δ_H_/δ_C_ 1.17/18.5 (H-5’/C-5’). The sequential COSY correlations from the methyl CH_3_-5’ to H-4’, H-3’, H-2’, H-1’, H-6, H-5, H-4 and H-3 and the HMBC correlation from H-3 and H-4 to the carbonyl ester at δC 166.0 indicated a 4,5,8,9-tetrahydroxydeca-2,6-dienoic acid or 4,5,8,9-tetrahydroxydeca-2,6-dienamide structure. To agree with the HRMS data, a cyclisation of the tetrahydroxydecadienoic acid structure had to take place and this was postulated between the acid function and the hydroxyl in C-6 to form a six-membered ring. Despite the lack of HMBC correlation between H-6 and C-2, the C-6 position was preferred because of the ^1^H and ^13^C deshielding of H/C-6. The ROESY correlation between H-5 and H-6 and the *J*_5,6_ value of 2.9 Hz positioned the propenamide side chain at C-6 in a pseudo-equatorial orientation and the hydroxy group at C-5 in axial orientation. Moreover, the positive Cotton effect in the circular dichroism curve (Δε 260–290 nm) was correlated with the C-6(*S*) configuration as described for substituted dihydro-α-pyrones in the literature [57]. The 11.4 Hz coupling constant for the set of olefinic protons H-1’ and H-2’ demonstrated the *cis* configuration of the double bond on the side chain. The small coupling constant between H-3’ and H-4’ (*J*_3′,4′_ = 4.7 Hz), the ROESY correlations from H-3’ to H-4’ and CH_3_-5’, and from H-4’ to H-2’ indicated a 3’*R*,4’*R* or 3’*S*,4’*S* configuration of the side chain (Figure 3). By comparison with the simulated ECD spectra (Figure 3), the absolute configuration of 2 was deduced as (5*S*,6*S*)-6-((3’*S*,4’*S*,*Z*)-3’,4’-dihydroxypent-1-en-1-yl)-5-hydroxy-5,6-dihydro-2H-pyran-2-one (Figures S6–S11).

Since the fractionation process yielded in one step additional pure products, their identification was carried out to gain more information on the composition of the polar partition extract of this strain. Six pure compounds (1, 3, 5–8) were then successfully isolated in a single step (Figure 1C) and identified by comparison with the literature as: the ubiquitous fungal metabolites uridine (1) (F3) [44] and 5-hydroxymethyl-furan-2-carboxylic acid (Sumikis’ acid) (3) (F8) [45], then, Diplobifuranylone B (5) (F12) [47], 3ξ-(1ξ-hydroxyethyl)-7-hydroxy-1-isobenzofuranone (6) (F14) [58], (2*Z*,4*Z*,8*E*)-6,7-dihydroxydeca-2,4,8-trienoic acid (7) (F19) [46] and (+)-(3*R*,4*S*)-4,5-dihydroxymellein (8) (F17) [59] (Figures S1–S51).

### 3.4. Untargeted Metabolomics to Search for Compounds 2 and 4 Analogues

The A02W extract, containing active compounds 2 and 4, was the only active extract in the screen. To understand the specificity and modulation of the activity with respect to the structure, a molecular network (MN) was generated to organize the tandem MS data acquired over the collection of endophytes. Molecular networking approaches allow us to organize untargeted tandem MS datasets based on their spectral similarity and, thus, to group analytes by structural similarity [49] and establish a comparison in the metabolome composition between the samples. Structural annotation in the MN was performed by comparison with an in-house database containing the in silico fragmentation spectra of all the compounds present in the dictionary of natural products DNP (DNP-ISDB) [50] followed by a taxonomically informed metabolite annotation process [51].

**Figure 4:**
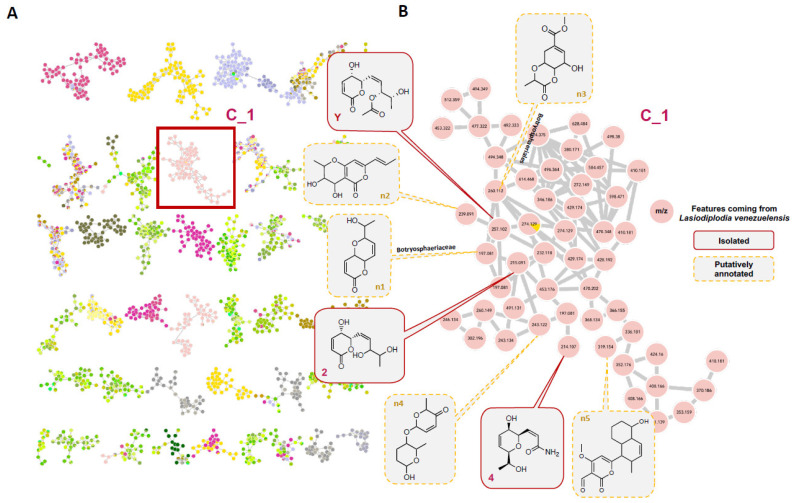
(**A**) Global molecular network of the 15 endophytic fungal extracts. Colors of the nodes correspond to the different strains present in the collection, with Cluster C_1 containing pyranone derivatives highlighted with a red square. (**B**) Cluster C_1, unique to the *Lasiodiplodia venezuelensis* strain, with annotated nodes (with the active compounds 2 and 4, and the previously isolated analog Y).

The active compounds 2 and 4 were localized and identified in the MN (Figure 4A), in a cluster containing pyran and pyranone derivatives (Figure 4B). This allowed verifying that they had potential structural analogues in this extract and five features could be putatively annotated (Figure 4B, Table S3). This cluster contained compounds only coming from the strain *L. venezuelensis* (W and Et extracts) and absent from other strains. Compound Y was present in the ethyl acetate extract and had been isolated from a previous screening [46]. Since this other *α,β*-unsaturated *δ*-lactone was available (compound Y, isolated and identified in [46]), it was tested but showed no anti-QS activity (Table S4). Y is an acetylated analogue of 2, and, based on the bioactivity results, this functionalization of the side chain might be responsible for the loss of activity. The presence of the other potential derivatives was checked in the other fractions, and those fractions were either inactive fractions or in too small quantity to be tested. 

### 3.5. Effect of Compounds 2 and 4 on the Expression of QS-Regulated Genes 

**Figure 5:**
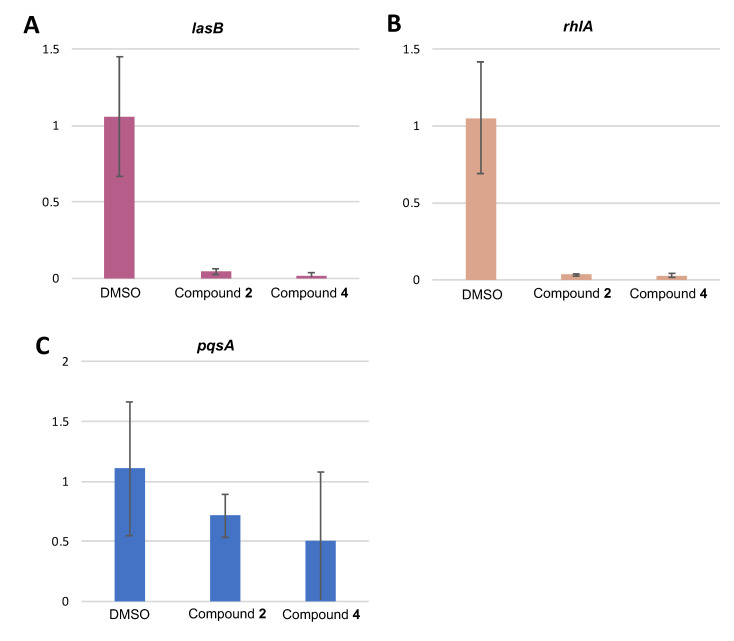
Effect of compounds 2 and 4 on the expression of QS-regulated genes: (**A**) *lasB*, (**B**) *rhlA*, (**C**) *pqsA.* Cultures of PAO1 were grown in triplicates with the compound of interest at 128 µg/mL for 4 h. DMSO 1.28% was used as solvent control. Error bars indicate standard deviations.

The influence of compounds 2 and 4 on the expression level of QS-regulated genes *lasB*, *pqsA* and *rhlA* in *P. aeruginosa* was thus observed using qRT-PCR on RNA extracted from PAO1 cultured with compounds 2 and 4 at 128 µg/mL, and DMSO (negative control) during 4 h. Compounds 2 and 4 almost totally suppressed the expression of the QS regulatory gene *lasB* controlled by the Las QS system (Figure 5A), and the *rhlA* gene, controlled by the Rhl QS system (Figure 5B). Surprisingly, although they showed an inhibition of *pqsA* signal during the screening, none of the compounds affected the expression of *pqsA* (Figure 5C). 

Intriguingly, no transcriptional inhibition was observed for the AI synthase genes *lasI* and *rhlI* nor for the transcriptional regulator genes *lasR*, *rhlR* and *MvfR* (Table S5). This suggests that the effect of inhibition mediated by 2 and 4 could act well downstream of the regulatory cascade, directly on the target genes of the QS.

### 3.6. Effect of the Compounds 2 and 4 on Virulence Factors Production

**Figure 6:**
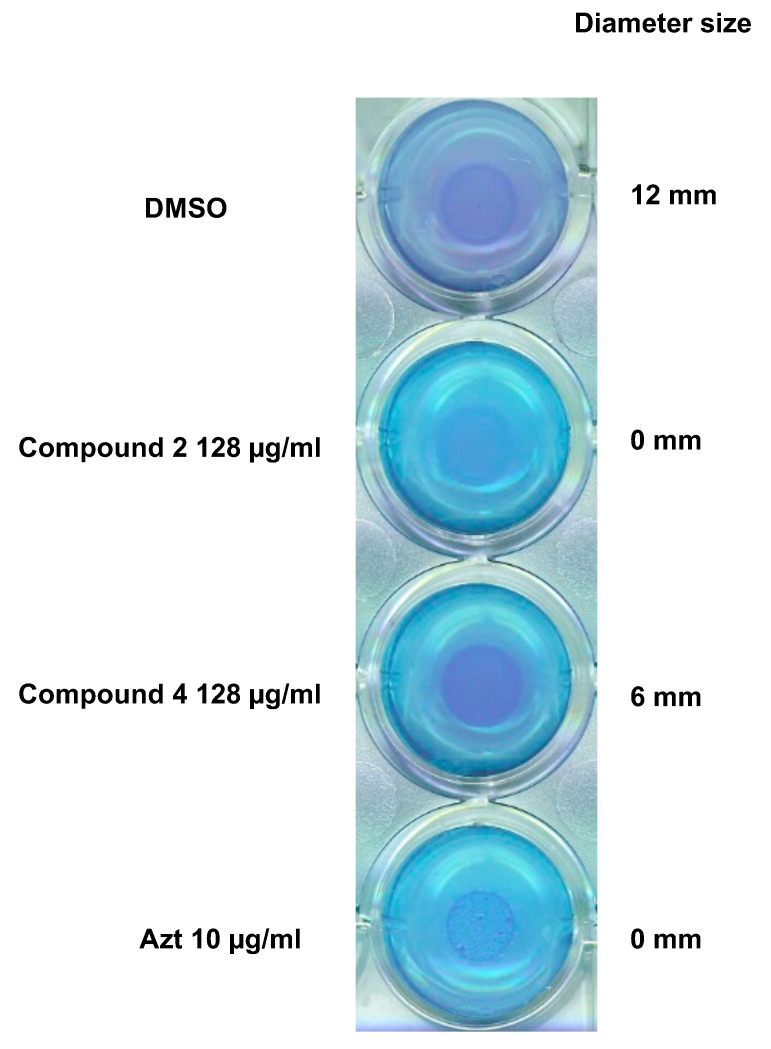
Effects of compounds 2 and 4 on rhamnolipids production. Compounds were tested in triplicate at a final concentration of 128 μg/mL.

Next, we evaluated the effect of the two compounds on the production of rhamnolipids, an important virulence factor involved in early cell-to-surface interactions and biofilm formation in *P. aeruginosa* [60]. The rhamnolipids production is indeed under QS regulation [61], they are therefore one of the indicators for the evaluation of the antivirulence activity of the compounds. The impact of compounds 2 and 4 on the release of rhamnolipids was evaluated by the cetyltrimethylammonium bromide (CTAB) agar test. In this particular medium, rhamnolipids precipitate with methylene blue forming a purple/blue halo around the bacterial colony [55]. PAO1 culture spotted on this medium showed that the rhamnolipids’ level was reduced by half when the medium was supplemented with compound 4 while no rhamnolipids at all were produced when supplemented with compound 2 at 128 µg/mL (Figure 6). These results show that both compounds are able to lower the production of this virulence factor, 2 being the more efficient.

### 3.7. Untargeted Metabolomics of P. aeruginosa PAO1 to Assess the Modulation of Virulence-Linked Metabolites When Incubated with 2 or 4

In order to obtain a more detailed view of the biological activity of 2 and 4, and, in particular, to observe their effect on the virulence signaling molecules involved in the QS of *P. aeruginosa*, a metabolomic study was undertaken. For this, a similar UHPLC HRMS/MS profiling approach as the one used for the analysis of the composition of the fungal strain A02 was applied. We recently used this strategy to identify and compare secreted bacterial compounds between wild type and mutants of *P. aeruginosa* [62]. Indeed, it has been demonstrated that about one third of the metabolome of *P. aeruginosa* was linked to QS, with secondary metabolite levels modulated by a lack of QS signaling molecules [63], and that clinical strains with different virulence phenotypes also had different metabolic profiles. A large part of these differences was associated with known virulence-linked metabolites such as rhamnolipids, alkyl quinolones, siderophores (pyochelin) and phenazines (pyocyanin) [64]. Recent advances in the development of open access MS databases, such as GNPS [49], and available data on the specialized metabolome of *P. aeruginosa*, allow for confident identification of these key groups of metabolites directly in the *P. aeruginosa* PAO1 supernatant [65].

To evaluate the effect of the active compounds 2 and 4 on these metabolites, supernatants of the reference *P. aeruginosa* strain PAO1 under different conditions (medium alone, PAO1, PAO1 incubated with 1.28% DMSO (negative control), PAO1 incubated with 2 or 4, PAO1 spiked with 2 or 4 after 15 h, in triplicate, repeated for different time points: 3 h (early exponential phase), 6 h (late exponential phase) and 15 h (stationary phase), were profiled by UHPLC-HRMS/MS. The data were organized as a molecular network (MN). All features were annotated by comparison with the DNP-ISDB followed by a taxonomically informed annotation process [51]. Spiked PAO1 with 2 and 4 was also used to demonstrate that the metabolome variations observed were due to the incubation with the tested molecules.

**Figure 7:**
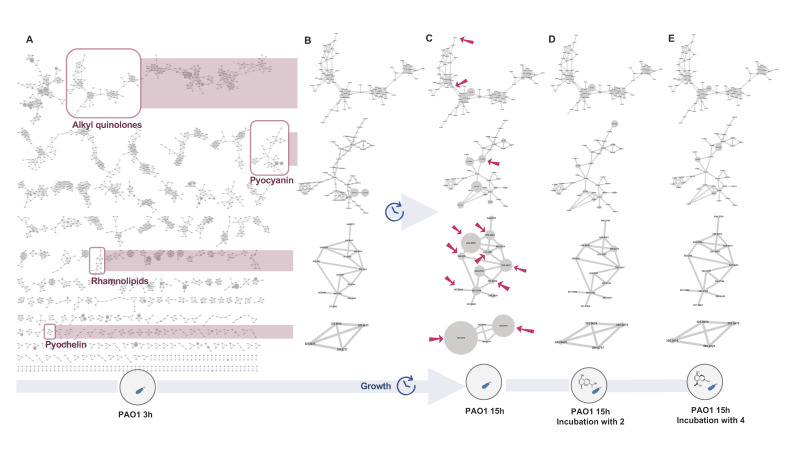
(**A**) Full molecular network of PAO1 alone after 3 h of incubation, with QS-related metabolites clusters highlighted with red squares. (**B**–**E**) Variation of the intensity of all features associated with virulence factors at 3 and 15 h, with or without incubation with the active compounds. (**B**) Clusters from PAO1 alone after 3 h. (**C**) Clusters from PAO1 alone after 15 h. (**D**) Clusters from PAO1 incubated with compound 2 after 15 h. (**E**) Clusters from PAO1 incubated with compound 4 after 15 h. Compounds were tested in triplicate at a final concentration of 128 μg/mL. The size of the nodes is proportional to the intensity of each feature in the samples. Metabolites significantly impacted by incubation with 2 or 4 are highlighted by red arrows.

In a first step, to globally assess the modulation of PAO1 metabolome, the intensity of each feature in the MN at 3 h versus 15 h (which corresponds to the production of QS metabolites) was compared. This showed that many features presented signals with significantly stronger intensities after 15 h of growth (Figure 7A–C).

Our annotation workflow and literature data permitted the identification of four clusters belonging to four families of specialized metabolites produced by *P. aeruginosa*: alkyl quinolones (AQs), rhamnolipids, pyocyanin and pyochelin (highlighted in red in Figure 7A and Figures S52–S57 and Table S6). The four clusters corresponding to QS molecules were monitored over time and when PAO1 was incubated with 2 or 4 (Figure 7B–E), the effects of the bioactive compounds 2 and 4 were shown by the decrease in the size of specific nodes in the highlighted clusters, which suggested a potential effect on the QS metabolites.

In a second step, a detailed differential analysis of the relative peak heights of the features in these clusters enabled monitoring of their production under the different conditions.

In the alkyl quinolone cluster, which shows many derivatives, some nodes were increased in size after 15 h of incubation. These nodes correspond to C9 HHQ (4-heptyl-4(*1H*)-quinolone) congeners, the C9 HHQdb, and the C7 and C9 HQNOs (2-heptyl-4-hydroxyquinoline-N-oxide) and their double-bonded congeners (see Figure 7C, Figures S52 and S53). These findings are consistent with the literature where more than 50 diverse HAQs have been identified, C7 and C9 congeners being the most abundant [66,67]. Comparison of this cluster with that recorded with incubation with 2 or 4, showed that no HAQs appeared to be significant except for the C4 HHQ and C6 HHQdb. These features, highlighted by red arrows in Figure 7C, show significant decrease in their intensities (Figure 7D,E). This is better highlighted in Figures S52 and S53.

The rhamnolipid cluster consisted of the rhamnose-free precursors: 3-(3-hydroxyalkanoyloxy) alkanoic acids (HAAs), rhamnolipids containing one (Rha) or two (Rha-Rha) rhamnoses [65]. All these forms of rhamnolipids were impacted by the incubation of 2 or 4 (Figure 7C–E and Figure S55).

For the rhamnolipids precursors HAAs, compound 2 induced an 8-fold and 9-fold decrease in the production of C10-C12 and C10-C10 HAAs, respectively. Compound 4 seemed to have a stronger effect with a 14-fold and 15-fold decrease in these HAAs, respectively. A decrease in the production of mono-rhamnolipids (C10-C10 and C10-C12) was also observed when PAO1 was incubated with 2 or 4, with a stronger effect exerted by compound 4. Likewise, the di-rhamnolipids were affected, notably with 4, with a total inhibition of the production of Rha-Rha C10-C10.

The pseudomonal iron siderophore pyochelin is known to exist in enantiomeric forms, cis and trans, and both could be annotated in the network (Figure 7C and Figure S56) [65]. Metabolomic data showed a 28-fold decrease in pyochelin relative intensity when incubated with 2 and 17-fold when incubated with 4. Similarly, the intensity of the other pyochelin enantiomer was reduced by 18-fold for 2 and 20-fold for 4 (Figure 7C–E and Figure S56).

Pyocyanin was identified in another cluster (red arrow in Figure 7C and Figure S57) among potential derivatives. A 3-fold decrease of its production was observed when PAO1 was incubated with either 2 or 4 (Figure 7C–E and Figure S57).

## 4. Discussion

Antivirulence therapy is a relevant strategy to overcome the antibiotic resistance crisis. QS-based approaches could help mitigate the development of microbial resistance by targeting the virulence of pathogenic bacteria rather than their growth, thereby reducing the selective pressure applied to these pathogens when confronted with antibiotics. Targeting QS in *P. aeruginosa* is suggested as a complementary strategy for the treatment of infections [68].

In this study, we hypothesized that the endophyte community living in the long-lived palm *A. sciophilum* could contribute to the protection and resistance of its leaves. It is not surprising that endophytes presenting quorum response to target virulence and pathogenicity occur in plant microbiome communities. They represent an integral part of plant systems and one of their functional traits includes inhibition of pathogen quorum molecules [26]. In our host–microbe interaction model, *L. venezuelensis* might produce the identified bioactive metabolites 2 and 4 to counter phytopathogens attacking *A. sciophilum* and maintain their symbiotic association and colonization in the internal tissues of the palm.

2 and 4 contain substituted pyrans and 2 notably contains a 5,6-dihydro-α-pyrone, also called α,β-unsaturated δ-lactone. This type of scaffold is frequently encountered in natural products and exhibits a wide range of biological activities [69]. The α,β-unsaturated δ-lactone system of such compounds was highlighted to play a role in the antimicrobial activity since they are well-known Michael acceptors [57]. Such compounds have been isolated from various fungal species, as, for instance, phomopsolides from a *Penicillium sp.,* endophyte of *Taxus brevifolia* and *Phomopsis oblonga* [70]. Phomopsolides A and B (derivatives of compound 2, esterified in position 5) have notably shown antimicrobial properties [71]. In the genus Lasiodiplodia, two 5,6-dihydro derivatives of α,β-unsaturated δ-lactones ((*R*)-2-Octeno-δ-lactone (lasiolactone) and tetrahydro-4-hydroxy-6-propylpyran-2-one (Massoia lactone)), have been isolated from *Lasiodiplodia theobromae* and a strain of *Lasiodiplodia* PSU-M114 [72]. They were to date the only representative of this family of compounds reported in *Lasiodiplodia*. 

In an interesting way, it has been shown that the ethyl acetate extract of a *Lasiodiplodia sp.* was able to inhibit violacein production in *Chromobacterium violaceum* CV026 without impairing its growth, leading to the thinking that this organism might contain QS inhibitory metabolites [73]. Another recent study demonstrated the anti-biofilm activity of the ethyl acetate extract of *L. pseudotheobromae* against the foodborne bacterium *Yersinia enterocolitica* [74]. However, to date, the compounds responsible for those activities have not been reported. The present work identifies 2 and 4 as responsible for the QSI activity exhibited by *L. venezuelensis* ethyl acetate extract. It is likely that these kind of molecules were also involved in the bioactivities reported for the other *Lasiodiplodia* species.

It is worth noting that some α-pyrones may act as communication molecules in *Pseudomonas* [75]. Pyrones derivatives such as 2 might have an antagonistic function by disrupting this pyrone-signal in bacteria, as in the case of AHL signaling [76].

2 and 4 showed strong inhibition of the *lasB::gfp* and the *pqsA::gfp* reporter strains at 128 µg/mL, in a dose-dependent manner. Both compounds almost completely inhibited the expression of the QS regulatory genes *lasB* and *rhlA*, but had surprisingly no effect on transcription for the AI synthase genes *lasI* and *rhlI* nor the transcriptional regulator genes *lasR*, *rhlR* and *mvfR.* This suggests that those two compounds could act downstream in the QS pathway by directly targeting the genes involved in the synthesis of virulence factors. Moreover, the fact that the growth of *P. aeruginosa* is not affected by these two compounds suggests that they specifically interfere with the QS regulators, without toxic effects on the bacteria. This makes 2 and 4 good potential QSIs since a QSI that does not disrupt life processes and growth minimizes selective pressure for resistance development.

The effect of 2 and 4 on the QS metabolites associated with the virulence of *P. aeruginosa* was evaluated through untargeted metabolomics of the *P. aeruginosa* supernatant*,* after incubation with the compounds. This showed that 2 and 4 induced a diminution of the production of the rhamnolipid precursors as well as the mono and di-rhamnolipids. This confirmed the observed effect on the RhlA system at the transcriptional level. Indeed, RhlA encoded by the *rhlA* gene is responsible for the synthesis of the precursor HAA. It is well established that the mono and di-rhamnolipids are formed by the subsequent assemblage between HAAs and dTDP-L-rhamnose by the RhlB rhamnosyltransferase, encoded by the *rhlB* gene [61]. The observed concomitant inhibition of the precursors and the rhamnolipids can thus be explained by the diminution of the HAAs precursors. We then confirmed the global decrease of the rhamnolipids production induced by 2 and 4 in vivo in a CTAB assay. We also observed a diminution of the pyocyanin production by 2 and 4. In several studies, loss in the production of pyocyanin was observed by deletion of *lasR*–*lasI*, *rhlR*–*rhlI* QS systems [77]. The loss of pyocyanin production observed here could, therefore, confirm the effect on the Rhl and Las systems. The siderophore pyochelin was also significantly inhibited when 2 and 4 were present. The production of siderophores such as pyochelin and pyoverdine is essential for the proliferation of *P. aeruginosa* in several types of infections. They are crucial to overcome the iron limitation in the host organism [78]. In burn wound infections, several studies have revealed a strong increase in the expression of genes involved in iron uptake, including pyoverdine production [79]. Gonzales et al. showed that this was accompanied by increased production of proteases and rhamnolipids via upregulation of *LasB* and *RhlA* [80].

By blocking pyoverdine production and interfering with the LasB and RhlA QS pathways, 2 and 4 could represent good candidates to impede pathogen development in certain situations such as *P. aeruginosa* infection in severely burned patients.

During the past 15 years, a significant range of QSIs have been discovered, with three main strategies to interfere with bacterial QS: suppression of the signal production, antagonism of the receptor and degradation of the AIs. QSIs include synthetic molecules as well as natural products (NP) and act as AHL-like or non-AHL-like antagonists, C-covalent binders and NP-based inhibitors [81]. Most of the Las and Rhl systems competitive inhibitors have been based on modifications of the native signaling molecules, either in the head group, or the acyl tail part [82]. The study of the crystal structure of the active sites and ligand-binding domains helped to model interactions between the diverse QSIs and the various targets involved, and to obtain insight into potential structure–activity relationships (SAR). Various studies have suggested that, for small molecules, a hydrophilic head group associated to a lipophilic acyl chain are key features for the modulation of QS activity [83,84]. Structurally, compounds 2 and 4 meet such criteria, and this could explain their effect on the QS targets. However, their alkyl chain is much shorter than the natural AHL and might lead to instability of the interactions, even though not having a highly lipophilic acid chain might be more favorable for pharmacokinetic properties. 

## 5. Conclusions

In our study, we showed that 2 and 4, two low-molecular-weight molecules, are able to act on multiple targets in *P. aeruginos*a QS systems [14]. They significantly reduce the expression of the QS-regulated genes *lasB* and *rhlA* without impairing the growth of *P. aeruginosa*, while also inhibiting the production of some critical virulence factors. We also give some insights on the structure–activity relationships between 2 and 4 and inactive analogues. These two small molecules represent an interesting pharmacophore starting model for the development of more effective *P. aeruginosa* QSIs.

## Data Availability

The datasets generated for this study are available on request to the corresponding author.

## Supplementary Materials

The following are available online at https://www.mdpi.com/article/10.3390/microorganisms9091807/s1.
