# Supplementary material for: Characterization of Pseudomonas aeruginosa Quorum Sensing Inhibitors from the Endophyte Lasiodiplodia venezuelensis and Evaluation of Their Antivirulence Effects by Metabolomics
Source: Microorganisms. 2021 Aug 25;9(9):1807. doi: 10.3390/microorganisms9091807 (PMC8465504; doi:10.3390/microorganisms9091807)
Supplement: Supplementary file 1 [file microorganisms-09-01807-s001.zip › A02W_Supplementary_material/Supplementary_ material.pdf]

Supplementary material

Table S1. Effects of the A02W extract (obtained in small scale and in large scale) on PAO1 *pqsA::gfp* and *lasB::gfp* reporter strain after 15h of growth. Azithromycin at 2 µg/mL was used as a positive control. DMSO 1.28 % was used as solvent control. Fluorescence was normalized to 100% of the reporter strain grown with DMSO 1.28%. All experiments were done in triplicate manner (technical and biological replicates).

	PqsA (% fluo)	SD	LasB (% fluo)	SD	CMI *P. aeruginosa* ATCC 27853
**A02W 128 µg/mL (Small scale)**	37	0.4	51	0.6	> 128 µg/mL
**A02W 128 µg/mL (Up scale)**	48	1.2	72	5.4	> 128 µg/mL
**Azt 2 µg/mL (C+)**	25	5.3	20	3.3	-

Table S2. Effects of the A02W fractions on PAO1 *pqsA::gfp* and *lasB::gfp* reporter strain after 15h of growth. Azithromycin at 2 µg/mL was used as a positive control, DMSO 1.28 % was used as solvent control. Fluorescence was normalized to 100% of the reporter strain grown with DMSO 1.28%. All experiments were done in triplicate manner (technical and biological replicates).

A02W Fractions (128 µg/mL)	PqsA (% fluo)	SD	LasB (% fluo)	SD	CMI *P. aeruginosa* ATCC 27853
**F1**	95	2.8	88	2.6	> 128
**F2**	93	0.7	78	1.5	> 128
**F3**	76	14.2	62	0.2	> 128
**F4**	**30**	**4.2**	**36**	**6.8**	**> 128**
**F5**	91	6.0	73	1.2	> 128
**F9**	86	4.3	75	2.2	> 128
**F11**	**26**	**2.8**	**23**	**1.2**	**> 128**
**F12**	101	3.8	74	1.4	> 128
**F13**	87	19.4	102	8.3	> 128
**F14**	100	6.0	84	6.4	> 128
**F18**	90	1.7	88	1.0	> 128
**F19**	89	1.3	72	1.1	> 128
**Azt 2 µg/mL (C+)**	25	5.3	20	3.3	> 128

Table S3. Annotation table of cluster C_1.

	RT (min)	m/z	Molecular formula	Molecule Name	Taxonomy							Classification			Inchi
		[M+H]+			Kingdom	Phylum	Class	Order	Family	Genus	Species	Class	SubClass	Parent level 1	
**Compound 2**	0.42	215.0913	$C_{10}H_{14}O_5$	(5S,6S)-6-((3'S,4'S,Z)-3',4'-dihydroxypent-1-en-1-yl)-5-hydroxy-5,6-dihydro-2H-pyran-2-one.	Fungi	Ascomycota	Dothideomycetes	Botryosphaeriales	Botryosphaeriaceae	Lasiodiplodia	Lasiodiplodia venezuelensis	Pyrans	Pyranones and derivatives	Butenolide	InChI=1S/C10H14O5/c1-6(11)7(12)2-4-9-8(13)3-5-10(14)15-9/h2-9,11-13H,1H3/b4-2-/t6?,7?,8-,9-/m0/s1
**Compound 4**	0.8	214.1072	$C_{10}H_{15}NO_4$	(Z)-3-((2R,3R,6R)-3-hydroxy-6-((R)-1-hydroxyethyl)-3,6-dihydro-2H-pyran-2-yl)acrylamide	Fungi	Ascomycota	Dothideomycetes	Botryosphaeriales	Botryosphaeriaceae	Lasiodiplodia	Lasiodiplodia venezuelensis	Pyrans	Pyranones and derivatives	Butenolide	InChI=1S/C10H15NO4/c1-6(12)8-3-2-7(13)9(15-8)4-5-10(11)14/h2-9,12-13H,1H3,(H2,11,14)/b5-4-/t6-,7+,8+,9+/m0/s1
**Node n1**	0.26	197.0807	$C_{10}H_{12}O_4$	Diplopyrone	Fungi	Ascomycota	Dothideomycetes	Botryosphaeriales	Botryosphaeriaceae	Diplodia	Diplodia mutila	Pyrans	Pyranones and derivatives	Butenolide	InChI=1S/C10H12O4/c1-6(11)7-2-3-9-8(13-7)4-5-10(12)14-9/h2-9,11H,1H3
**Node n2**	0.98	239.0910	$C_{12}H_{14}O_5$	Radicinol3-Epimer	Fungi	Ascomycota	Dothideomycetes	Pleosporales	Pleosporaceae	Alternaria	Alternaria radicina	Pyrans	Pyranones and derivatives	Butenolide	InChI=1S/C12H14O5/c1-3-4-7-5-8-9(12(15)17-7)11(14)10(13)6(2)16-8/h3-6,10-11,13-14H,1-2H3/b4-3+
**Node n3**	0.71	260.1123 [M+NH3]+ of 242.079	$C_{11}H_{14}O_6$	Scytolide3S,10-Dihydro	Fungi	Ascomycota	Dothideomycetes	Botryosphaeriales	-	-	-	Pyrans	Pyranones and derivatives	Butenolide	InChI=1S/C11H14O6/c1-5-10(13)17-9-7(12)3-6(11(14)15-2)4-8(9)16-5/h3,5,7-9,12H,4H2,1-2H3
**Node n4**	1.02	243.1222	$C_{12}H_{18}O_5$	Antibiotic SEN 366F	Fungi	Ascomycota	-	-	-	-	-	Pyrans	Pyranones and derivatives	Butenolide	InChI=1S/C12H18O5/c1-7-9(13)3-6-12(16-7)17-10-4-5-11(14)15-8(10)2/h3,6-8,10-12,14H,4-5H2,1-2H3
**Node n5**	2.36	319.1537	$C_{18}H_{22}O_5$	Solanapyrone B6Œ≤-Hydroxy, 1''-aldehyde	Fungi	Ascomycota	Dothideomycetes	Pleosporales	Pleosporaceae	Alternaria	tenuissima SP-07	Pyrans	Pyranones and derivatives	Butenolide	InChI=1S/C18H22O5/c1-10-6-7-11-12(4-3-5-14(11)20)17(10)16-8-15(22-2)13(9-19)18(21)23-16/h6-12,14,17,20H,3-5H2,1-2H3

Table S4. Effect of compound **Y** on PAO1 *pqsA::gfp* and *lasB::gfp* reporter strain after 15h of growth. Azithromycin at 2 µg/mL was used as a positive control. DMSO 1.28 % was used as solvent control. Fluorescence was normalized to 100% of the reporter strain grown with DMSO 1.28 %. All experiments were done in triplicate manner (technical and biological replicates).

	PqsA (% fluo)	SD	LasB (% fluo)	SD	CMI *P. aeruginosa* ATCC 27853
**Compound Y 128 µg/mL**	68	3.9	72	0.6	> 128 µg/mL
**Azt 2 µg/mL (C+)**	25	5.3	20	3.3	-

Table S5. Effect of compounds **2** and **4** on the expression on QS regulated genes: *lasI*, *lasR, rhlI*, *rhlR*, *MvfR,* expressed as relative expression. Cultures of PAO1 were grown in triplicate with the compound of interest at 128 µg/ml during 4 hours. DMSO 1.28 % was used as solvent control. Error bars indicate standard deviations.

	lasI	*SD*	*lasR*	*SD*	*rhlI*	*SD*	*rhlR*	*SD*	*pqsR*	*SD*
**Compound 2 128 µg/mL**	0.34	0.34	0.60	0.14	0.36	0.39	0.64	0.40	1.03	0.20
**Compound 4 128 µg/mL**	0.61	0.67	0.47	0.65	0.47	0.58	0.41	0.26	0.98	0.86
**DMSO**	1.05	0.36	1.08	0.49	1.05	0.34	1.03	0.27	1.06	0.40

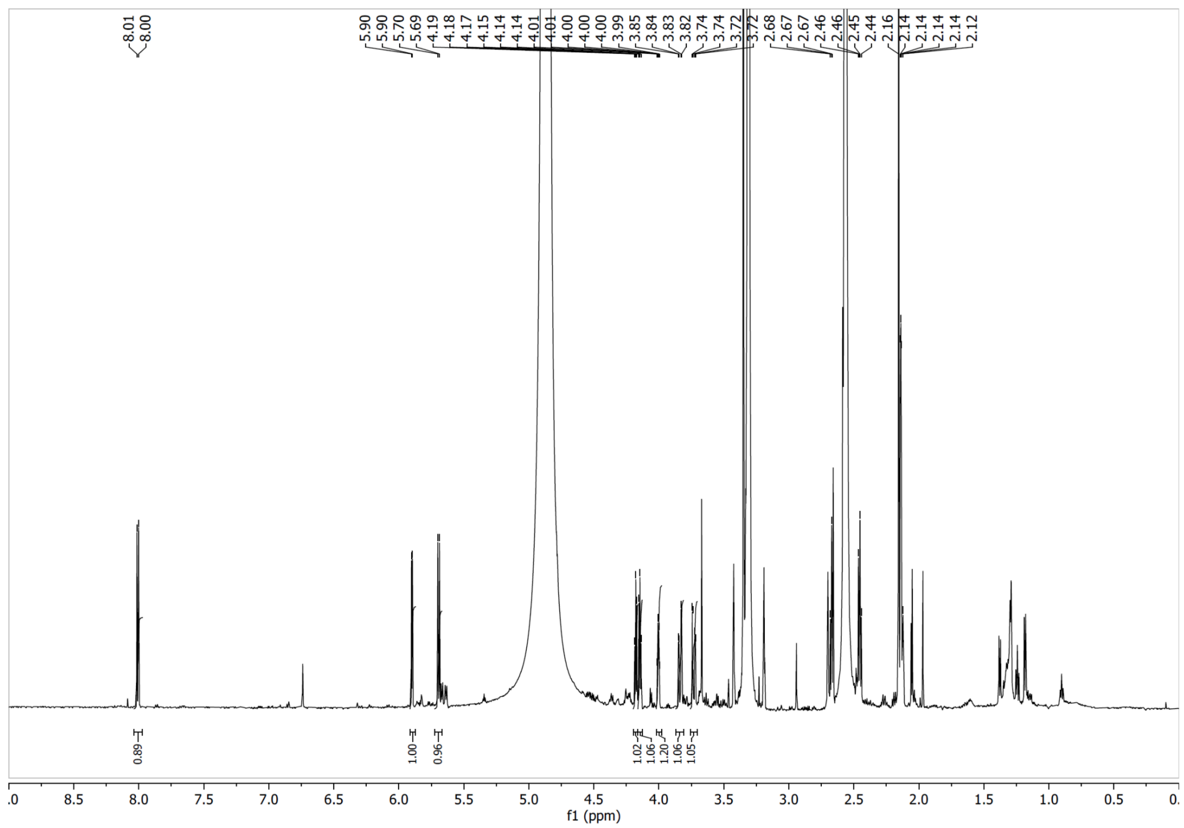

Figure S1. ^{1}H NMR spectrum of compound **1** in CD_3OD at 600 MHz

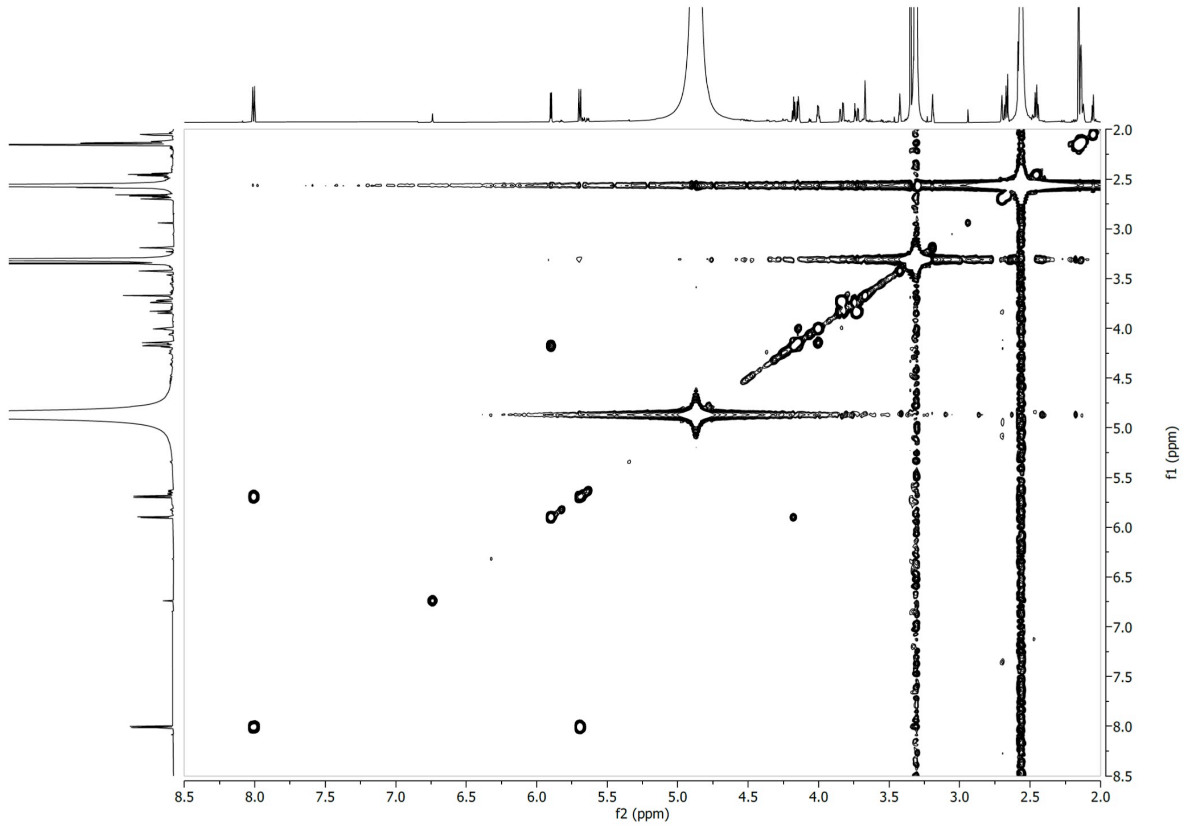

Figure S2. COSY NMR spectrum of compound **1** in CD_3OD

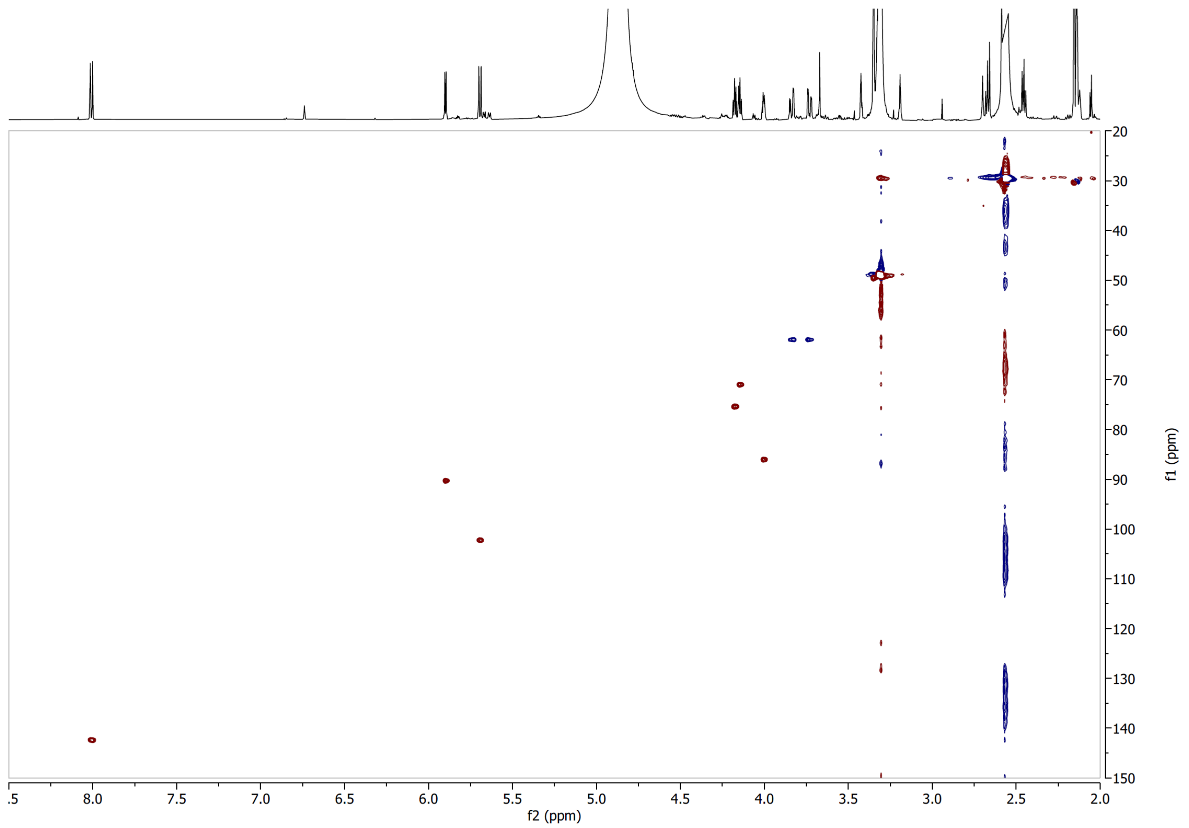

Figure S3. Edited-HSQC NMR spectrum of compound **1** in CD_3OD

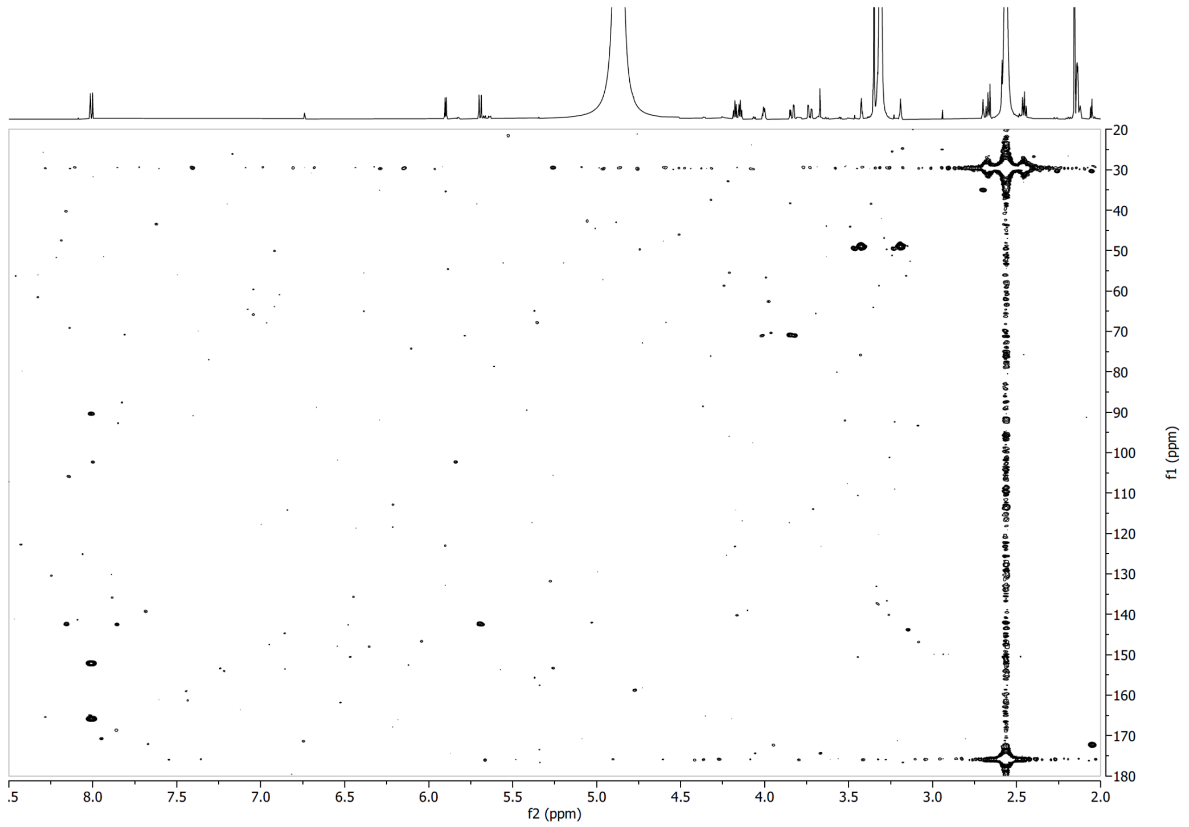

Figure S4. HMBC NMR spectrum of compound **1** in CD_3OD

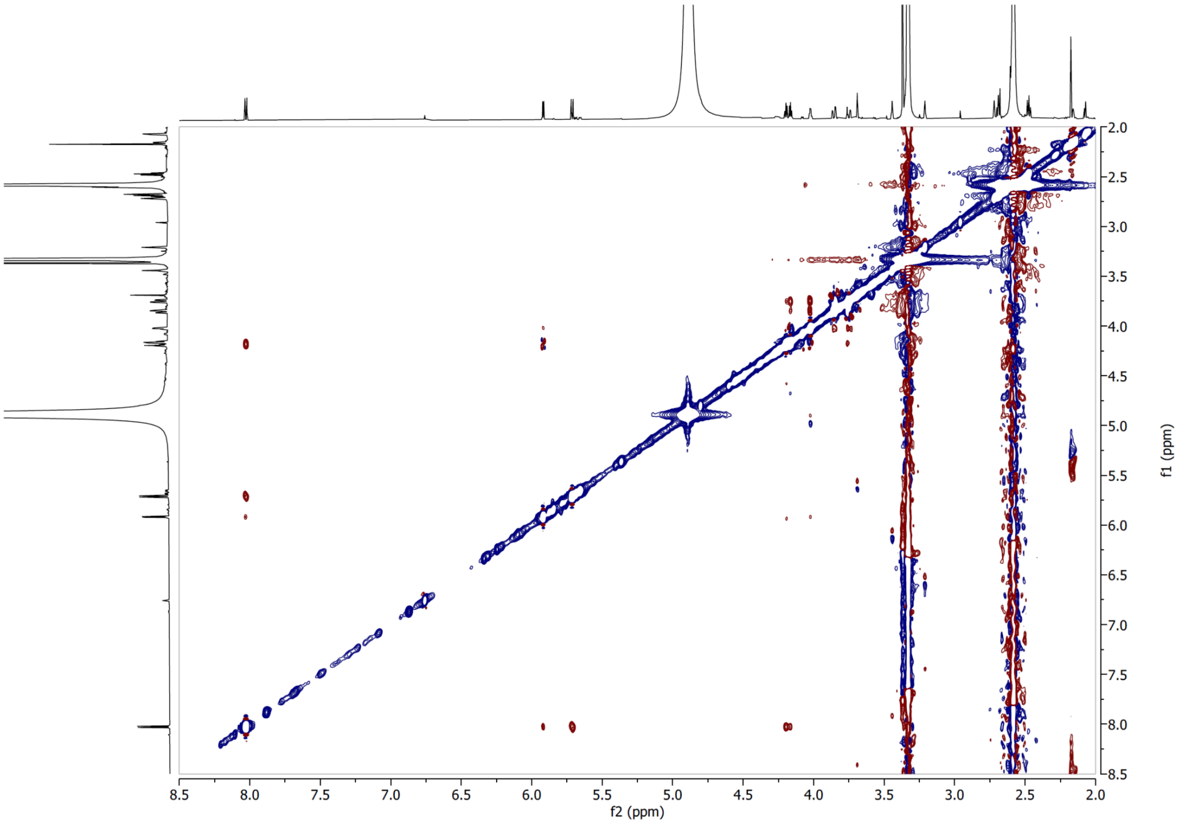

Figure S5. ROESY NMR spectrum of compound **1** in CD_3OD

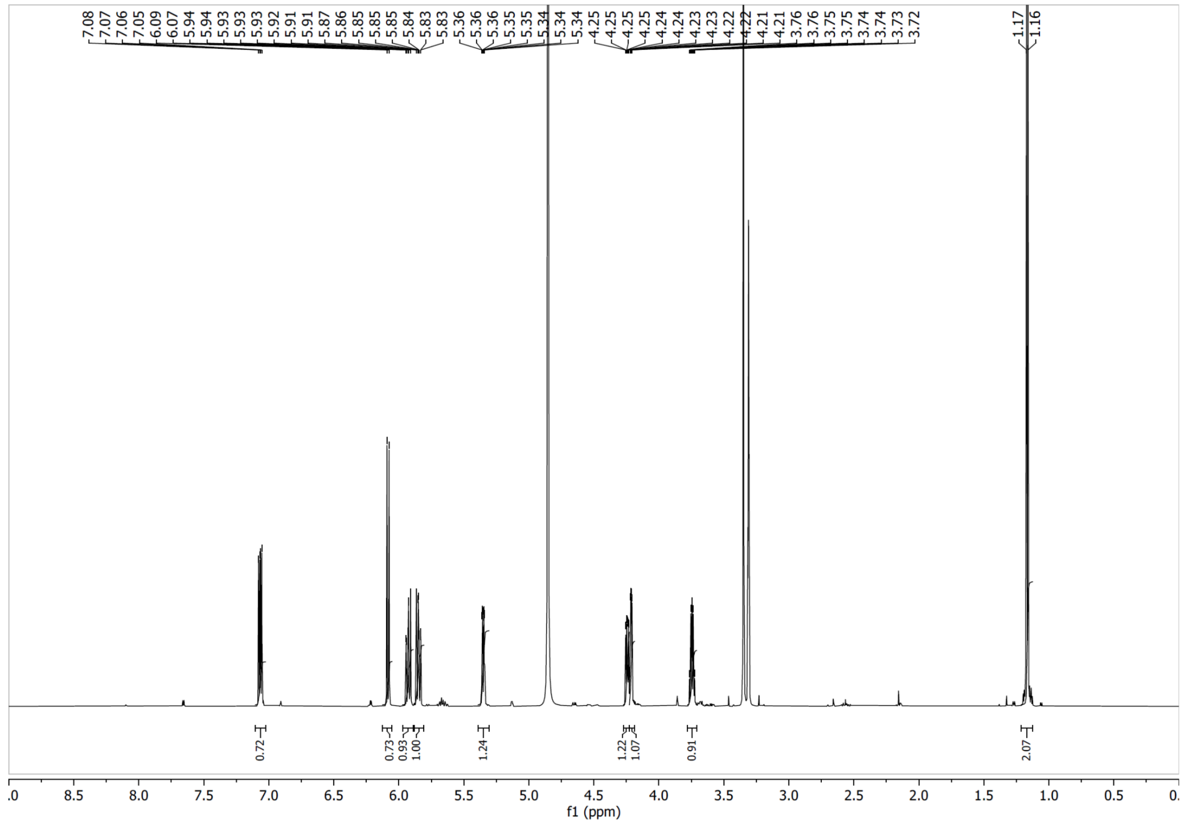

Figure S6. ^{1}H NMR spectrum of compound **2** in CD_3OD at 600 MHz

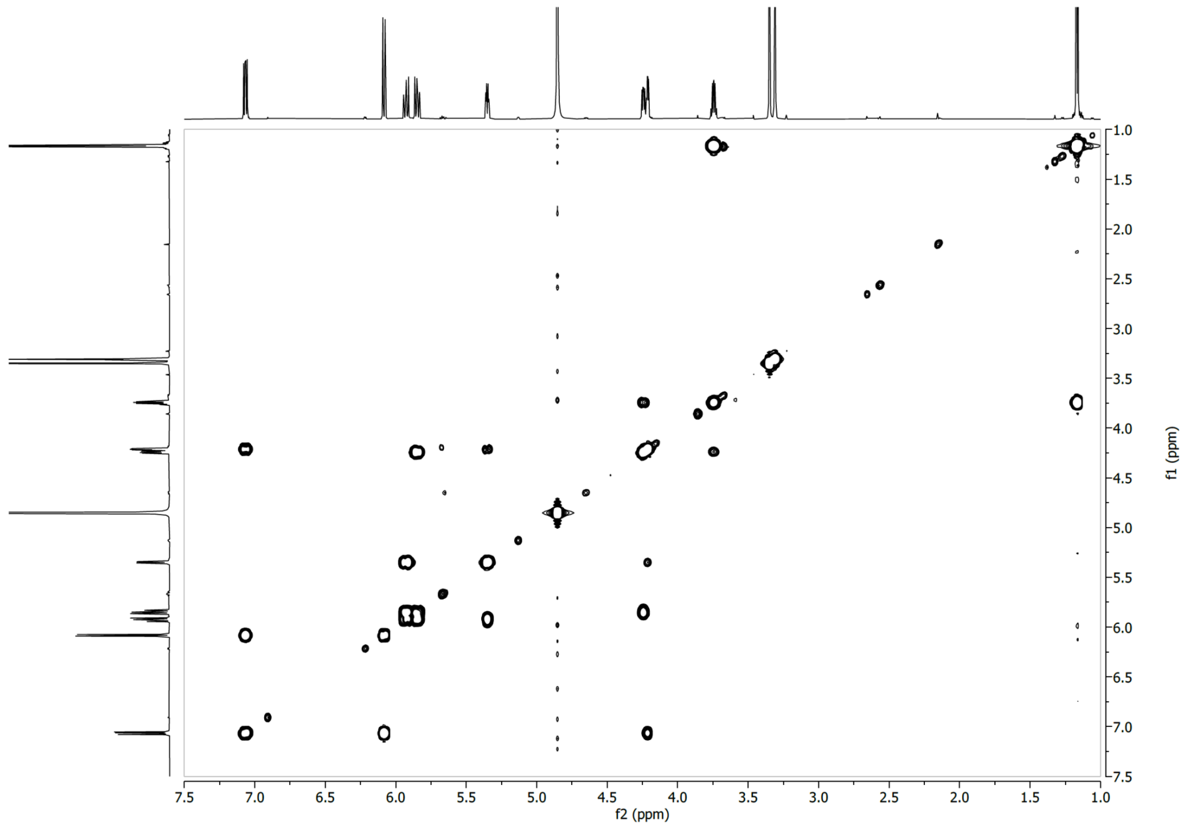

Figure S7. COSY NMR spectrum of compound **2** in CD3OD

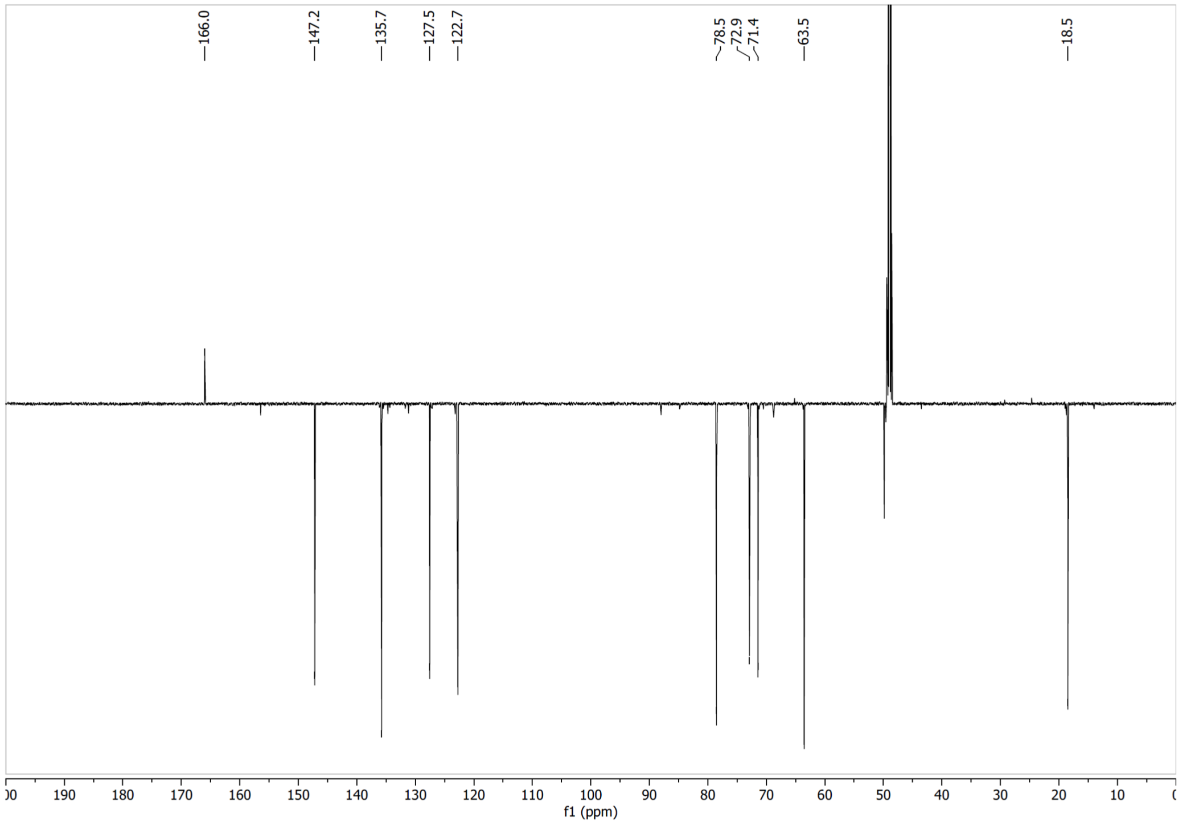

Figure S8. ^{13}C-DEPTQ NMR spectrum of compound **2** in CD_3OD at 151 MHz

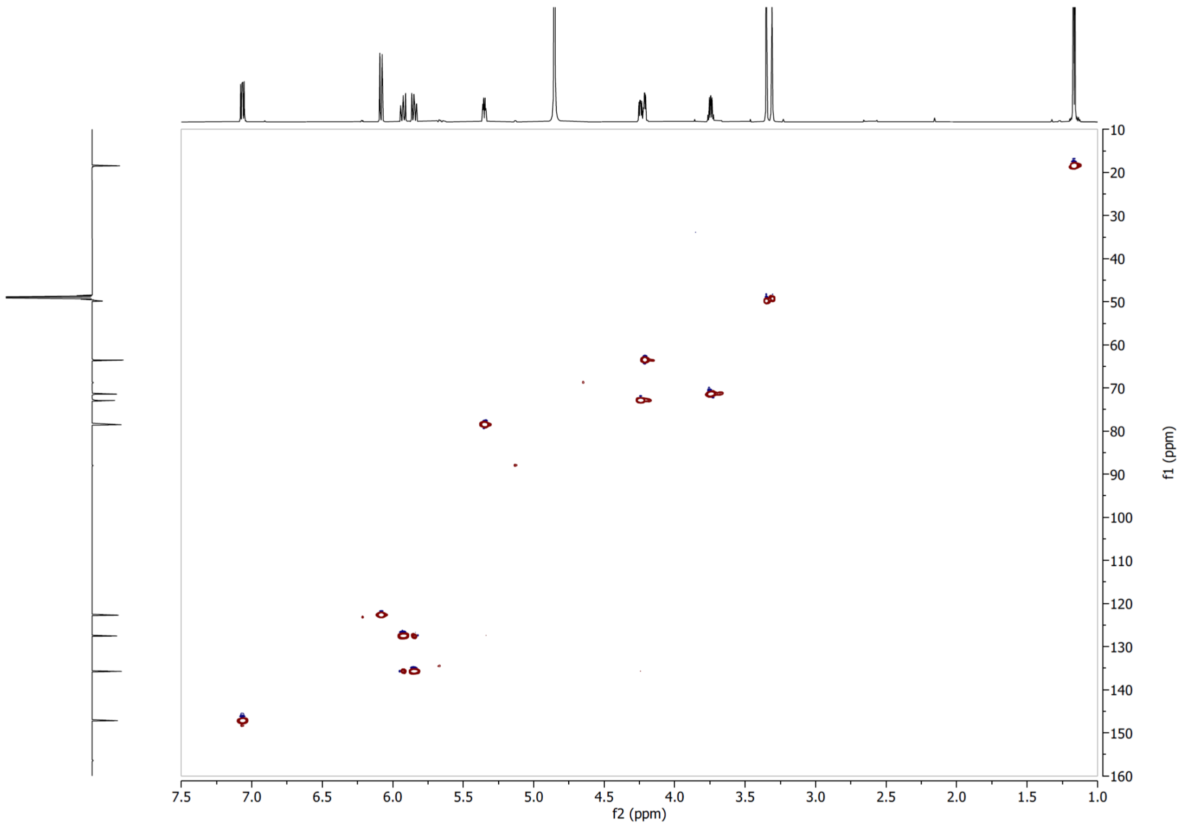

Figure S9. Edited-HSQC NMR spectrum of compound **2** in CD_3OD

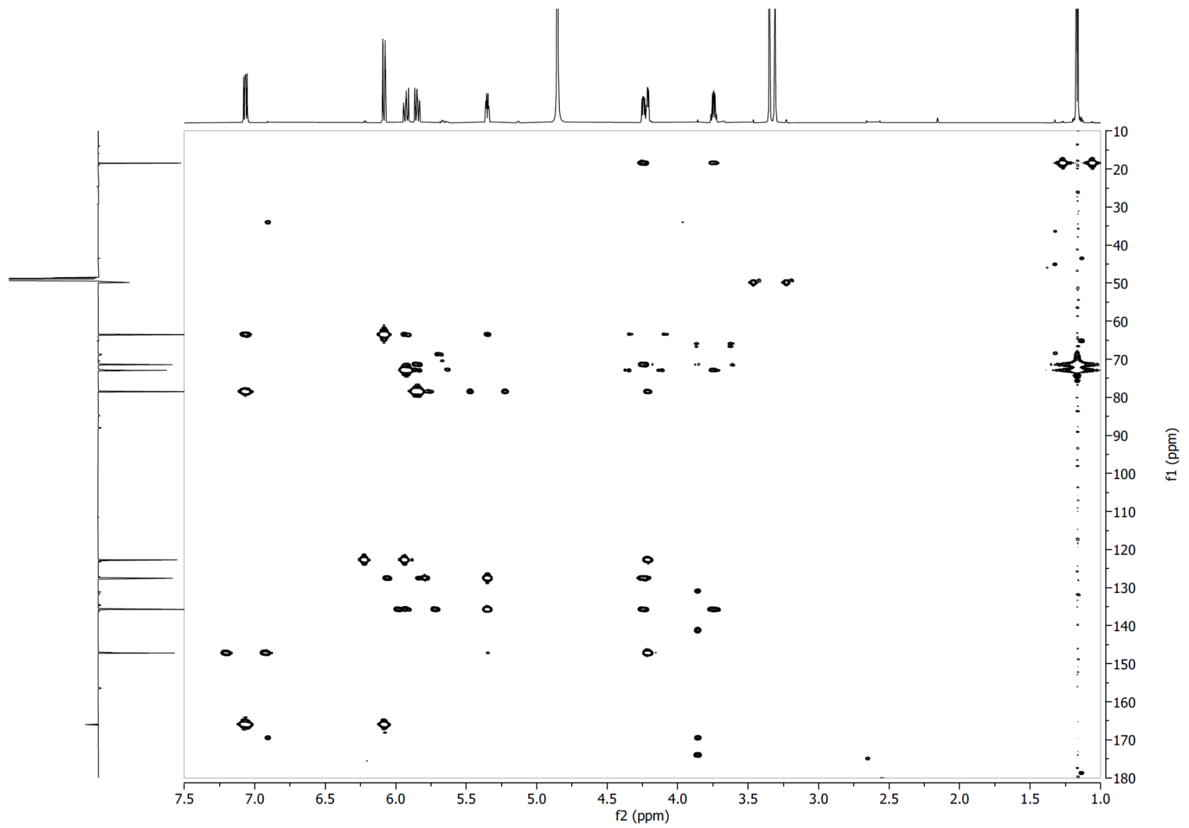

Figure S10. HMBC NMR spectrum of compound **2** in CD_3OD

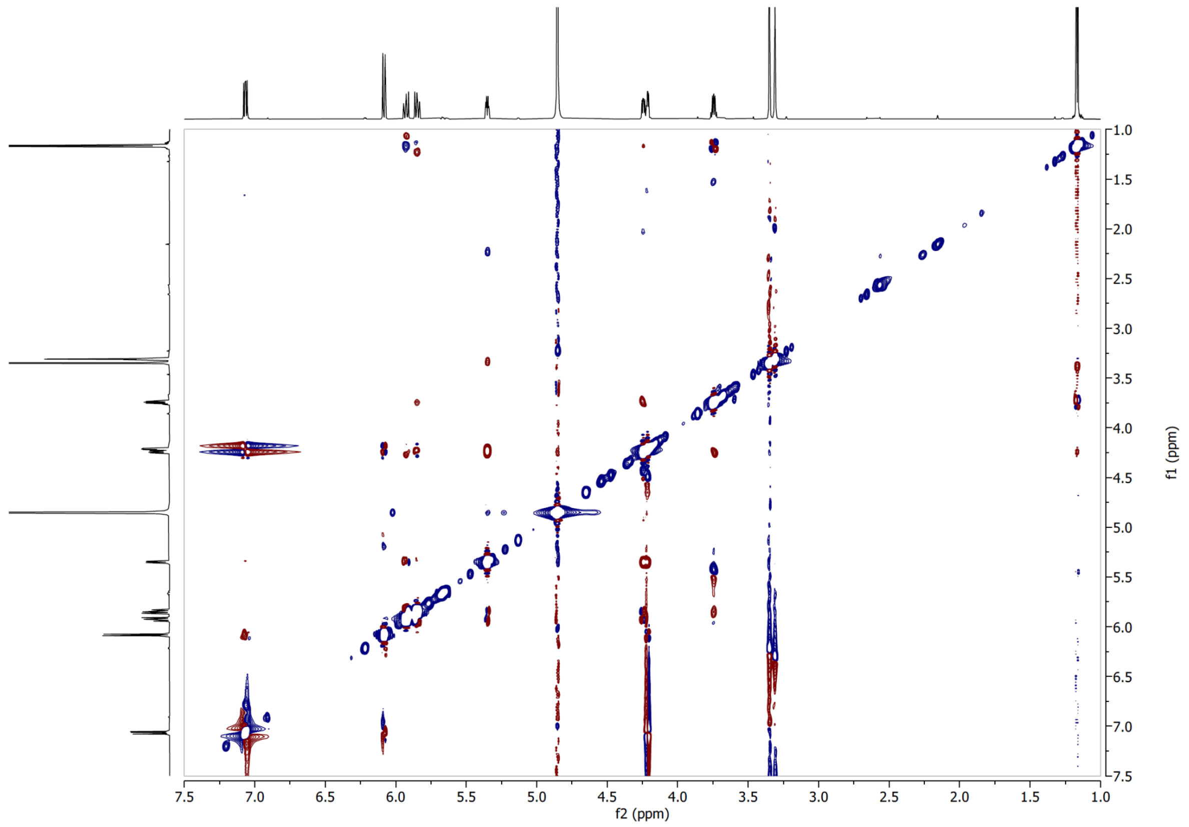

Figure S11. ROESY NMR spectrum of compound **2** in CD_3OD

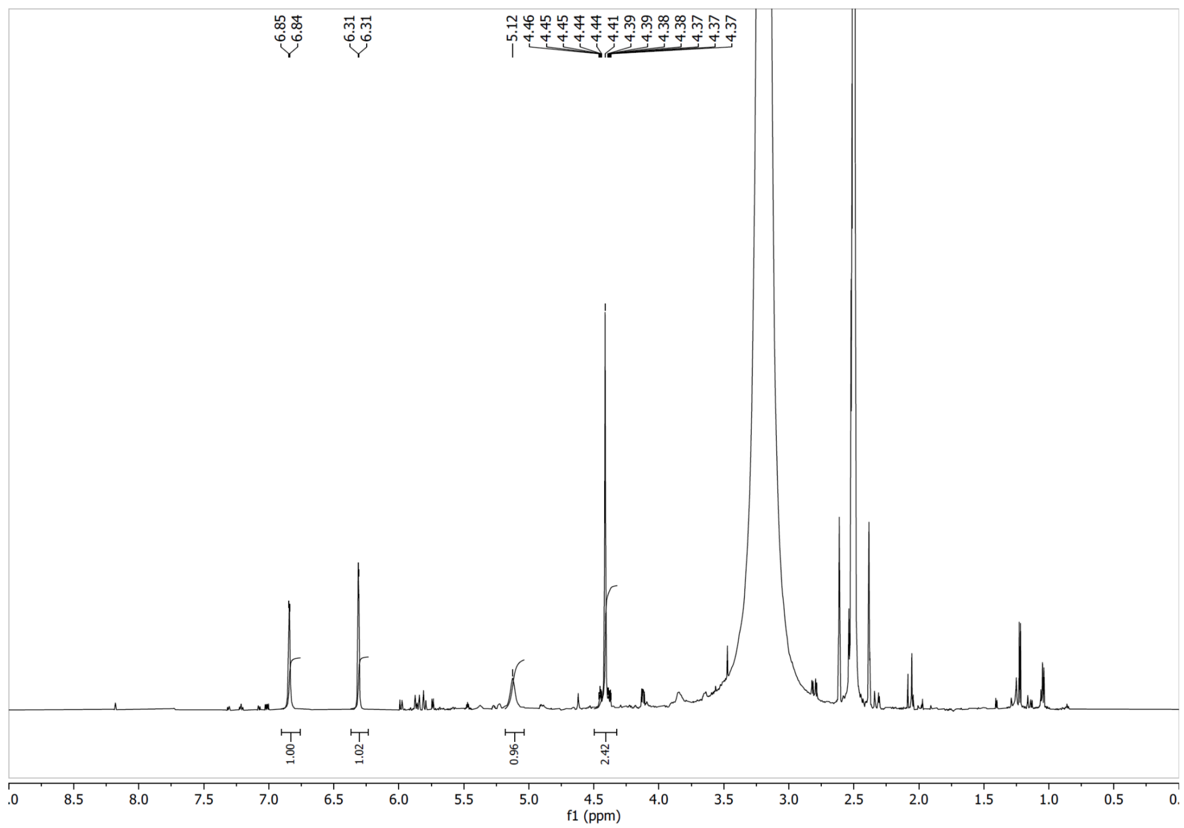

Figure S12. ^{1}H NMR spectrum of compound **3** in DMSO-d_6 at 600 MHz

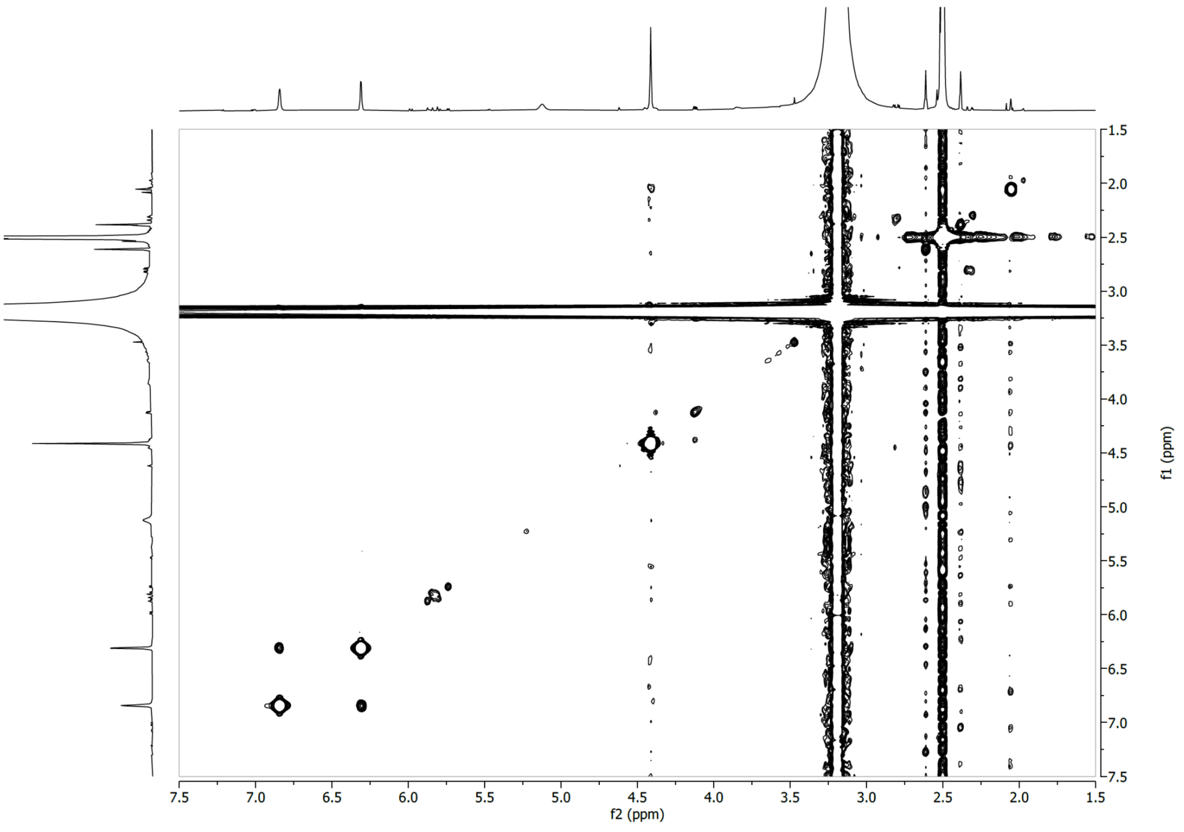

Figure S13. COSY NMR spectrum of compound **3** in DMSO-d_6

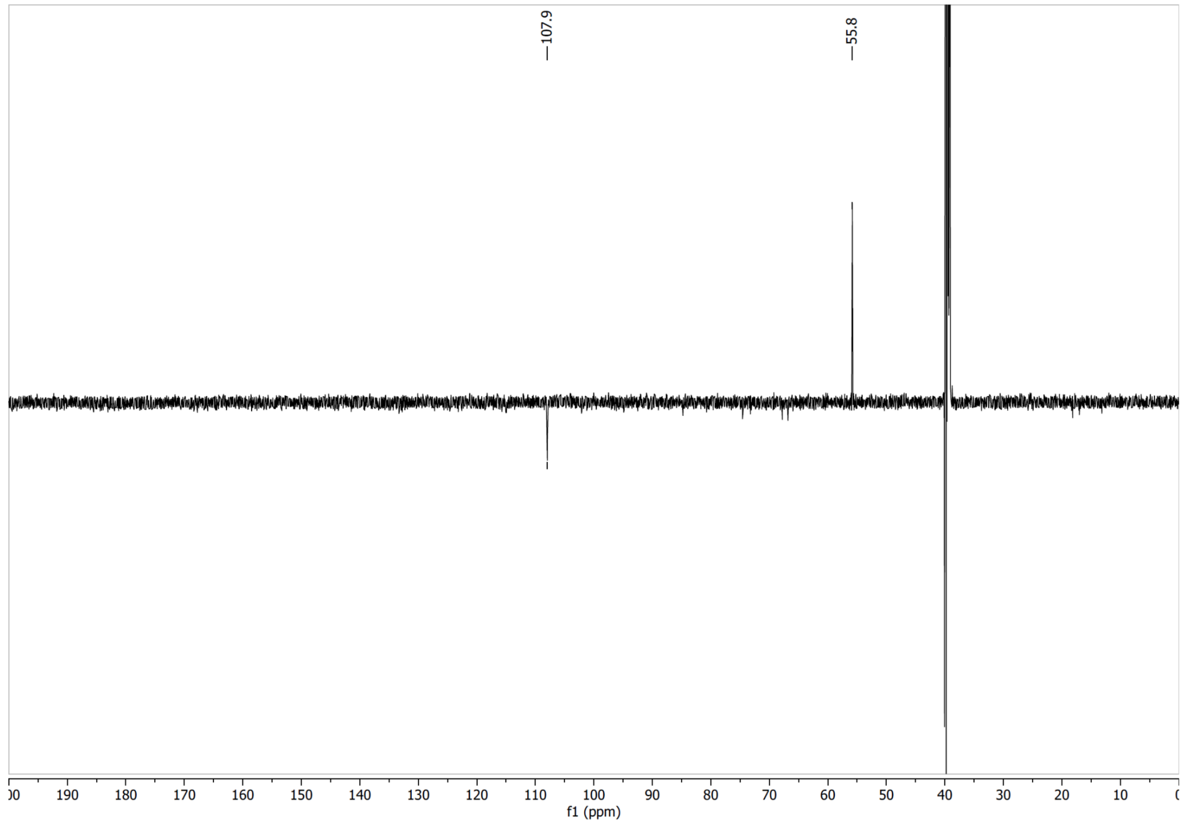

Figure S14. ^{13}C-DEPTQ NMR spectrum of compound **3** in DMSO-*d*$_6$

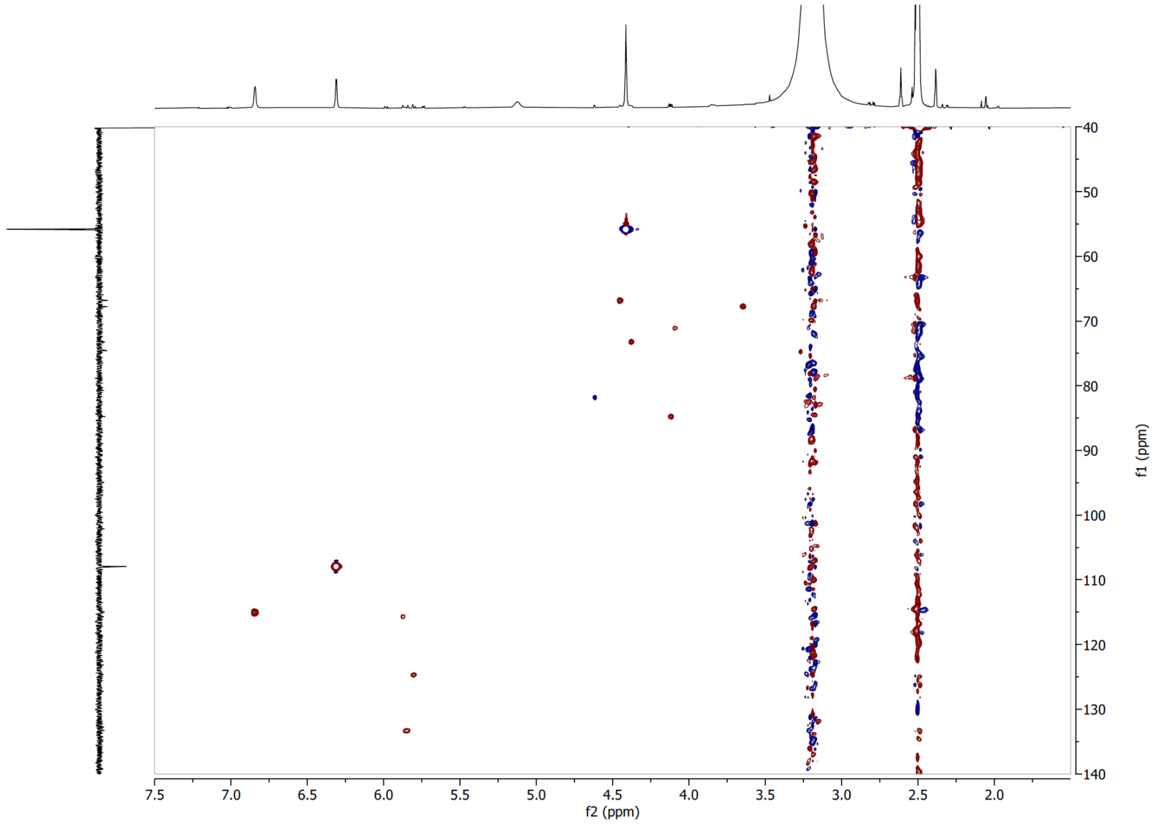

Figure S15. Edited-HSQC NMR spectrum of compound **3** in DMSO-*d*$_6$

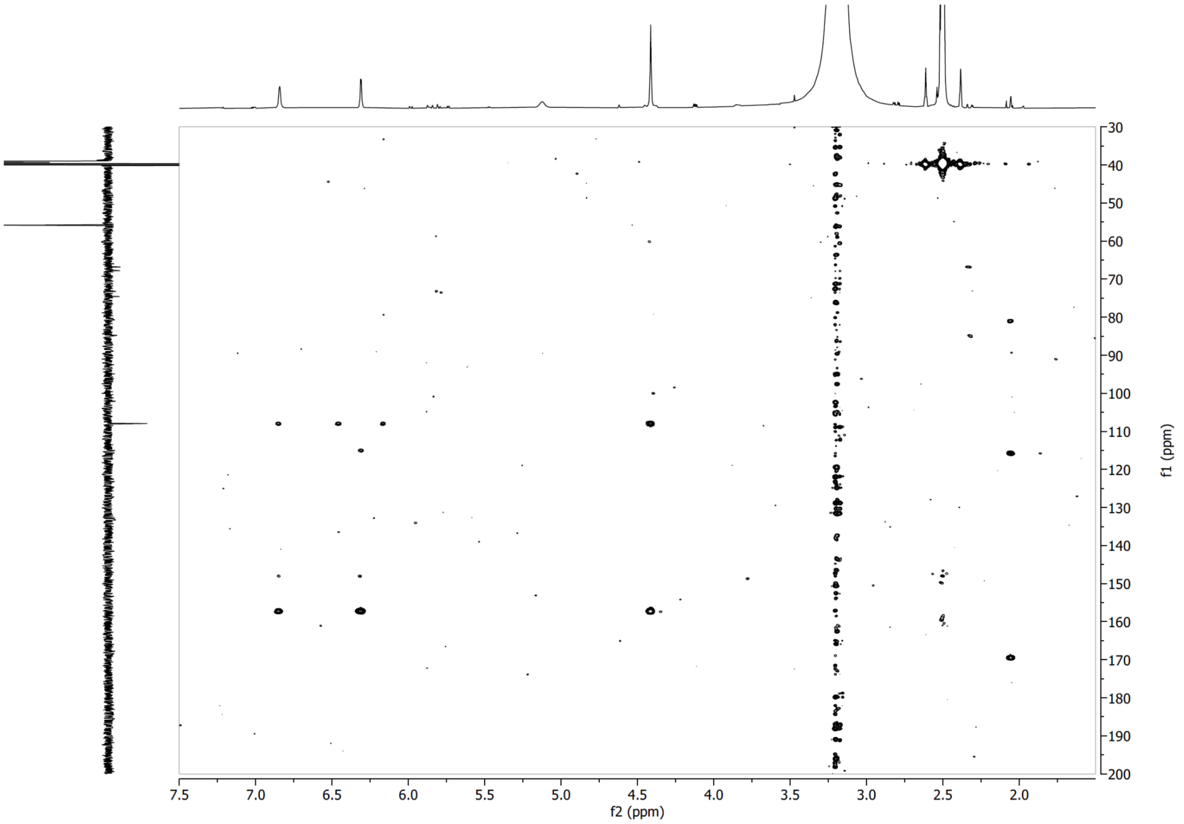

Figure S16. HMBC NMR spectrum of compound **3** in DMSO-d_6

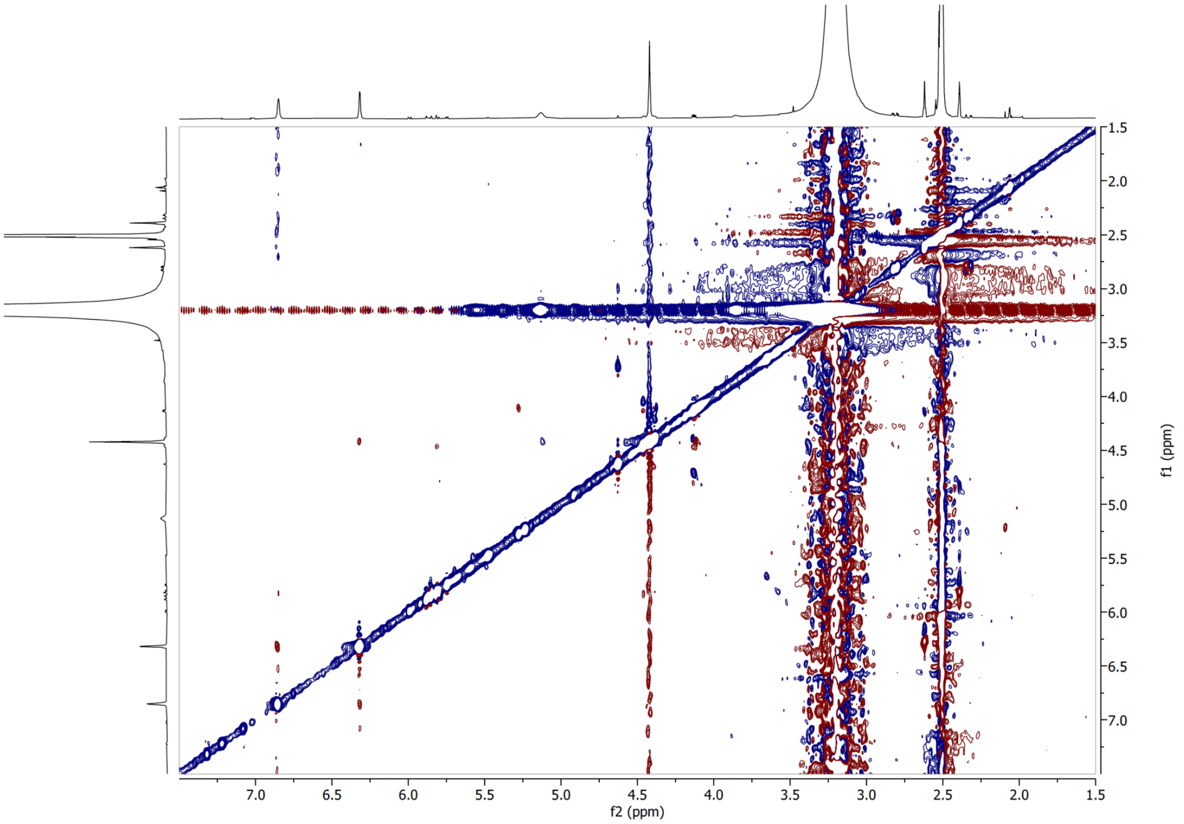

Figure S17. ROESY NMR spectrum of compound **3** in DMSO-d_6

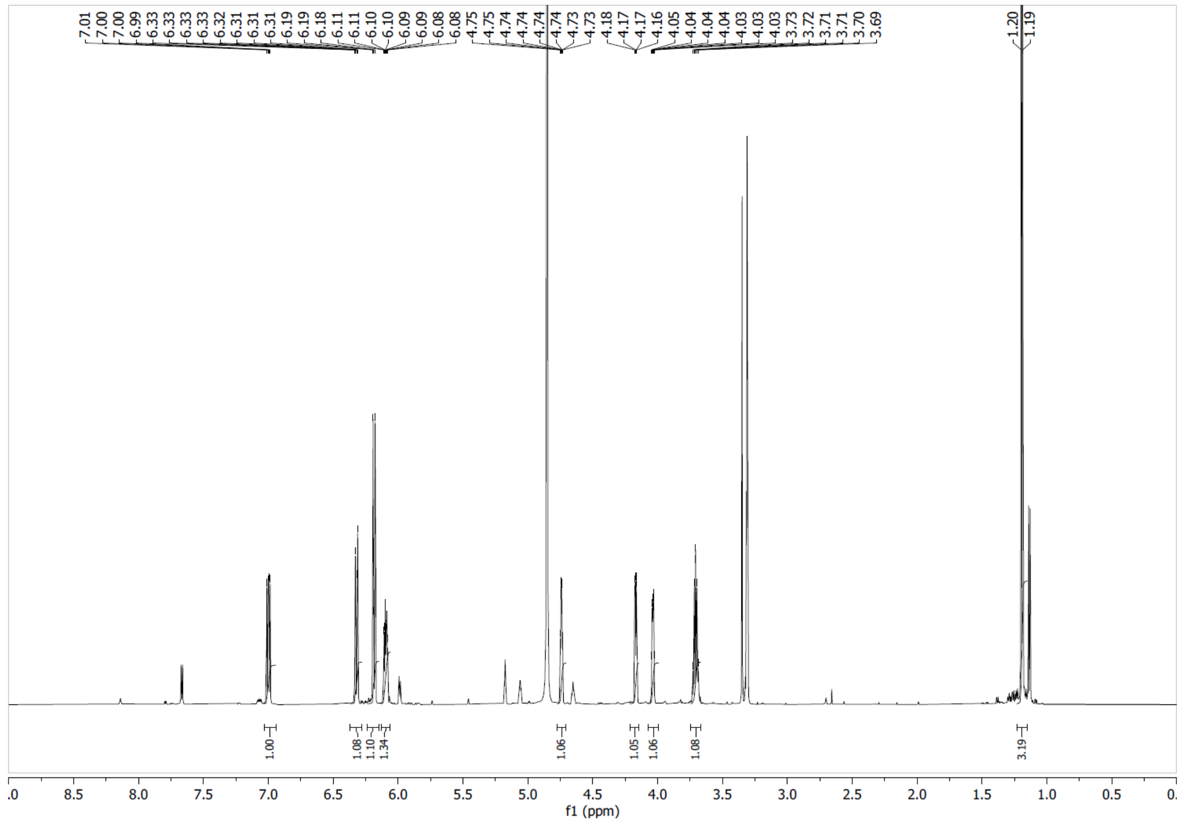

Figure S18. ^{1}H NMR spectrum of compound **4** in CD_3OD

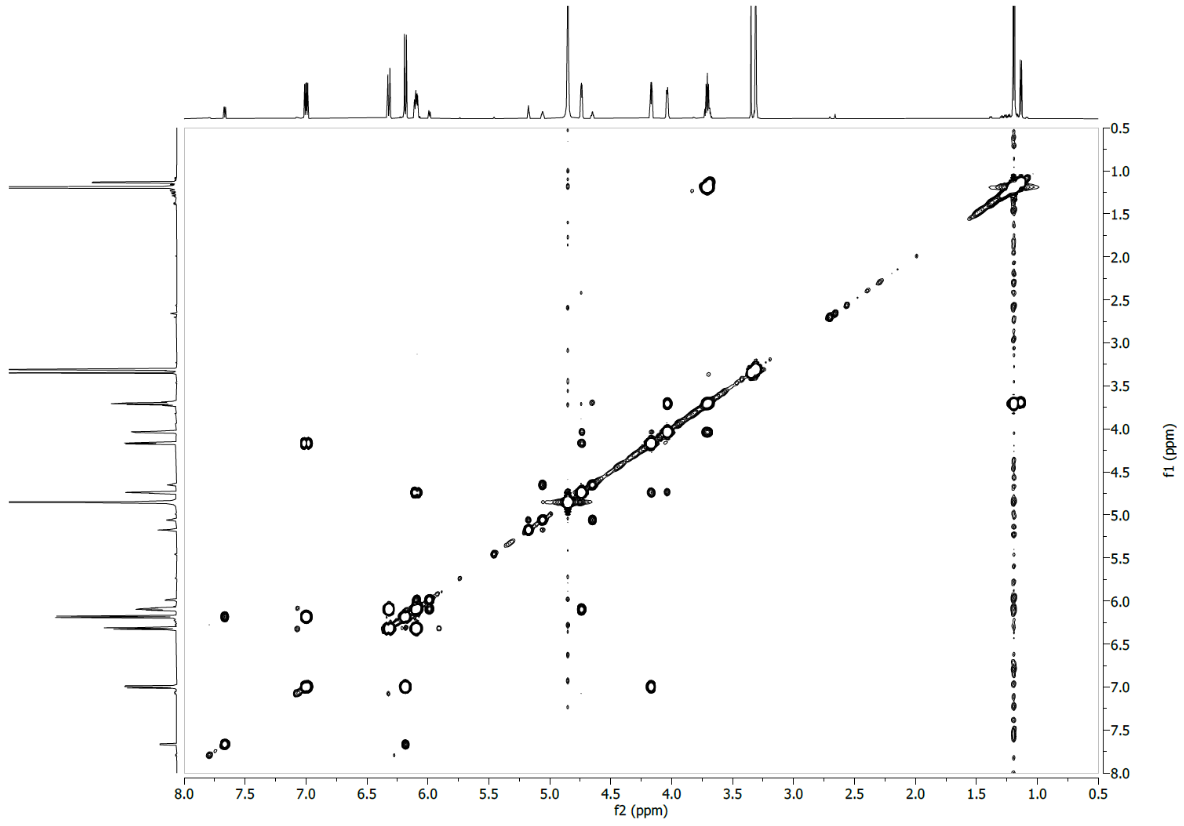

Figure S19. COSY NMR spectrum of compound **4** in CD_3OD

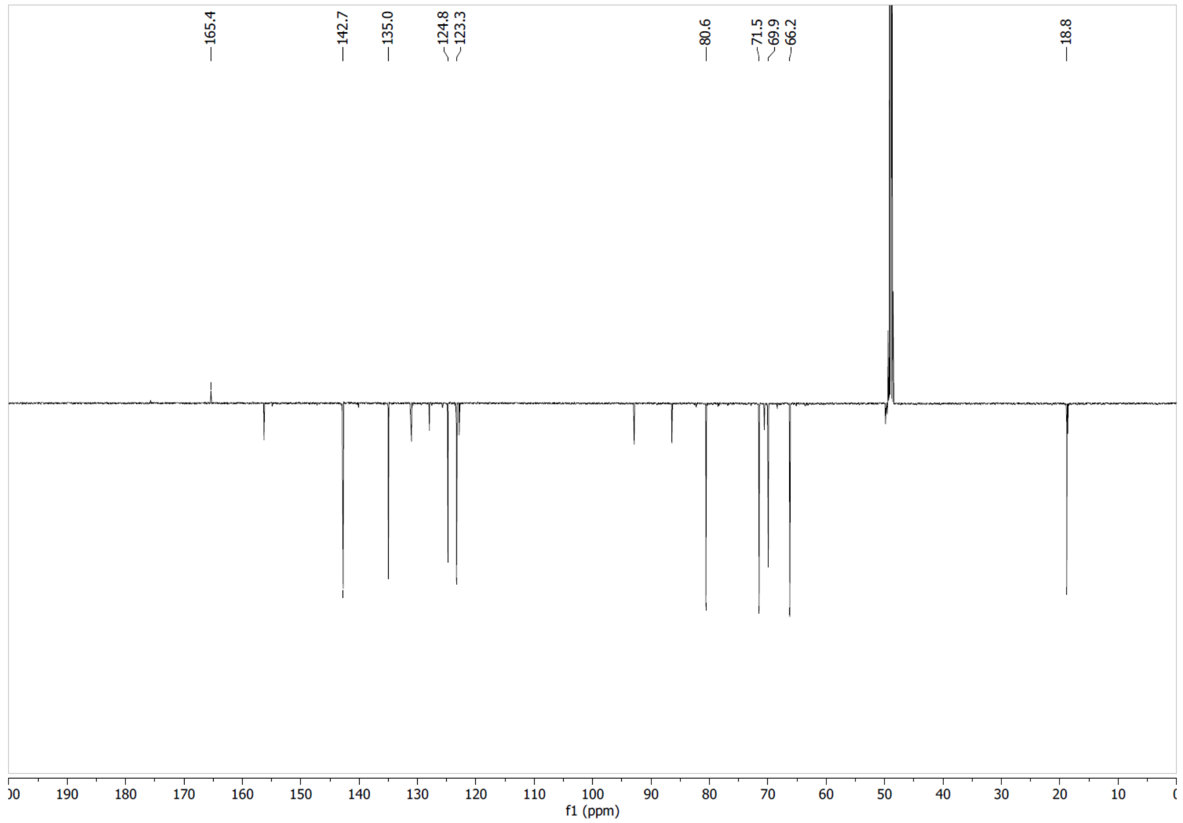

Figure S20. ^{13}C-DEPTQ NMR spectrum of compound **4** in CD_3OD at 151 MHz

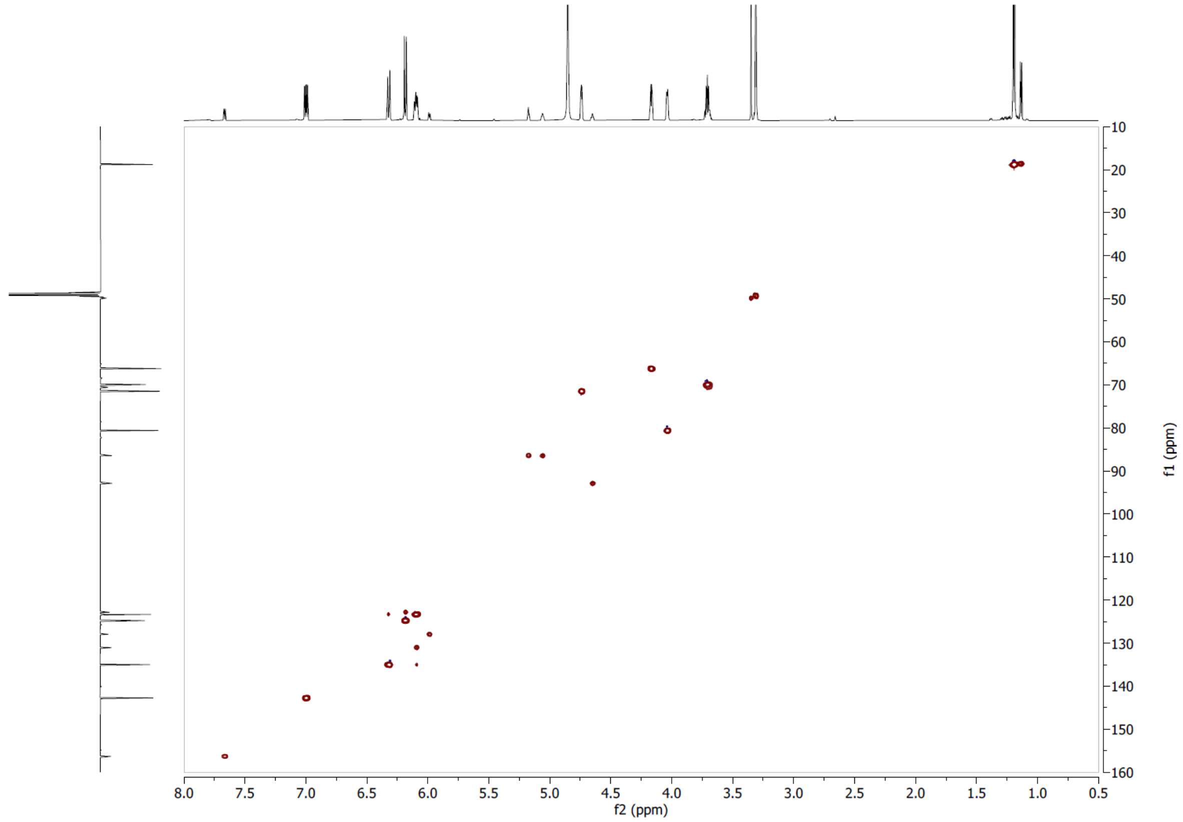

Figure S21. Edited-HSQC NMR spectrum of compound **4** in CD_3OD

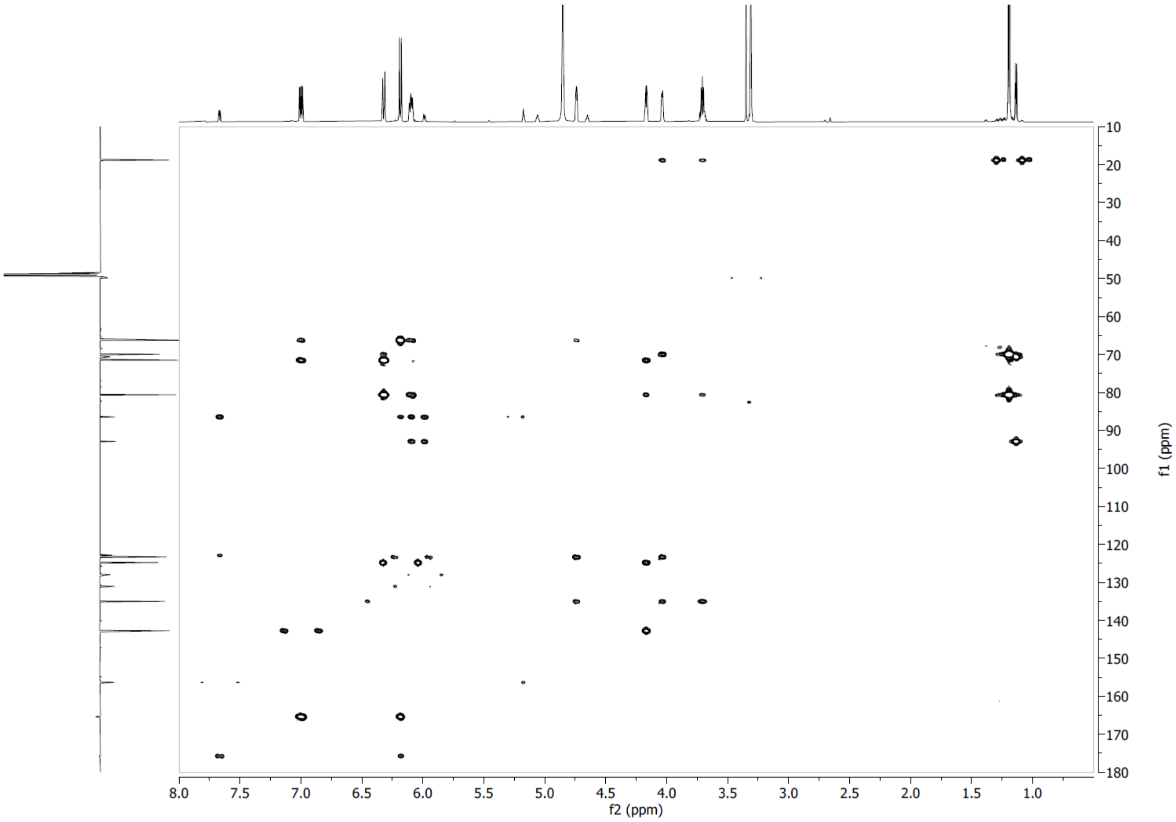

Figure S22. HMBC NMR spectrum of compound **4** in CD_3OD

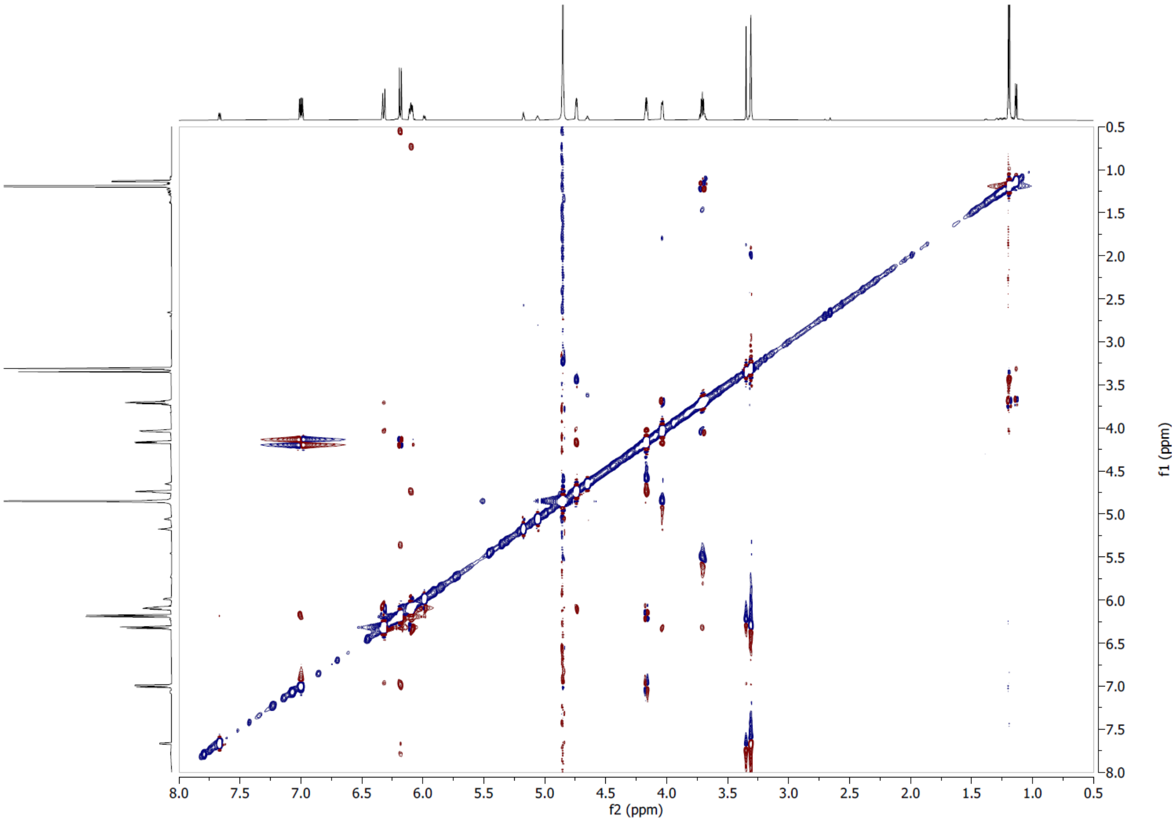

Figure S23. ROESY NMR spectrum of compound **4** in CD_3OD

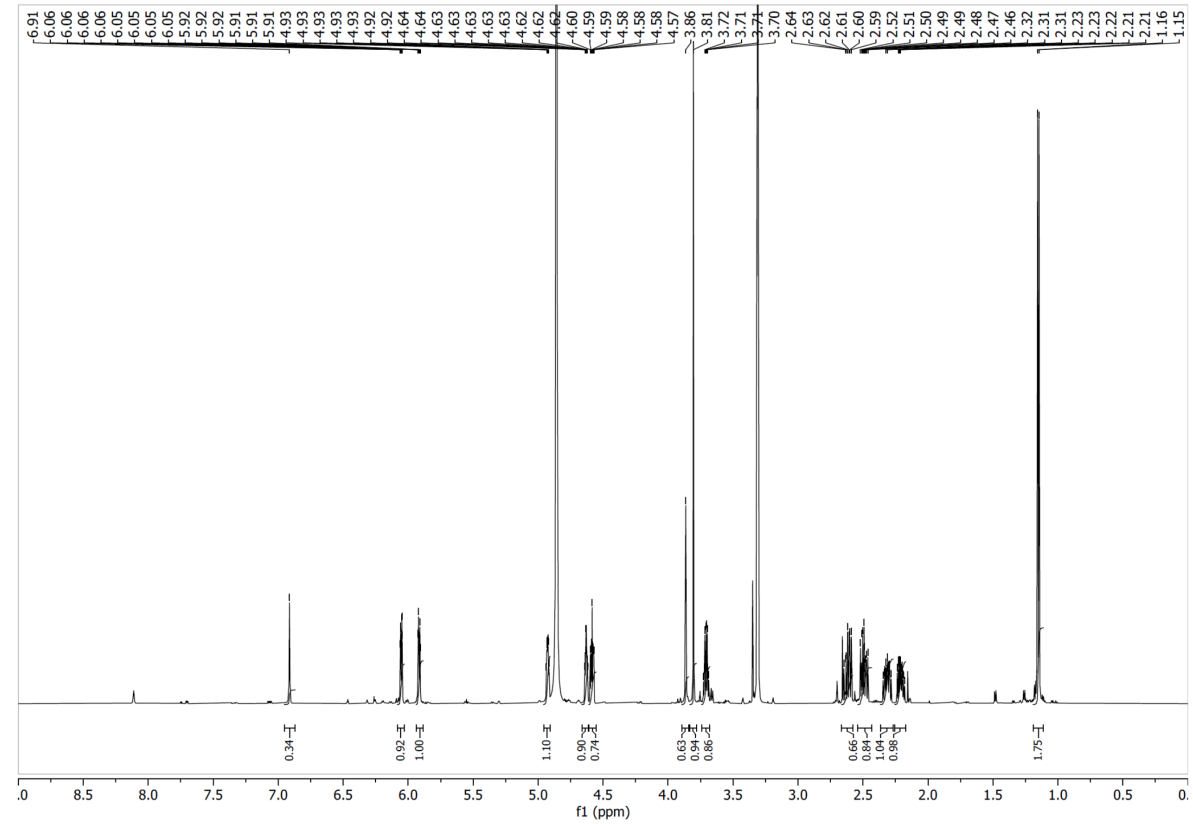

Figure S24. ^1H NMR spectrum of compound **5** in CD_3OD at 600 MHz

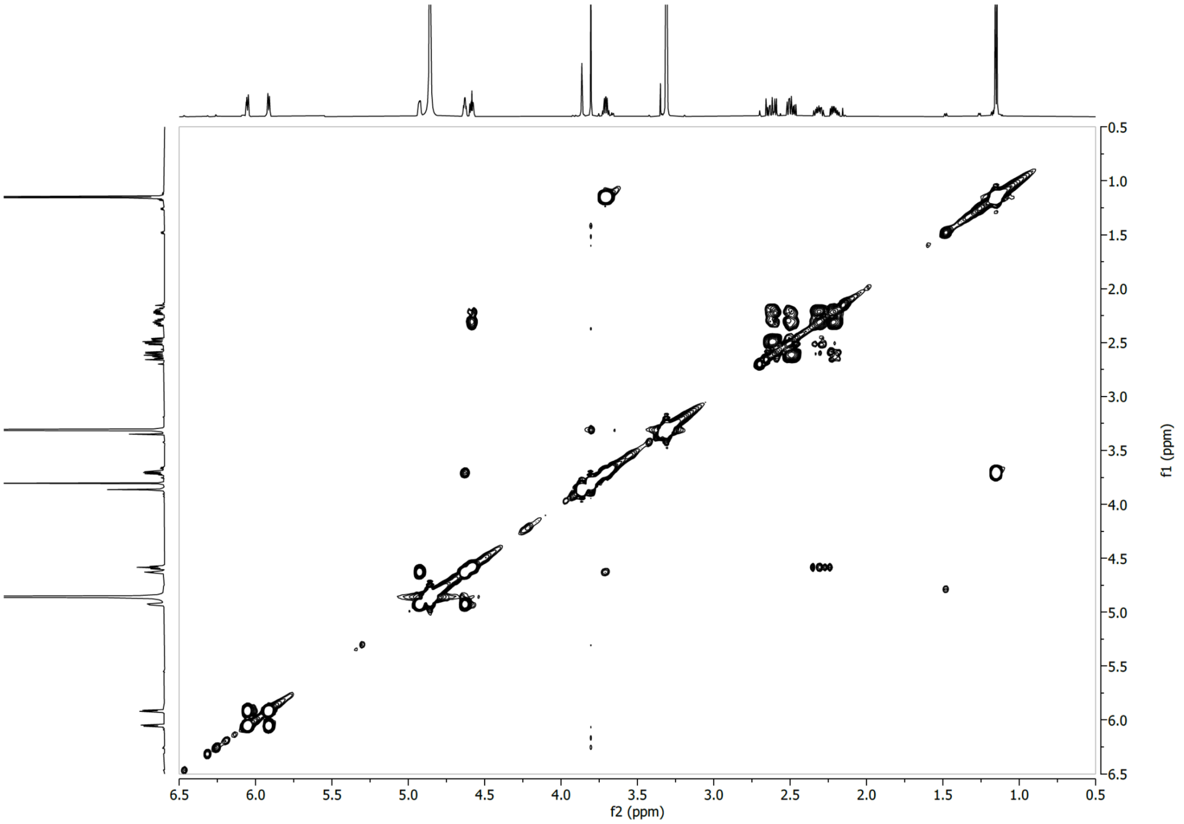

Figure S25. COSY NMR spectrum of compound **5** in CD_3OD

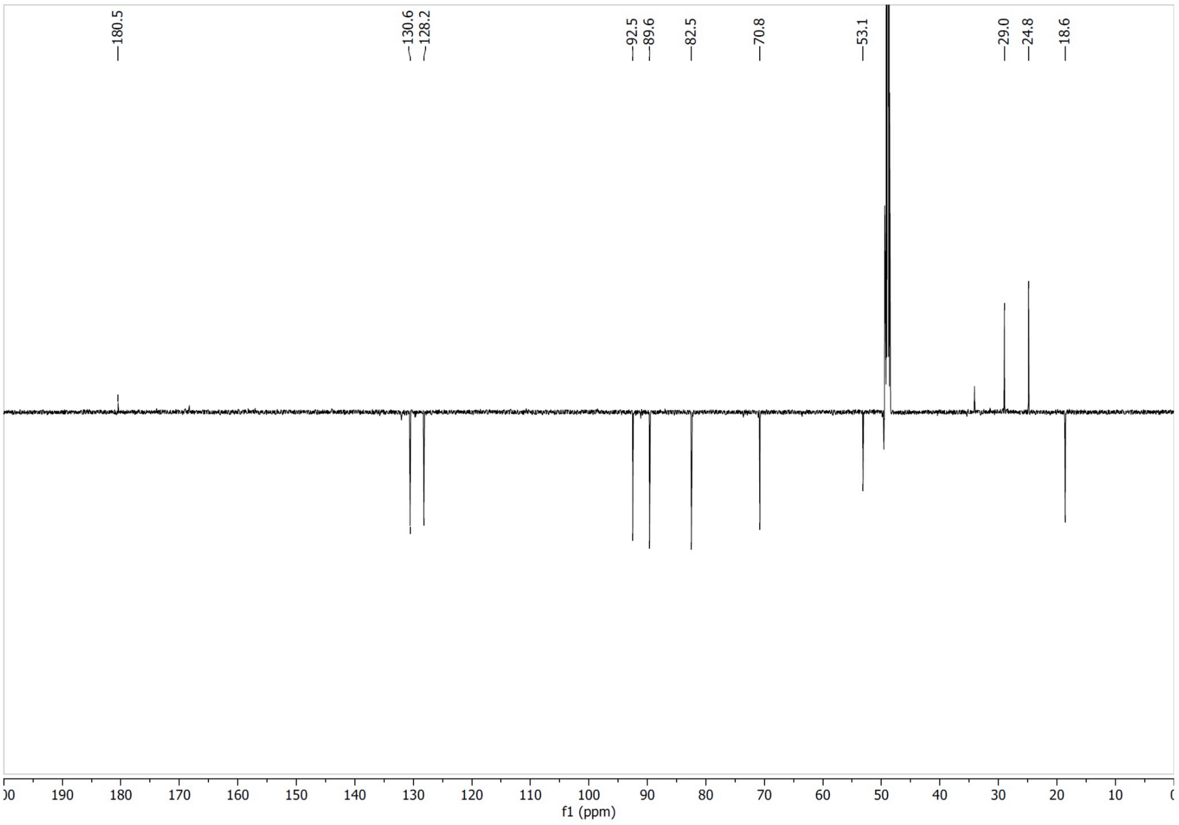

Figure S26. ^{13}C-DEPTQ NMR spectrum of compound **5** in CD_3OD at 156 MHz

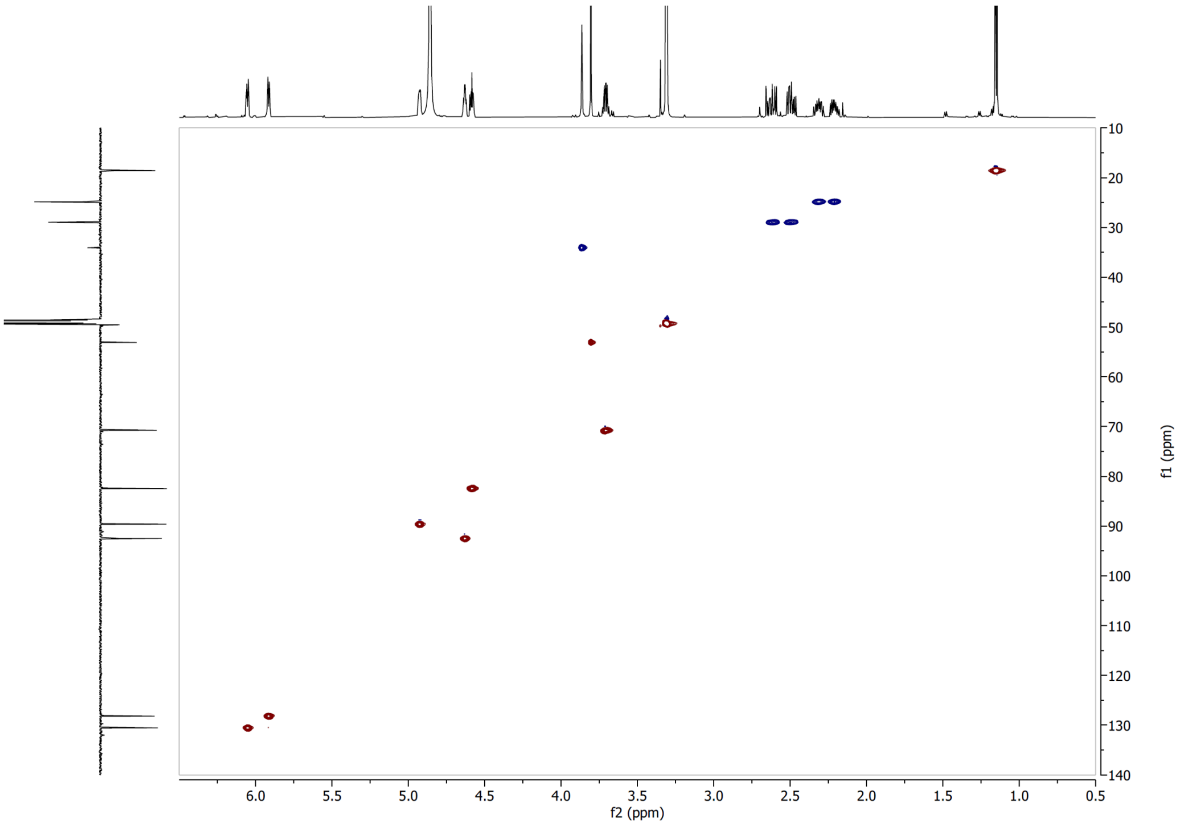

Figure S27. Edited-HSQC NMR spectrum of compound **5** in CD_3OD

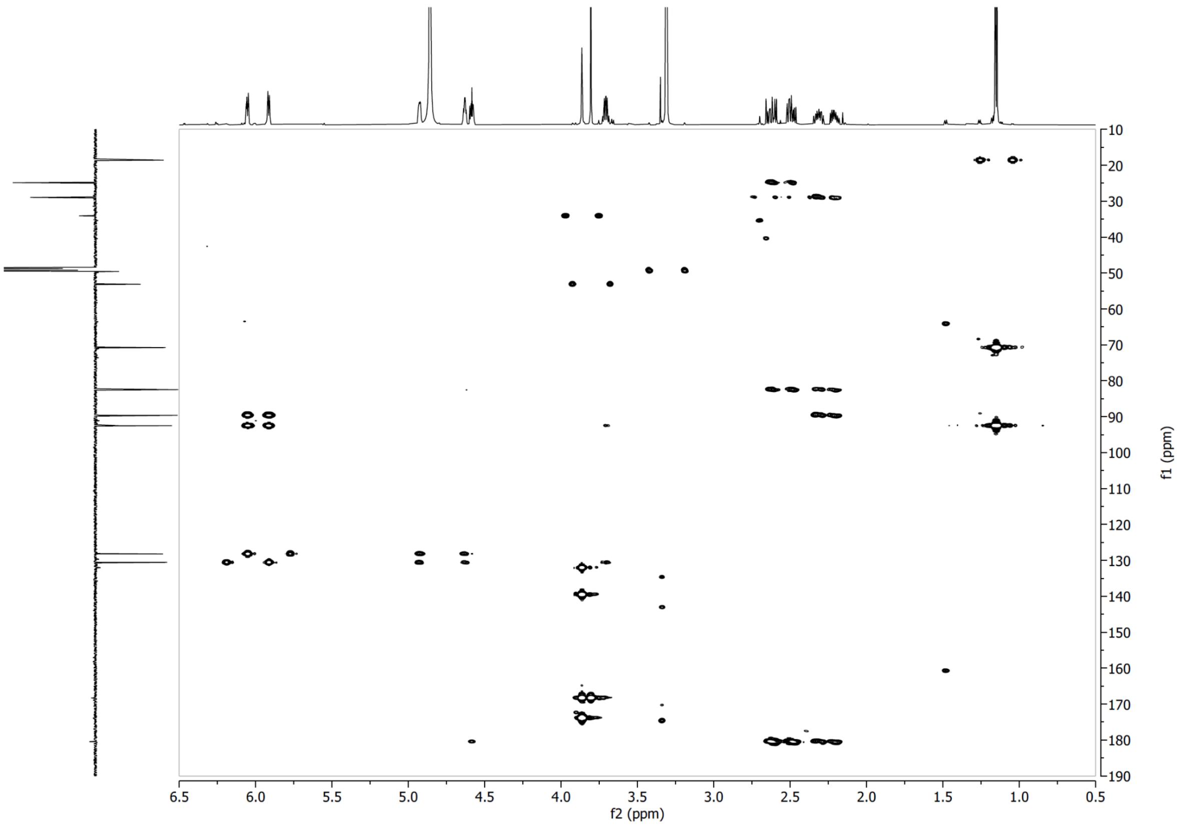

Figure S28. HMBC NMR spectrum of compound **5** in CD_3OD

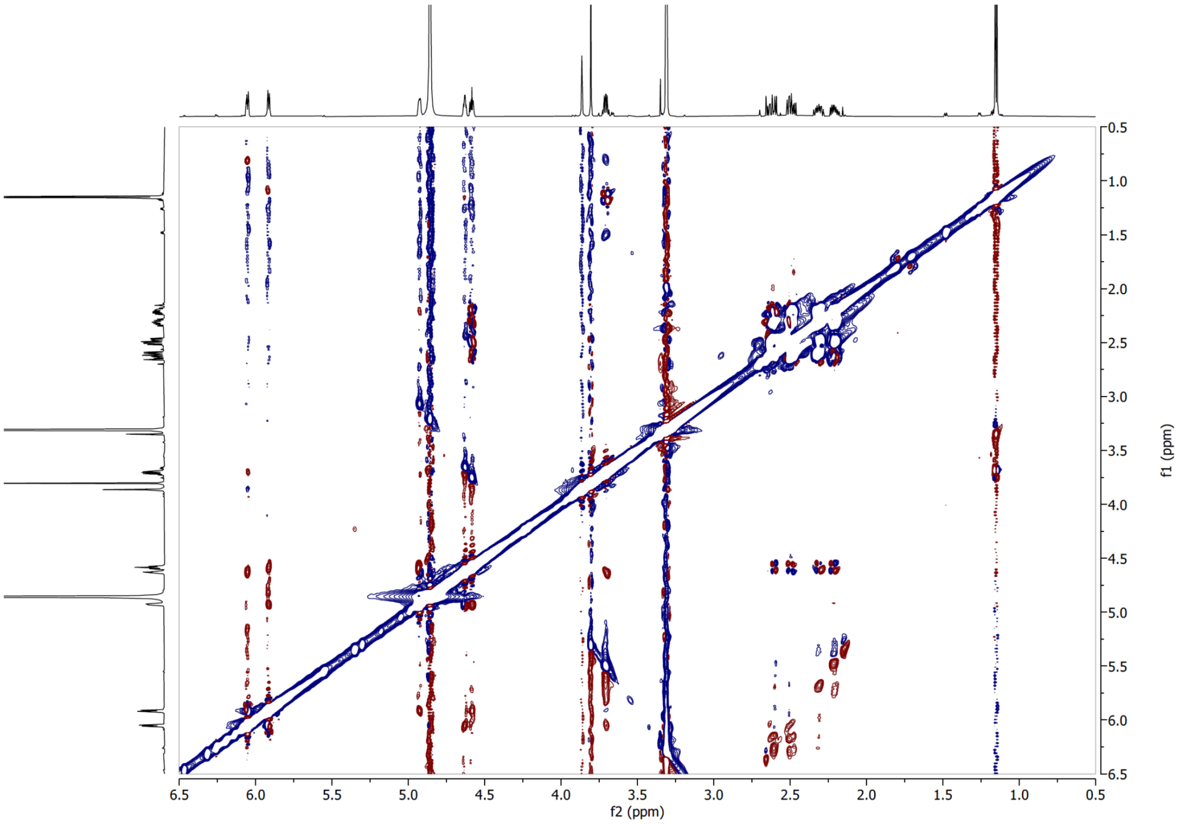

Figure S29. ROESY NMR spectrum of compound **5** in CD_3OD

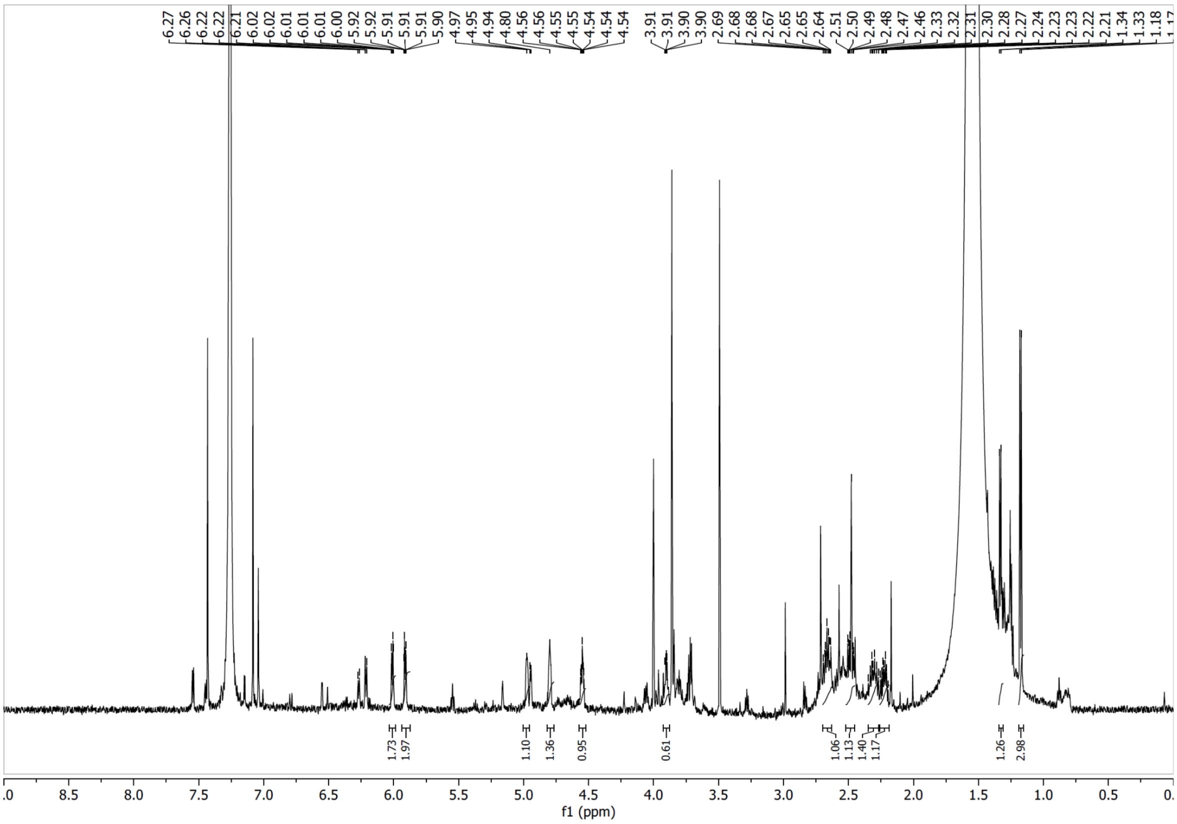

Figure S30. 1H NMR spectrum of compound **5** in CDCl3 at 600 MHz

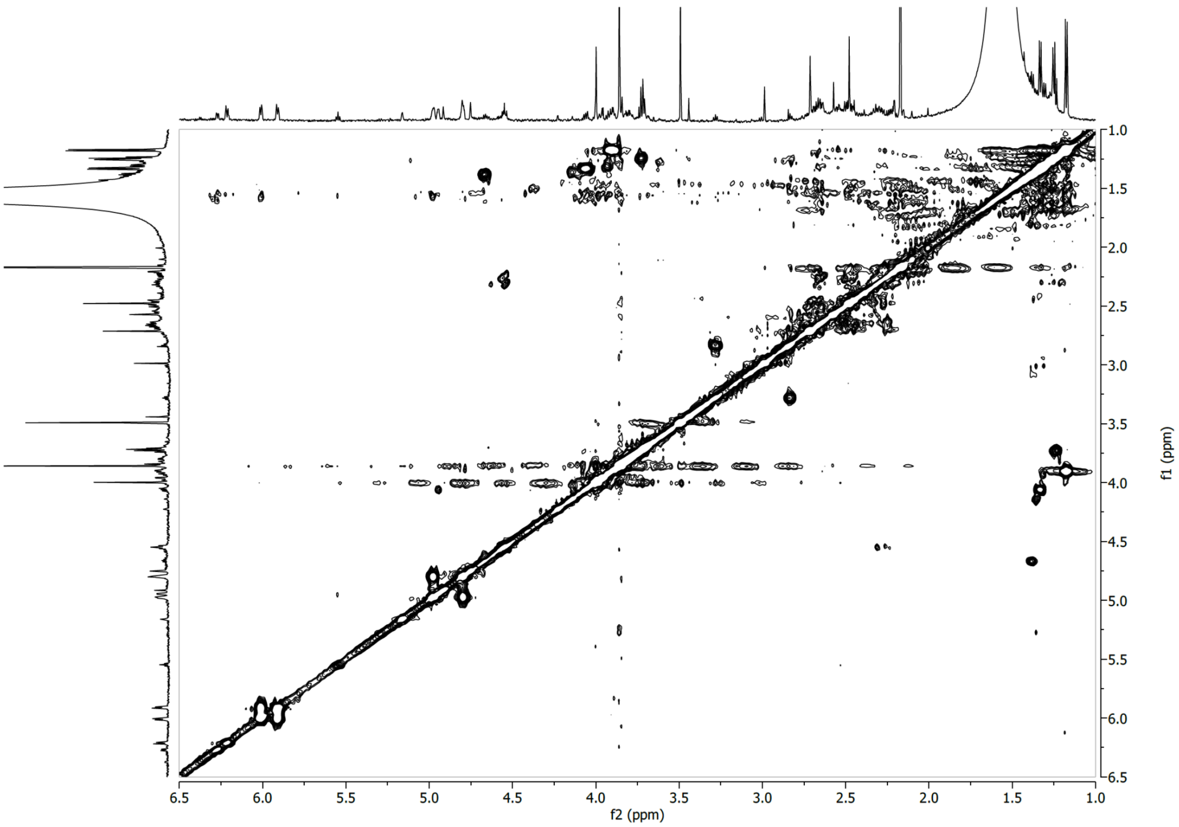

Figure S31. COSY NMR spectrum of compound **5** in $CDCl_3$

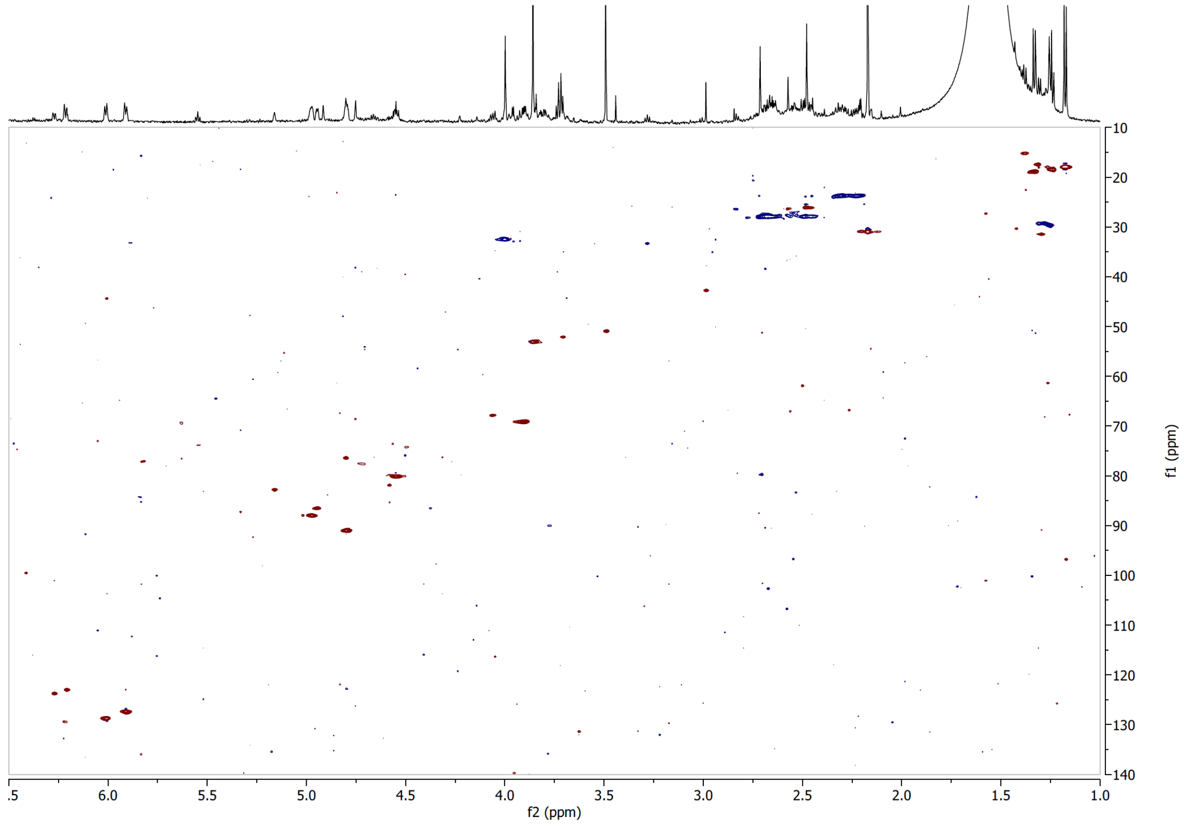

Figure S32. Edited-HSQC NMR spectrum of compound **5** in $CDCl_3$

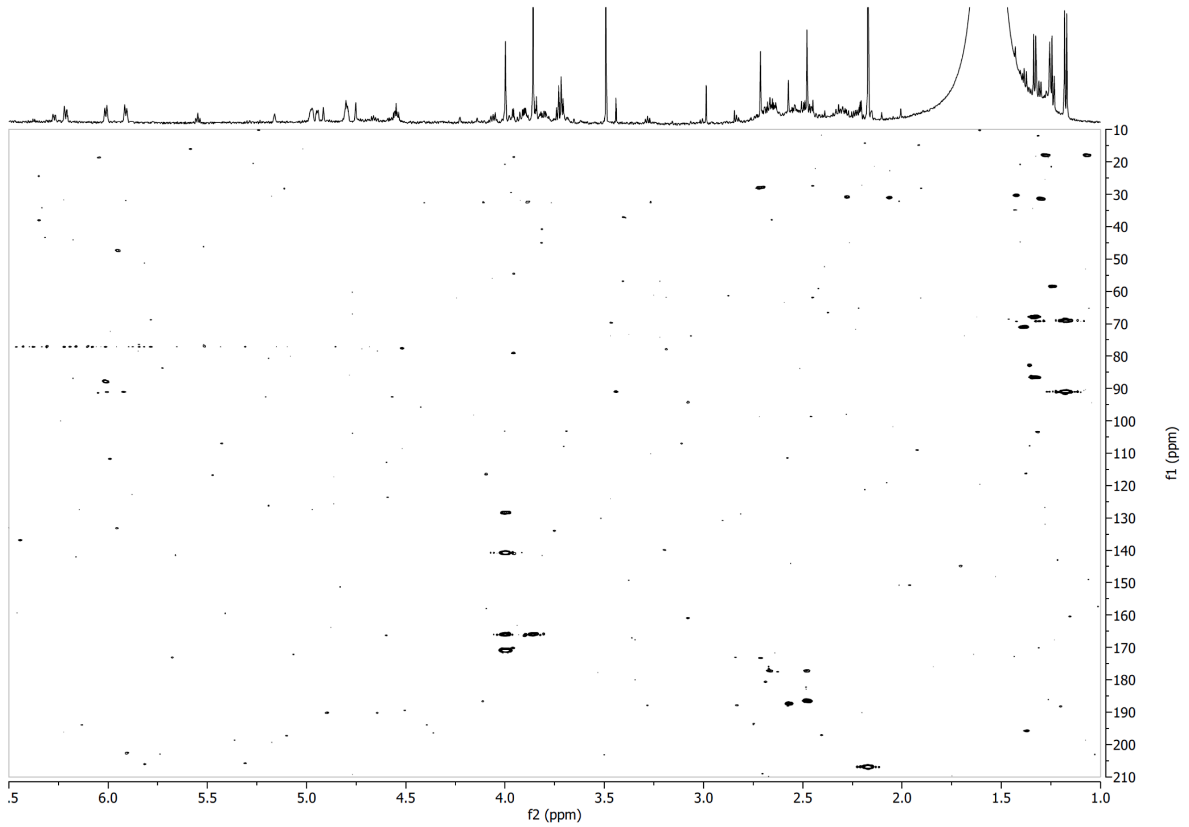

Figure S33. HMBC NMR spectrum of compound **5** in $CDCl_3$

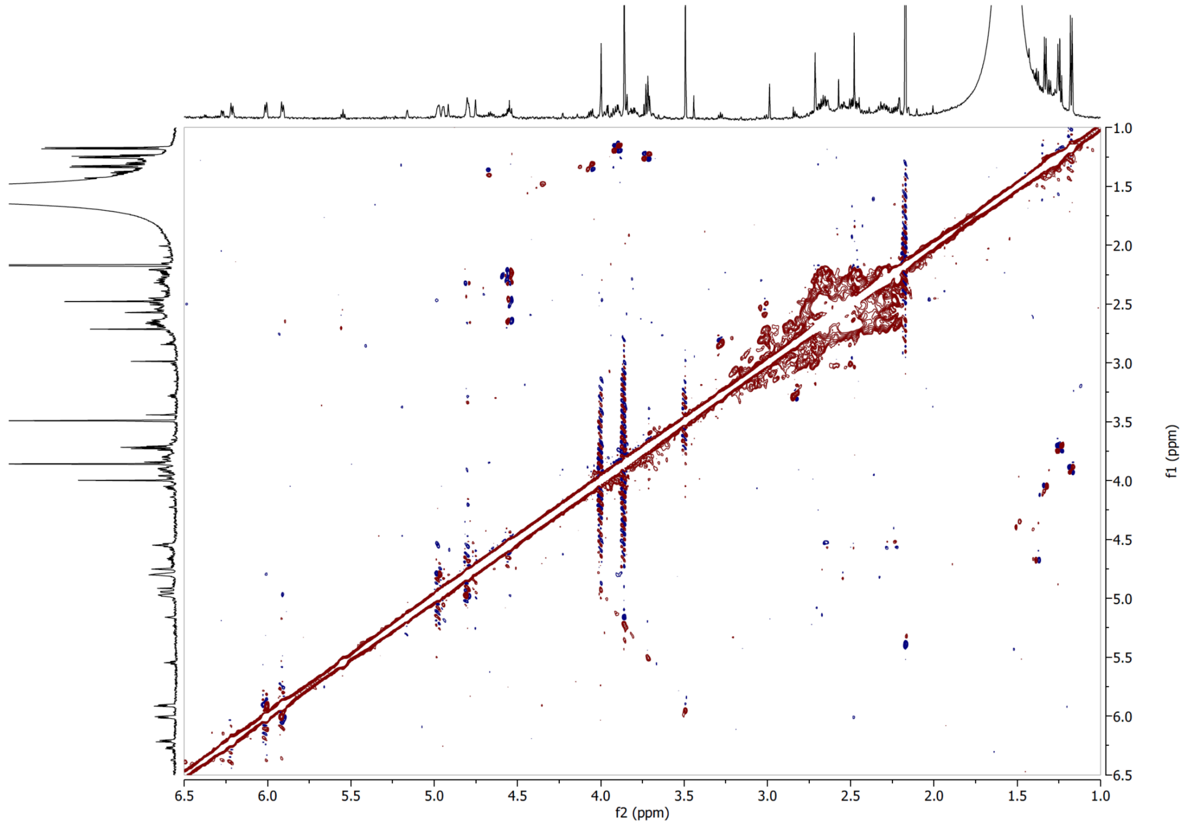

Figure S34. ROESY NMR spectrum of compound **5** in $CDCl_3$

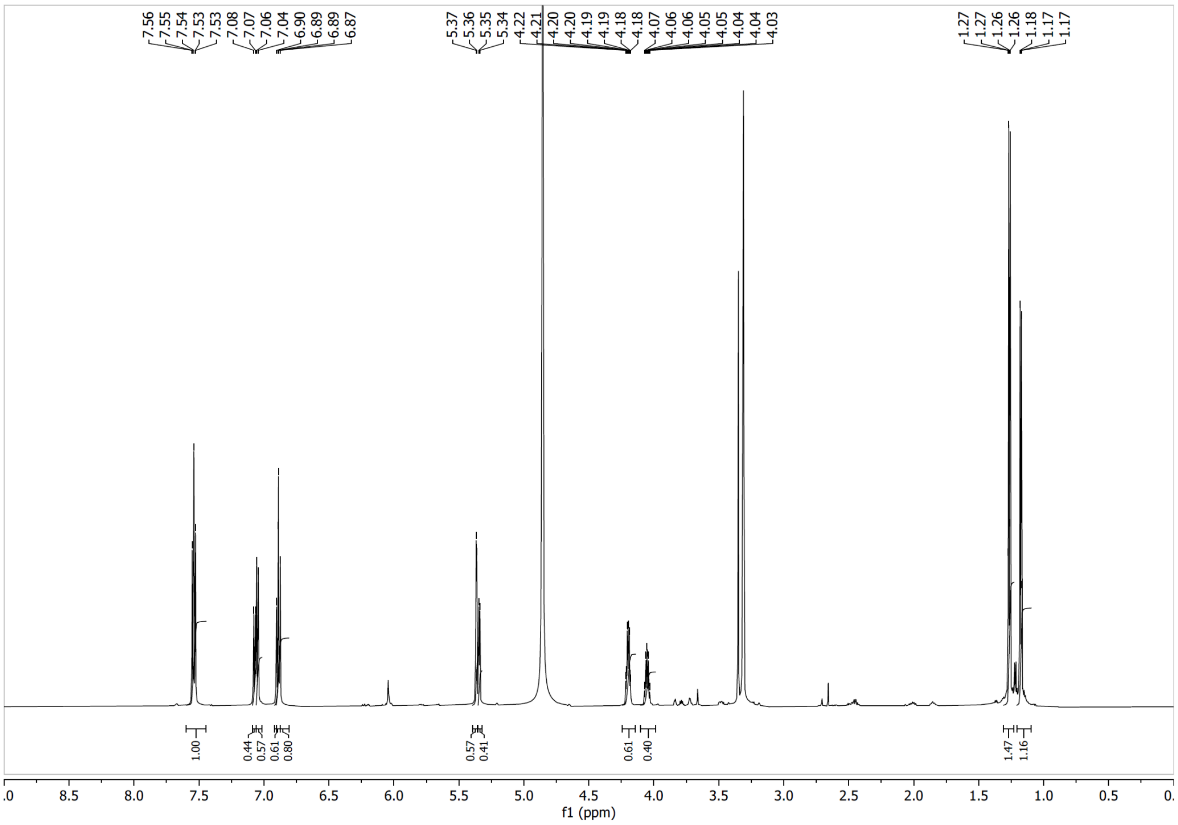

Figure S35. ^{1}H NMR spectrum of compound **6a** and **6b** in CD_3OD

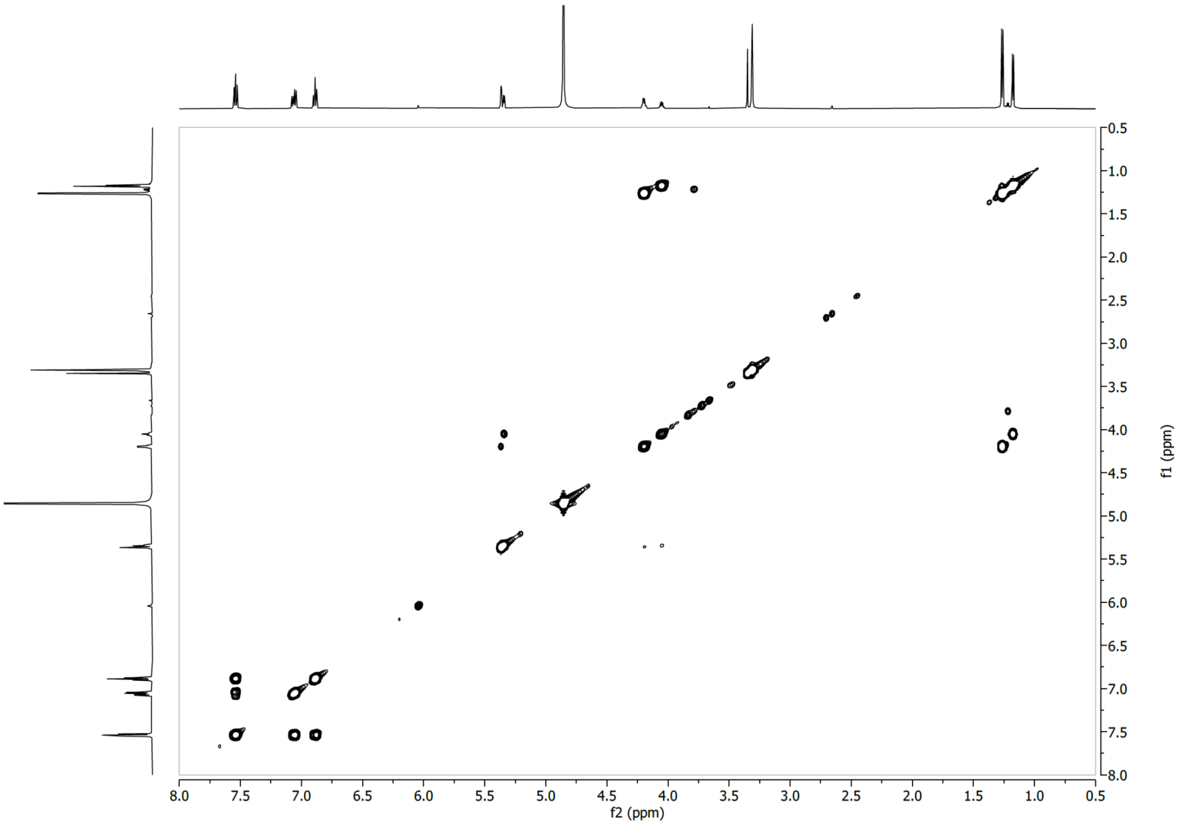

Figure S36. COSY NMR spectrum of compound **6a** and **6b** in CD_3OD

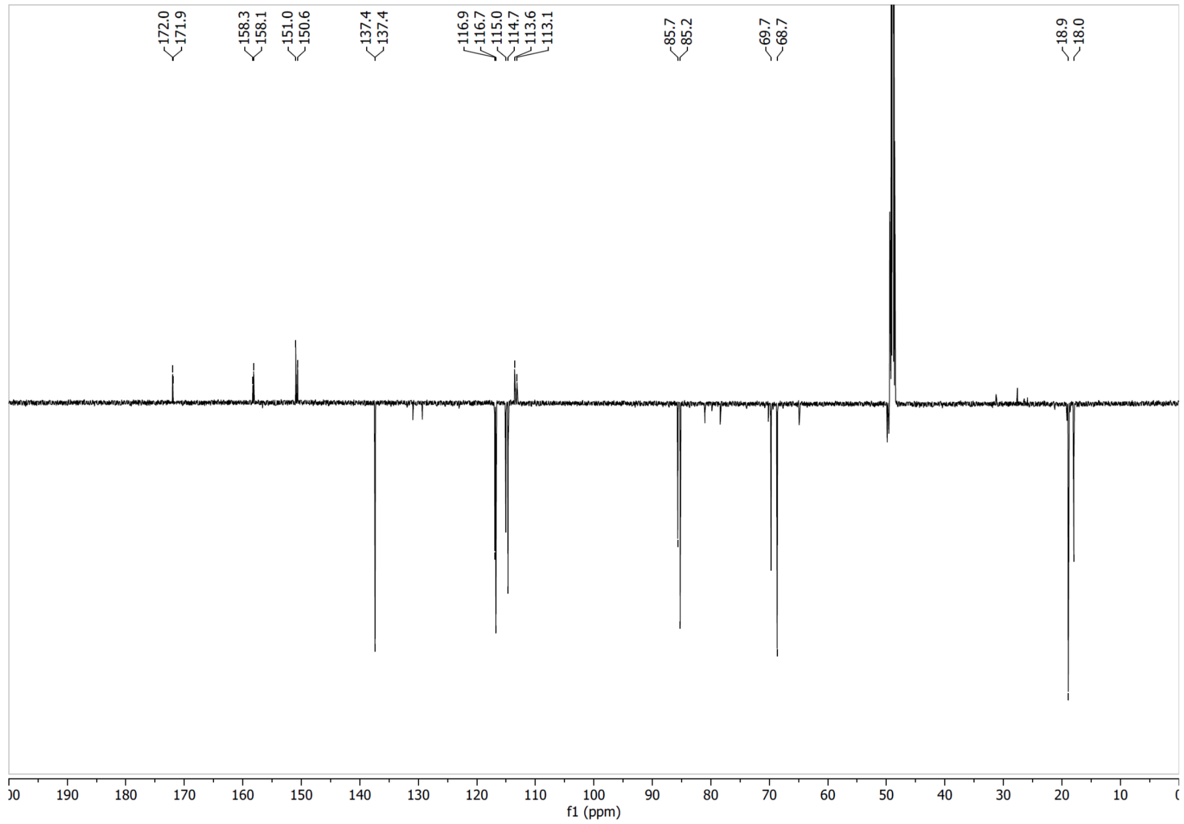

Figure S37. ^{13}C-DEPTQ NMR spectrum of compound **6a** and **6b** in CD_3OD

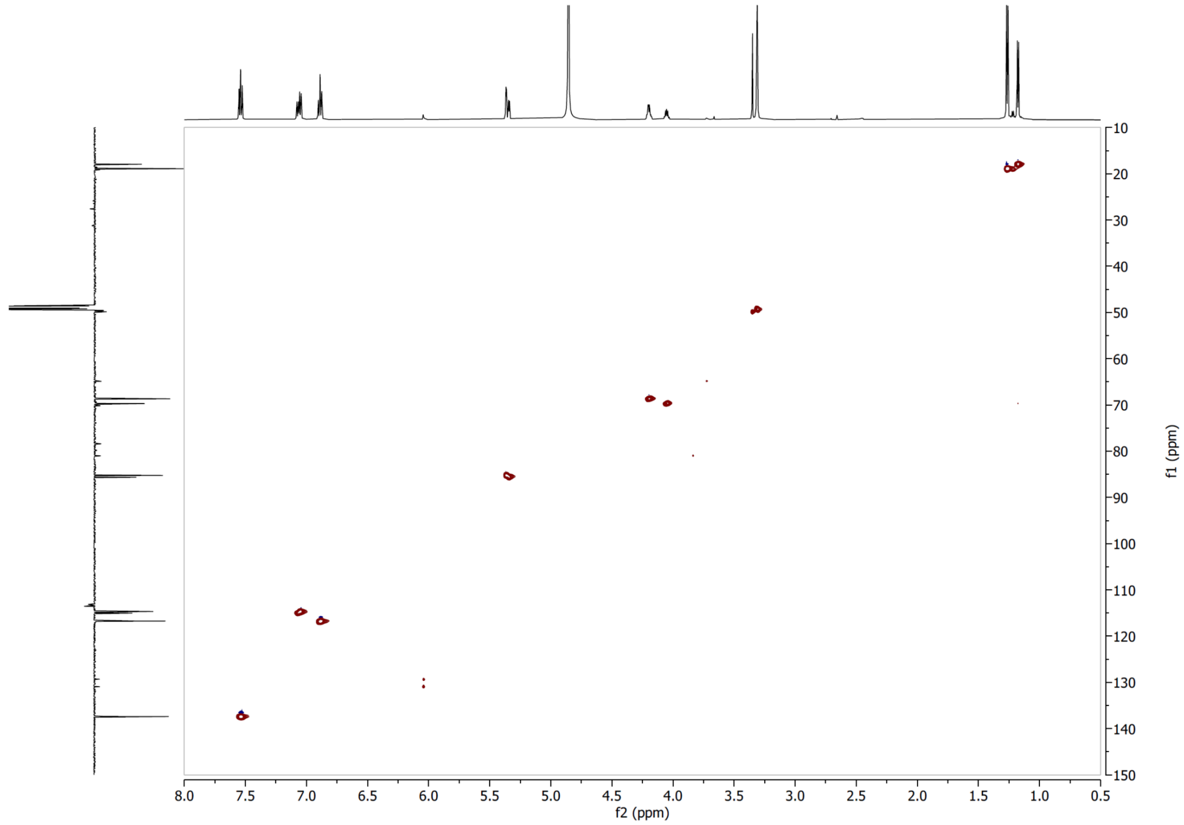

Figure S38. Edited-HSQC NMR spectrum of compound **6a** and **6b** in CD_3OD

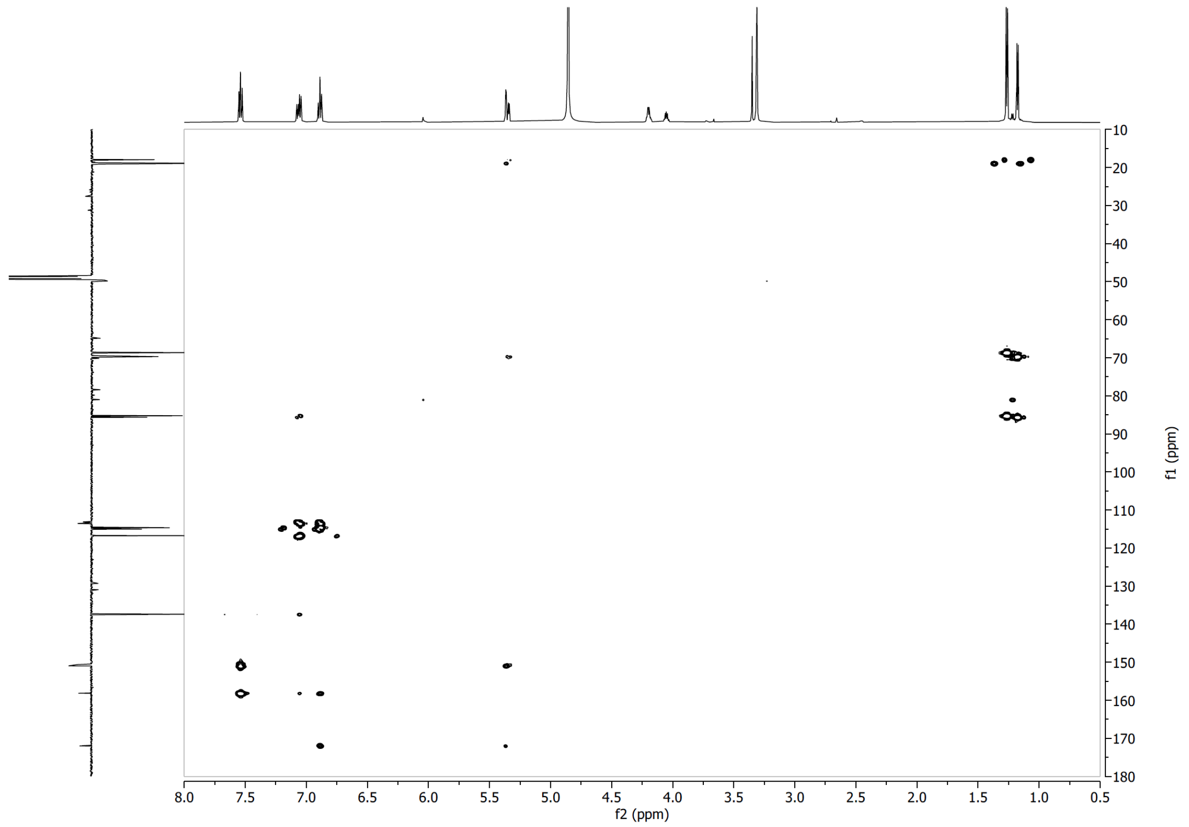

Figure S39. HMBC NMR spectrum of compound **6a** and **6b** in CD_3OD

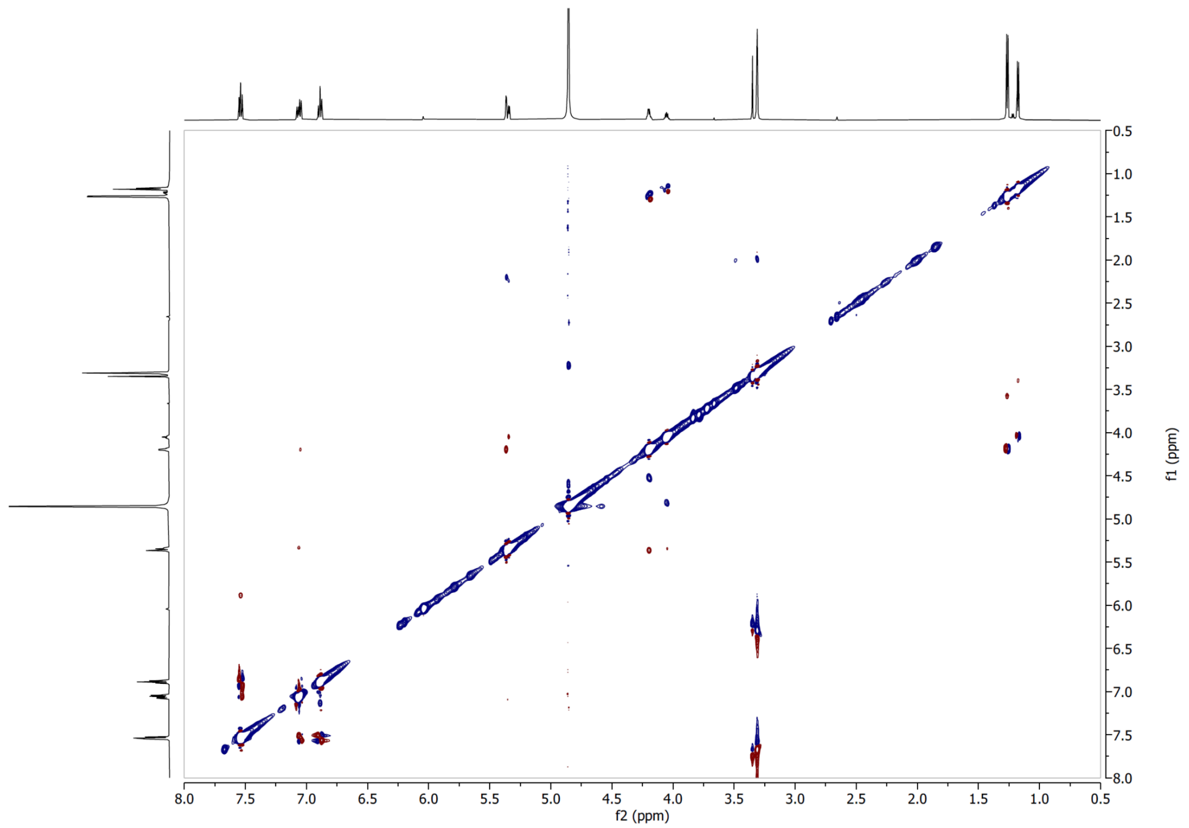

Figure S40. ROESY NMR spectrum of compound **6a** and **6b** in CD_3OD

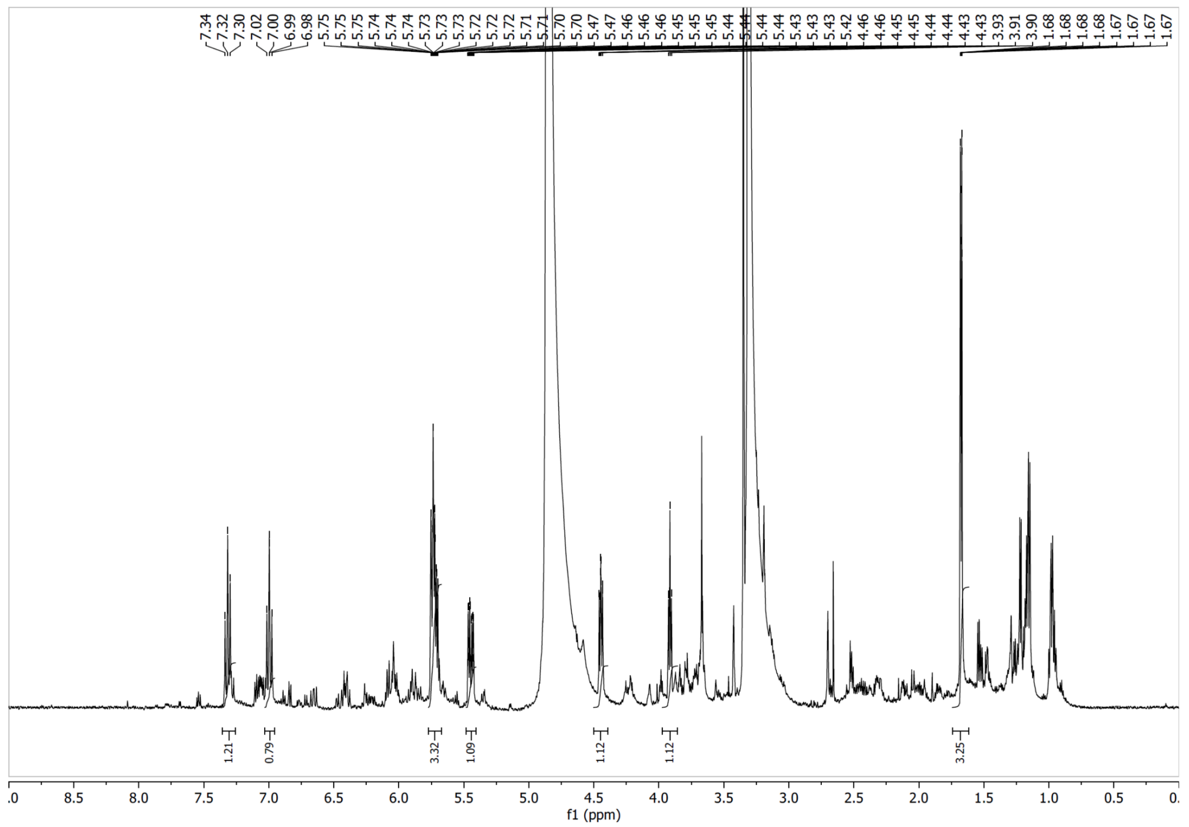

Figure S41. 1H NMR spectrum of compound **7** in CD_3OD

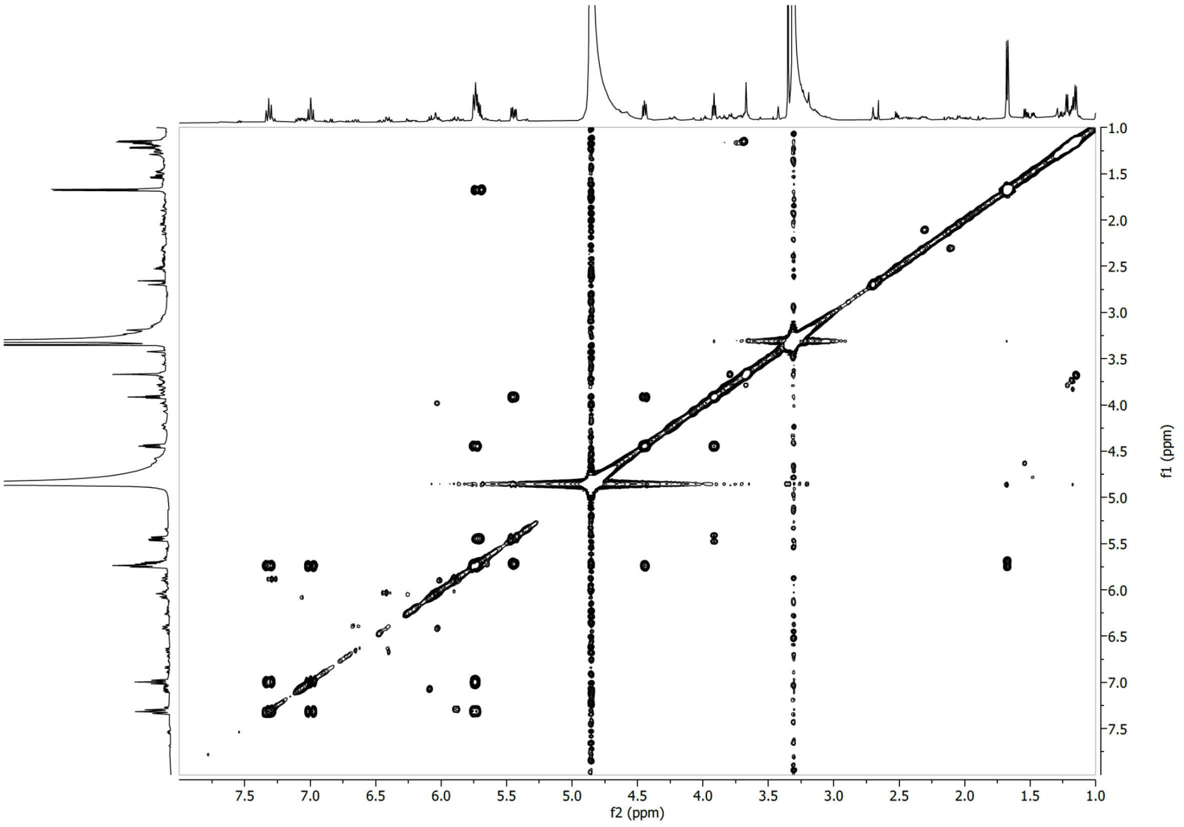

Figure S42. COSY NMR spectrum of compound **7** in CD_3OD

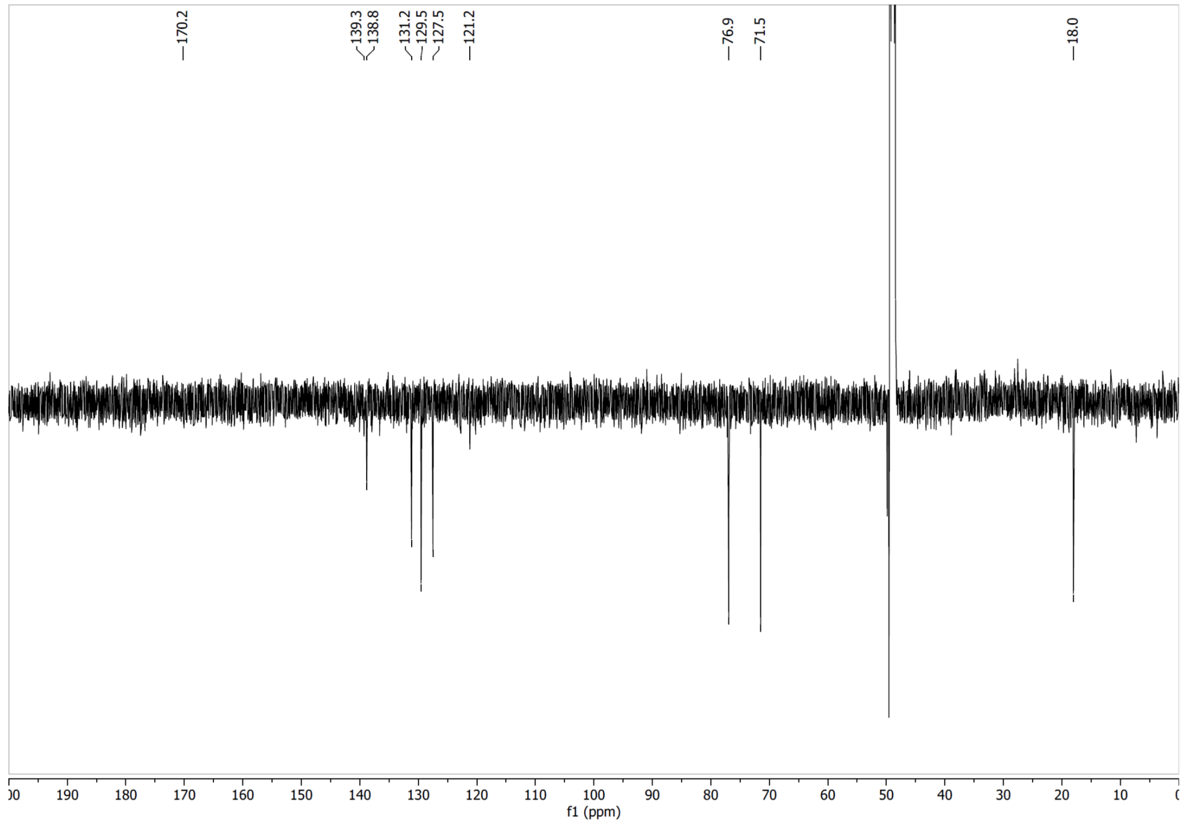

Figure S43. ^{13}C-DEPTQ NMR spectrum of compound **7** in CD_3OD at 151 MHz

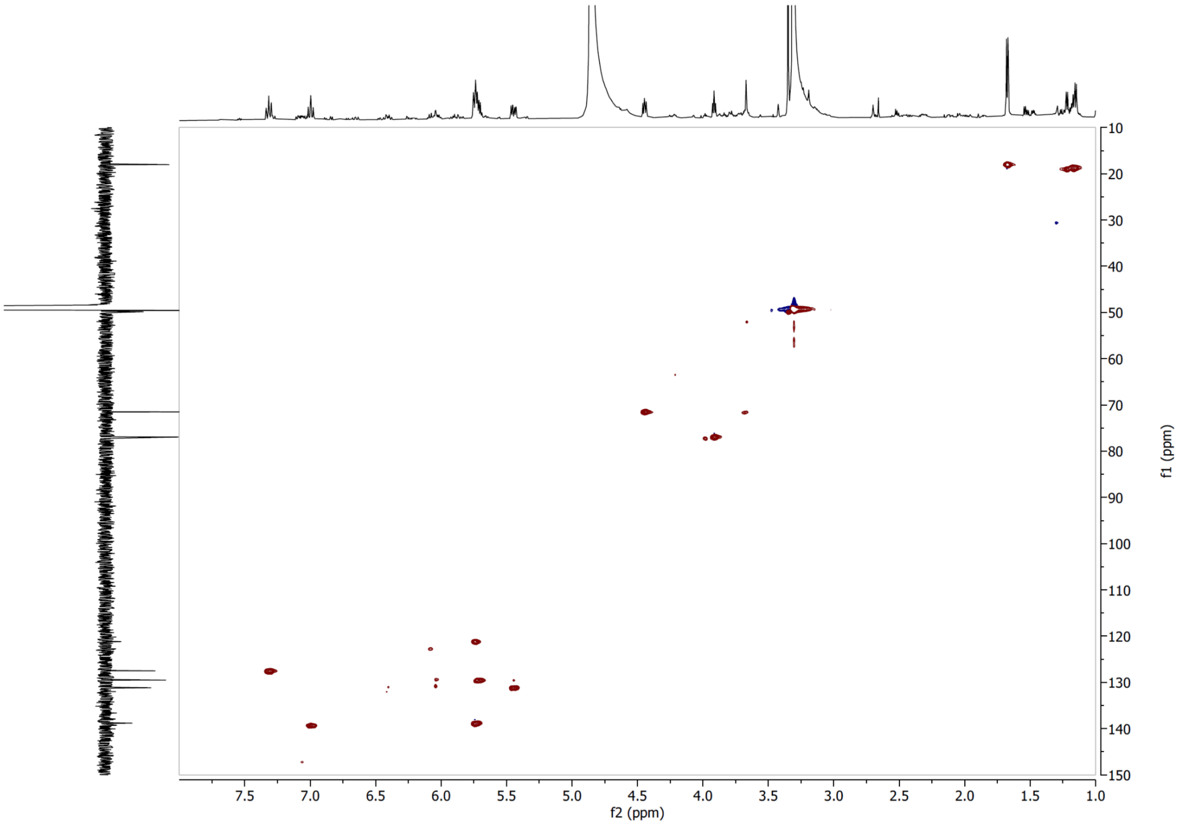

Figure S44. Edited-HSQC NMR spectrum of compound **7** in CD_3OD

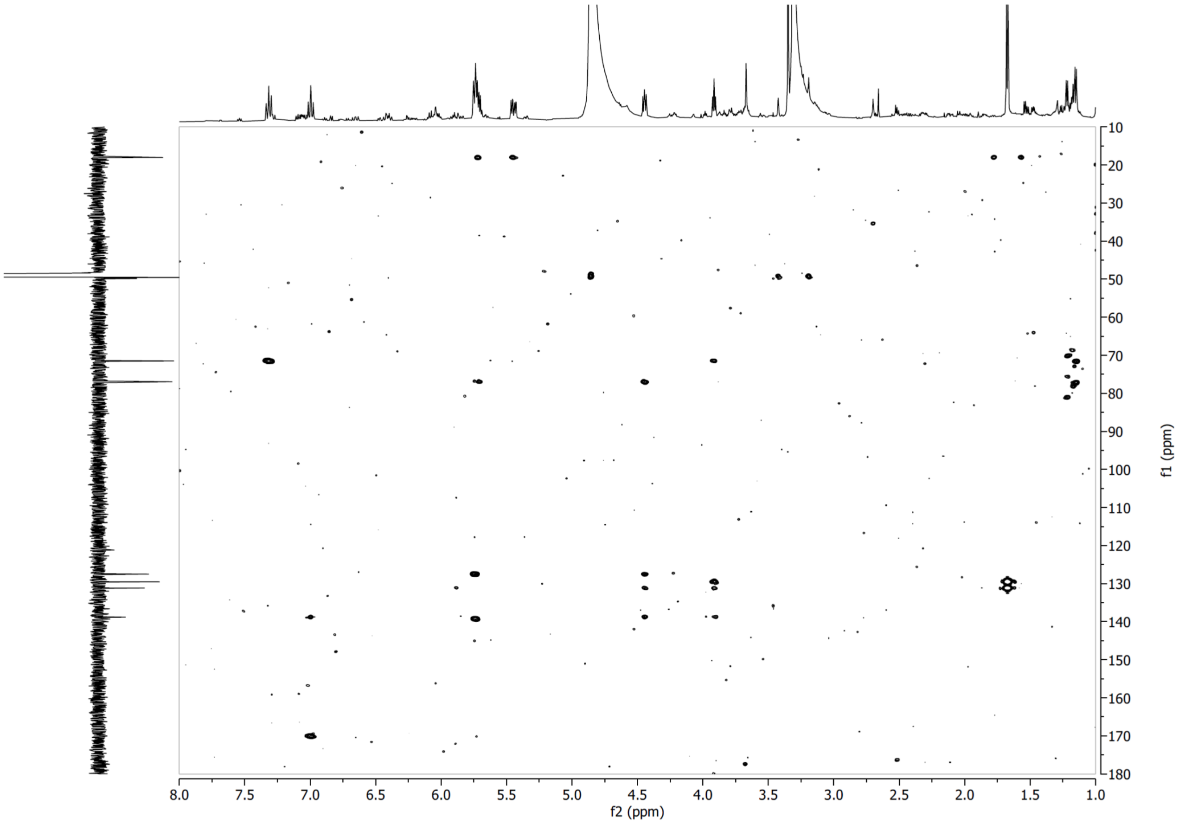

Figure S45. HMBC NMR spectrum of compound **7** in CD_3OD

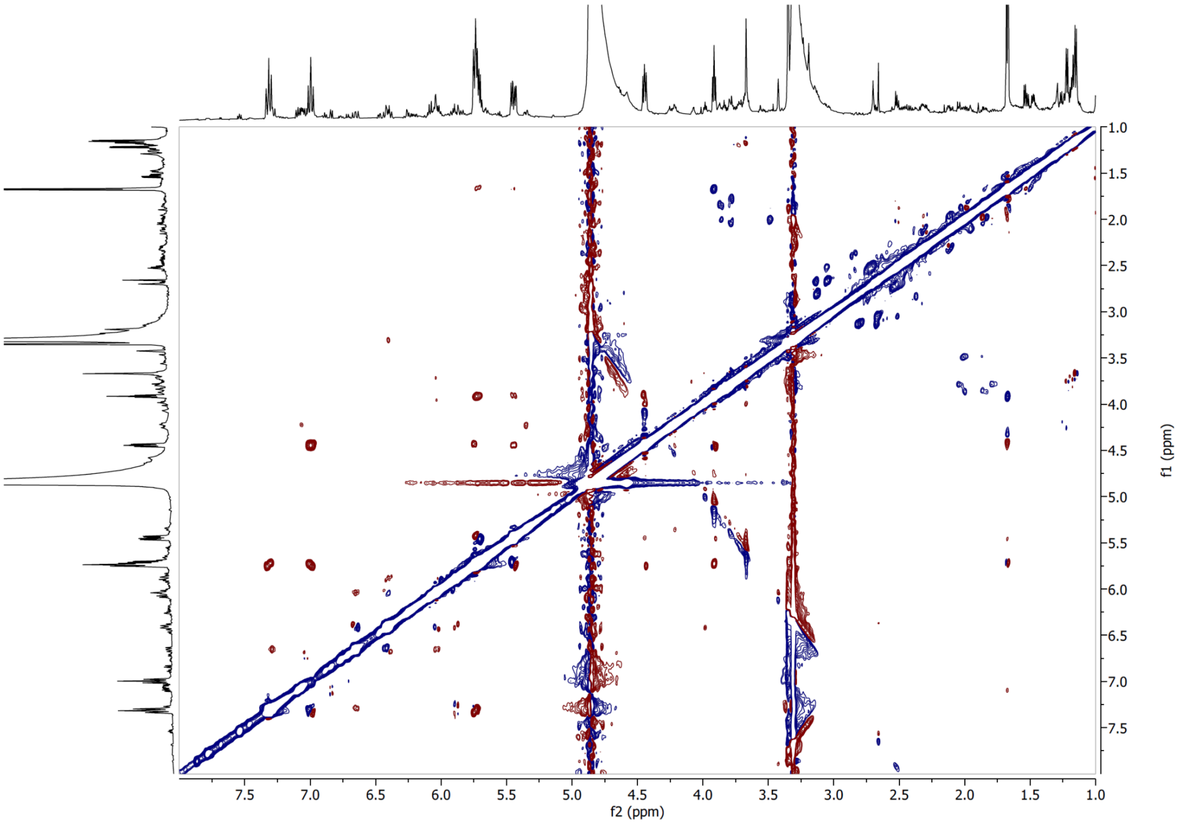

Figure S46. ROESY NMR spectrum of compound **7** in CD_3OD

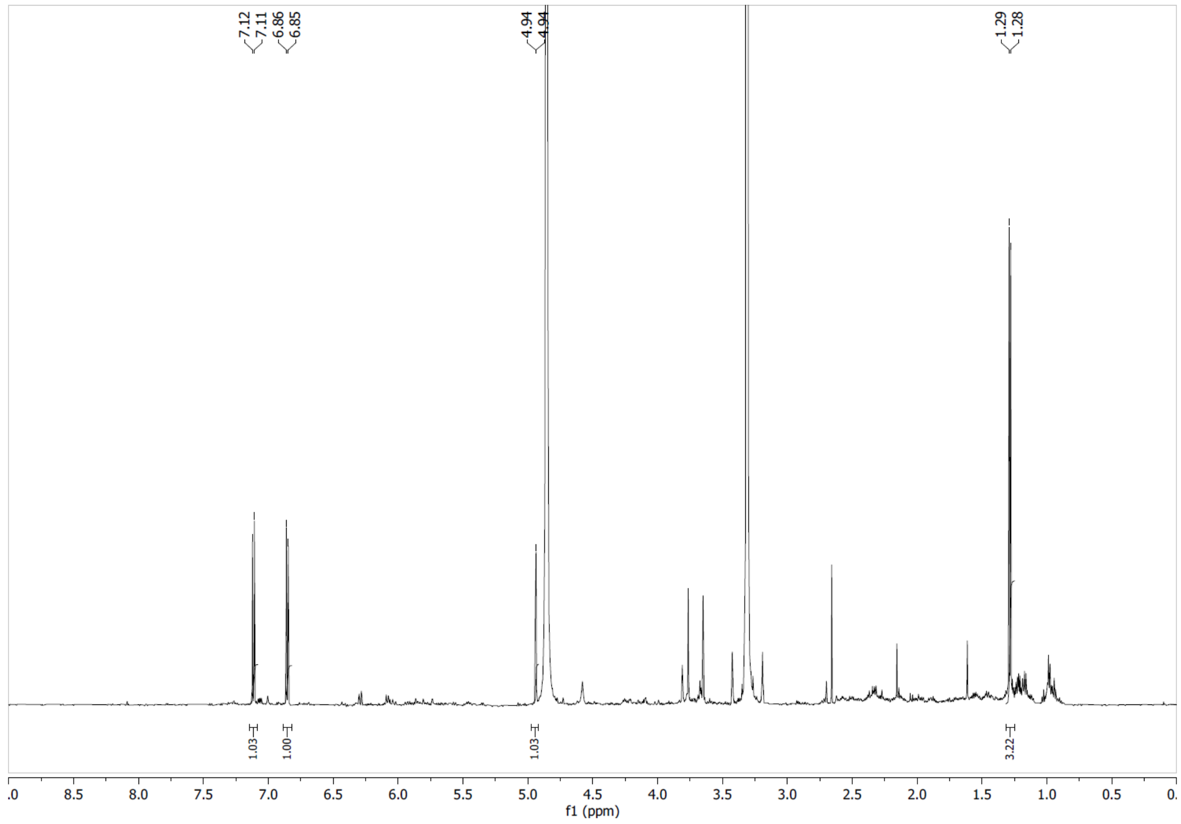

Figure S47. ^{1}H NMR spectrum of compound **8** in CD_3OD at 600 MHz

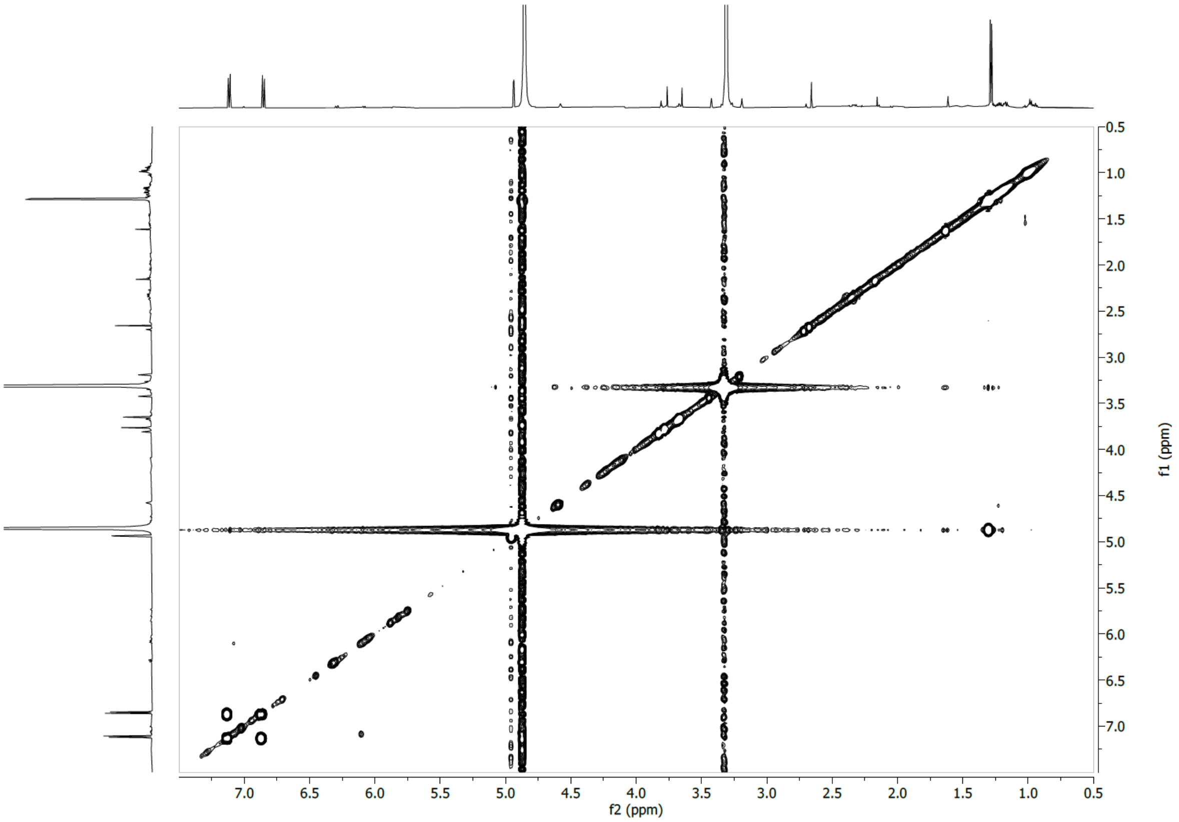

Figure S48. COSY NMR spectrum of compound **8** in CD_3OD

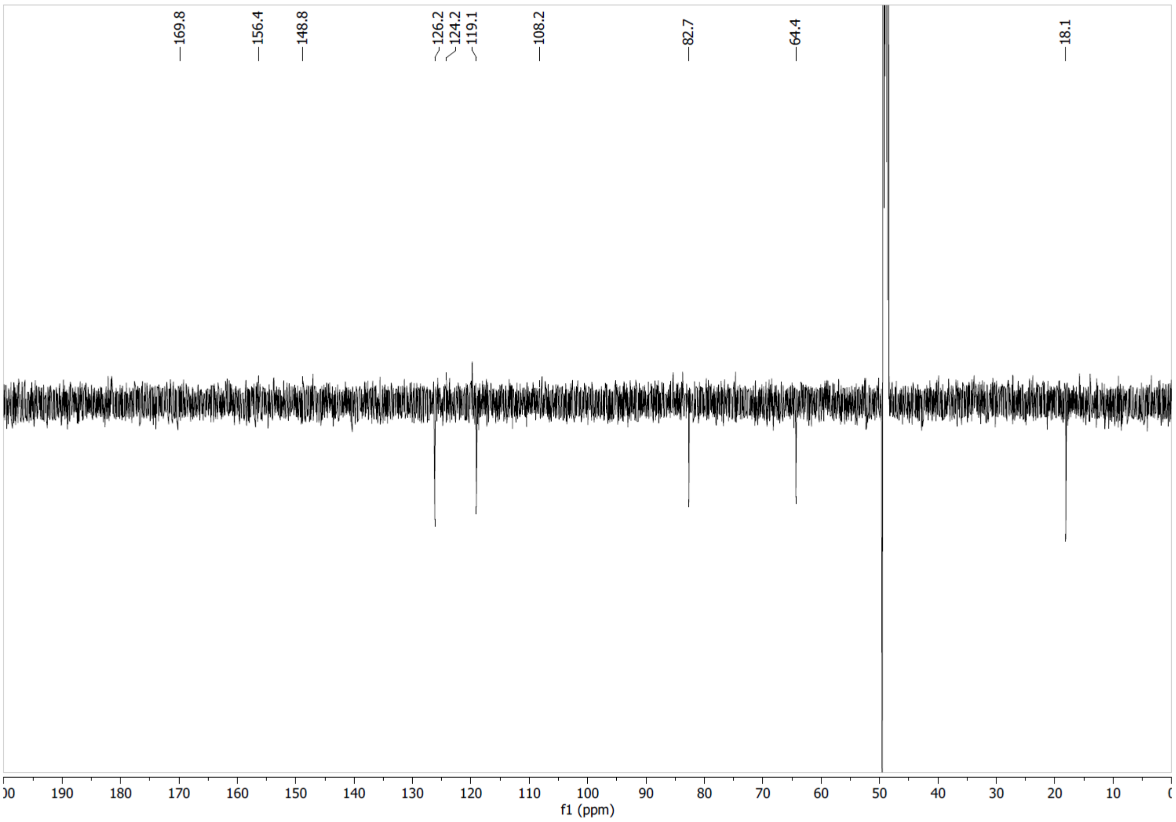

Figure S49. ^{13}C-DEPTQ NMR spectrum of compound **8** in CD_3OD at 151 MHz

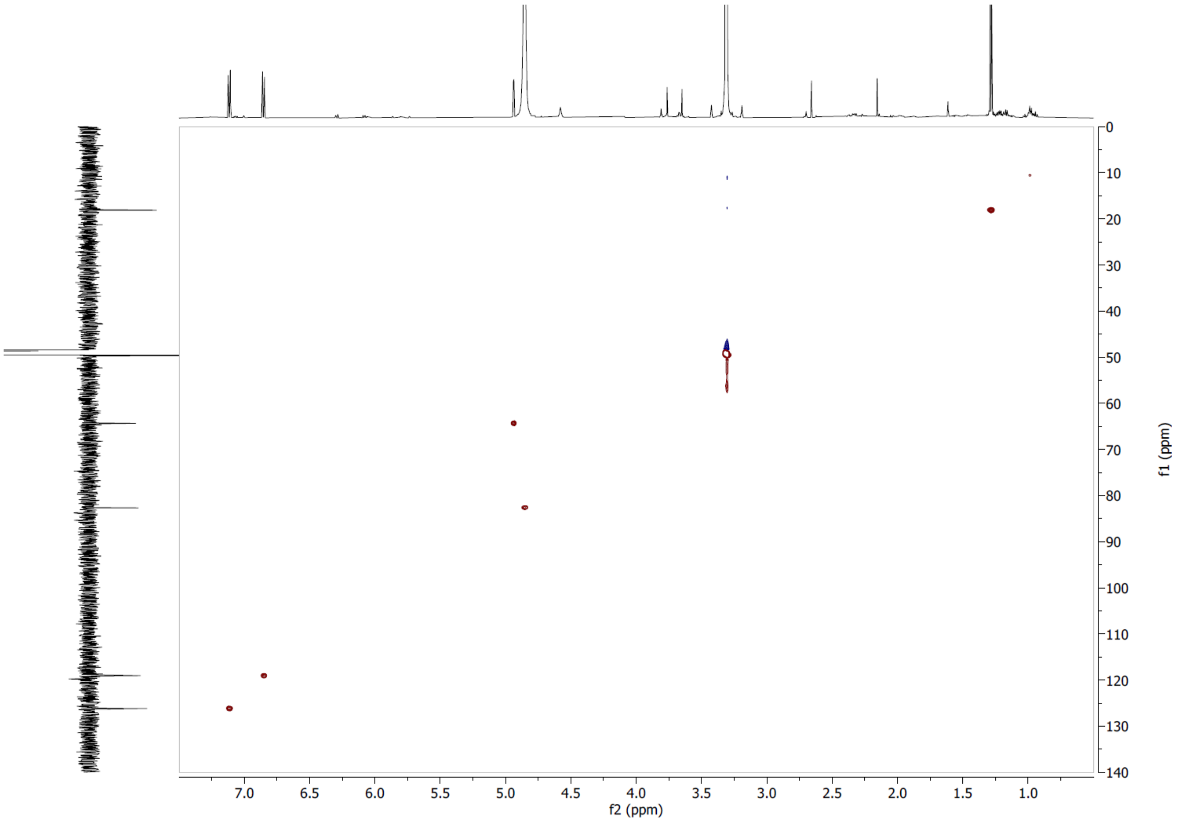

Figure S50. Edited-HSQC NMR spectrum of compound **8** in CD_3OD

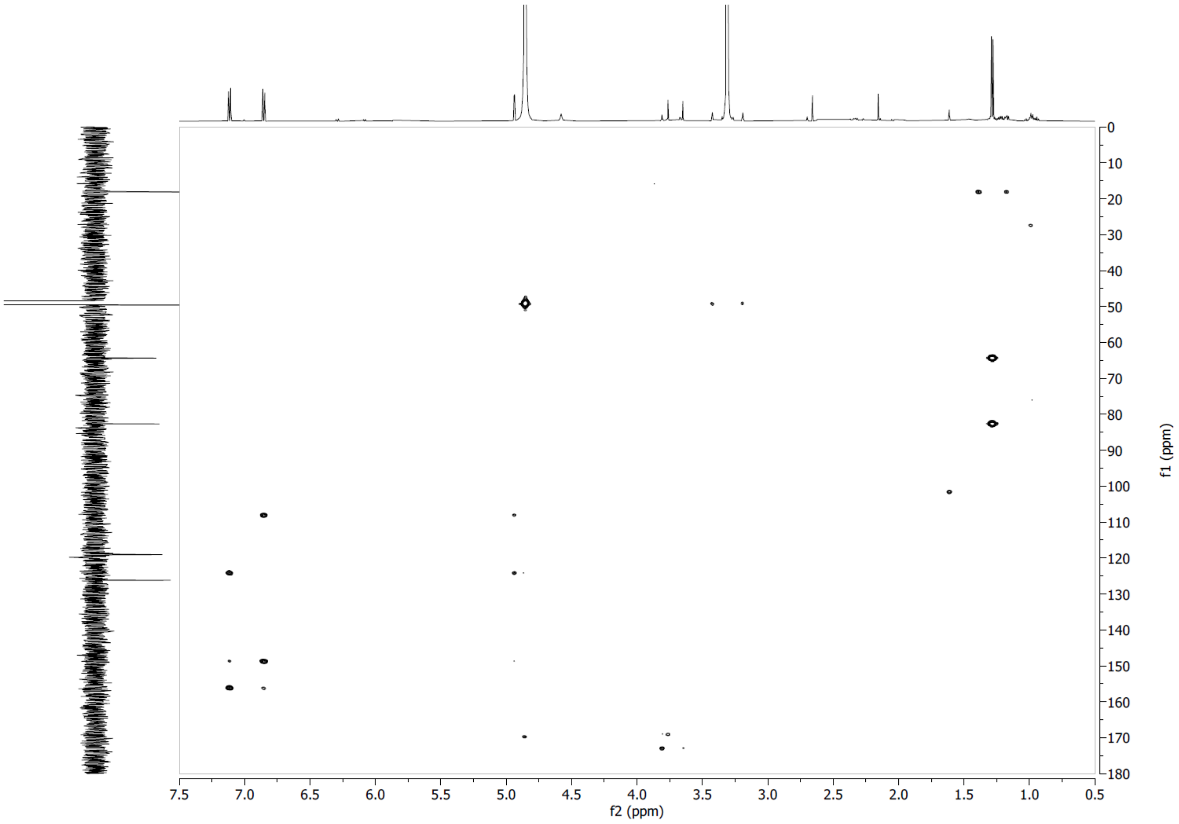

Figure S51. HMBC NMR spectrum of compound **8** in CD_3OD

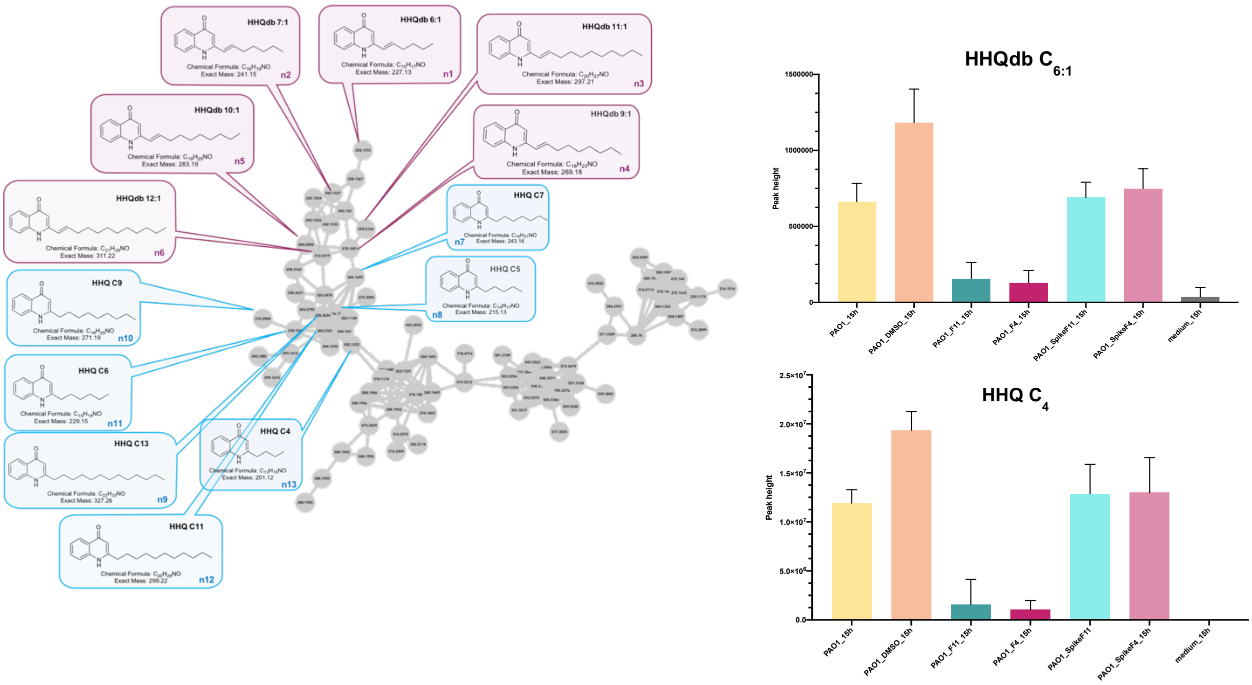

Figure S52. Cluster of alkyl quinolones with annotated HHQs and intensities under the different conditions of the features corresponding to the two HHQs that are significantly decreased with both **2** and **4.**
HHQ: 4-heptyl-4(*1H*)-quinolone, db: double bond**.**

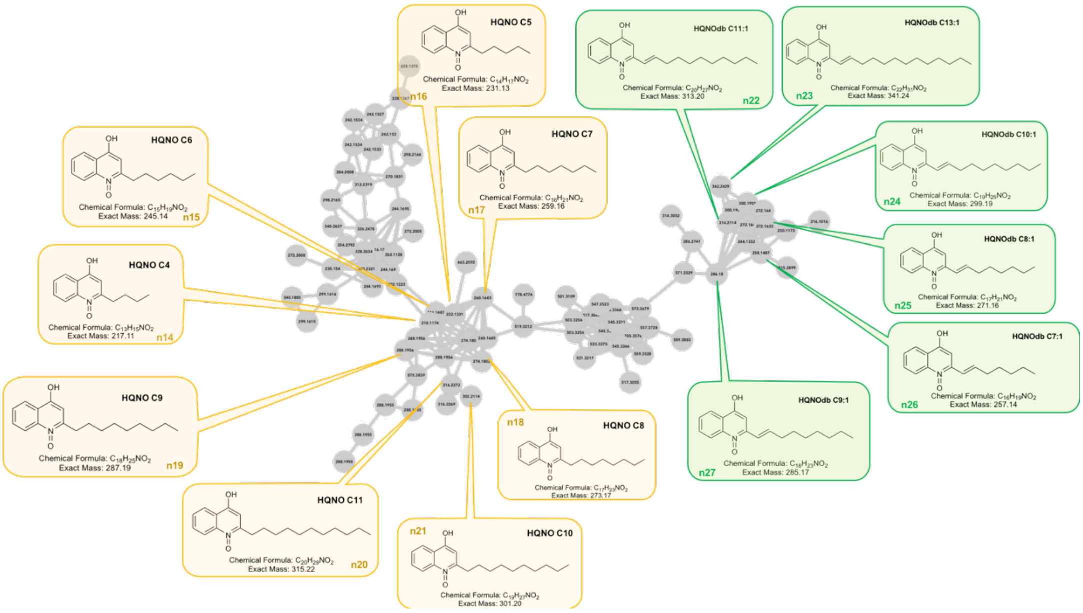

Figure S53. Cluster of alkyl quinolones with annotated HQNOs.
HQNO: 2-heptyl-4-hydroxyquinoline-N-oxide.

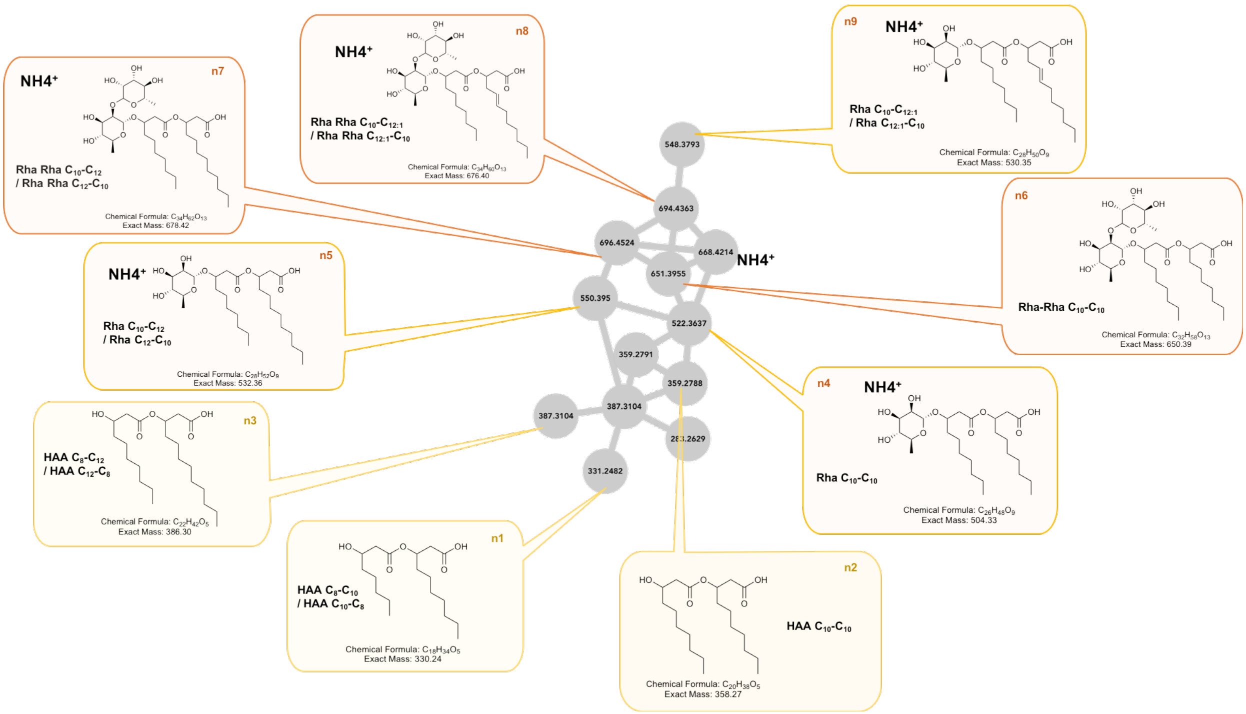

Figure S54. Cluster of rhamnolipids with annotated precursors without rhamnose HAA, mono-rhamnolipids Rha, and di-rhamnolipids Rha-Rha.
HAA: 3-(3-hydroxyalkanoyloxy)alkanoic acids
Rha: rhamnolipid

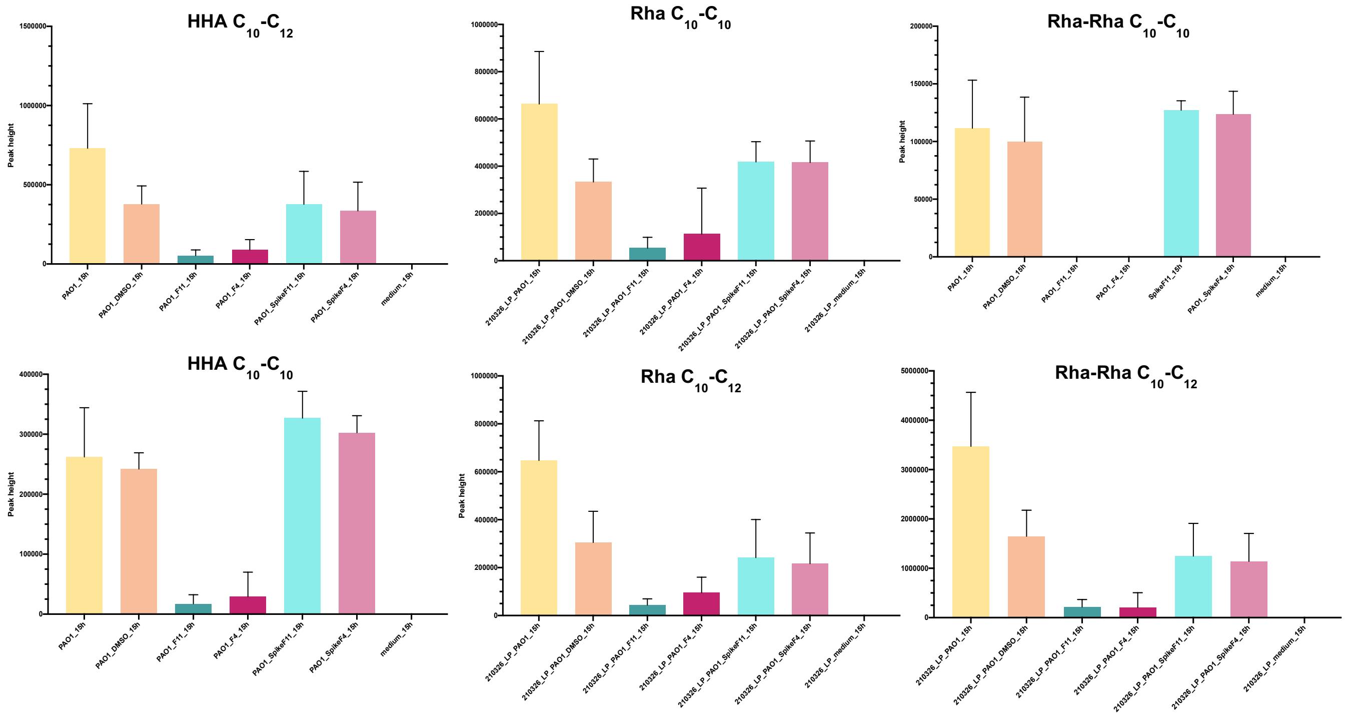

Figure S55. Intensities under the different conditions of the features corresponding to the HAAs and rhamnolipids that are significantly decreased with both **2** and **4.**

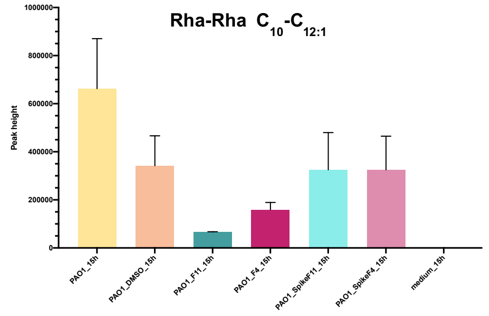

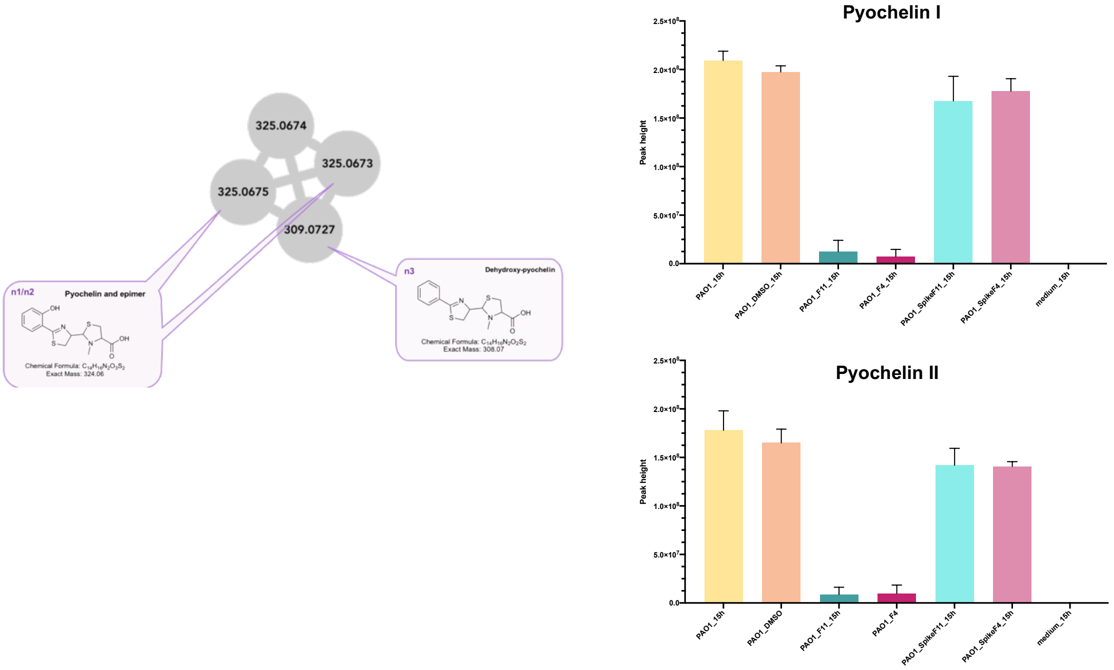

Figure S56. Cluster of pyochelin derivatives with annotated pyochelin I and epimer pyochelin II, dihydroxypyochelin and intensities under the different conditions of the features that are significantly decreased with both **2** and **4.**

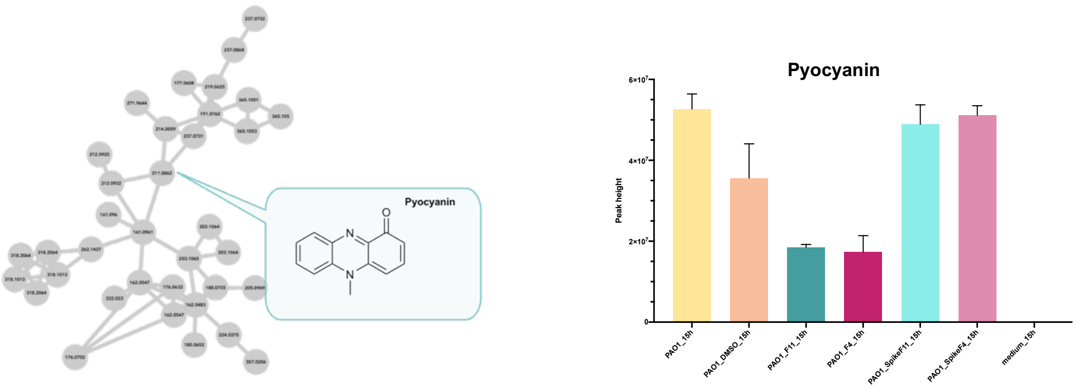

Figure S57. Cluster of pyocyanin derivatives with annotated pyocanin and intensities of the corresponding feature under the different conditions, significantly decreased with both **2** and **4.**

Table S7. Primers used for the qPCR

Gene	Primer forward	Primer reverse
oprF	GGTTACTTCCTGACCGACGA	TCGCTGTTGATGTTGGTGAT
lasB	AAGCCATCACCGAAGTCAAG	GTAGACCAGTTGGGCGATGT
rhlA	CGAGGTCAATCACCTGGTCT	GACGGTCTCGTTGAGCAGAT
pqsA	CAATACACCTCGGGTTCCAC	TGAACCAGGGAAAGAACAGG
pqsR	AACCTGGAAATCGACCTGTG	TGAAATCGTCGAGCAGTACG
